# Existing evidence on antibiotic resistance exposure and transmission to humans from the environment: a systematic map

**DOI:** 10.1186/s13750-022-00262-2

**Published:** 2022-03-12

**Authors:** Isobel Catherine Stanton, Alison Bethel, Anne Frances Clare Leonard, William Hugo Gaze, Ruth Garside

**Affiliations:** 1 European Centre for Environment and Human Health, College of Medicine and Health, Penryn Campus, University of Exeter, Penryn, TR10 9FE UK; 2 College of Medicine and Health, St Luke’s Campus, University of Exeter, Exeter, EX1 1TX UK; 3 European Centre for Environment and Human Health, College of Medicine and Health, Knowledge Spa, University of Exeter, Truro, TR1 3HD UK

**Keywords:** Antibiotic resistance, Health, Colonisation, Infection, Transmission, Exposure, Water, Air, Soil, Food

## Abstract

**Background:**

Antimicrobial resistance (AMR) is predicted to become the leading cause of death by 2050 with antibiotic resistance being an important component. Anthropogenic pollution introduces antibiotic resistant bacteria (ARB) and antibiotic resistance genes (ARGs) to the natural environment. Currently, there is limited empirical evidence demonstrating whether humans are exposed to environmental AMR and whether this exposure can result in measurable human health outcomes. In recent years there has been increasing interest in the role of the environment and disparate evidence on transmission of AMR to humans has been generated but there has been no systematic attempt to summarise this. We aim to create two systematic maps that will collate the evidence for (1) the transmission of antibiotic resistance from the natural environment to humans on a global scale and (2) the state of antibiotic resistance in the environment in the United Kingdom.

**Methods:**

Search strategies were developed for each map. Searches were undertaken in 13 bibliographic databases. Key websites were searched and experts consulted for grey literature. Search results were managed using EndNote X8. Titles and abstracts were screened, followed by the full texts. Articles were double screened at a minimum of 10% at both stages with consistency checking and discussion when disagreements arose. Data extraction occurred in Excel with bespoke forms designed. Data extracted from each selected study included: bibliographic information; study site location; exposure source; exposure route; human health outcome (Map 1); prevalence/percentage/abundance of ARB/antibiotic resistance elements (Map 2) and study design. EviAtlas was used to visualise outputs.

**Results:**

For Map 1, 40 articles were included, from 11,016 unique articles identified in searches, which investigated transmission of AMR from the environment to humans. Results from Map 1 showed that consumption/ingestion was the most studied transmission route. Exposure (n = 17), infection (n = 16) and colonisation (n = 11) being studied as an outcome a similar number of times, with mortality studied infrequently (n = 2). In addition, *E. coli* was the most highly studied bacterium (n = 16). For Map 2, we included 62 studies quantifying ARB or resistance elements in the environment in the UK, from 6874 unique articles were identified in the searches. The most highly researched species was mixed communities (n = 32). The most common methodology employed in this research question was phenotypic testing (n = 37). The most commonly reported outcome was the characterisation of ARBs (n = 40), followed by characterisation of ARGs (n = 35). Other genetic elements, such as screening for *intI1* (n = 15) (which encodes a Class 1 integron which is used as a proxy for environmental ARGs) and point mutations (n = 1) were less frequently reported. Both maps showed that research was focused towards aquatic environments.

**Conclusions:**

Both maps can be used by policy makers to show the global (Map 1) and UK (Map 2) research landscapes and provide an overview of the state of AMR in the environment and human health impacts of interacting with the environment. We have also identified (1) clusters of research which may be used to perform meta-analyses and (2) gaps in the evidence base where future primary research should focus.

**Supplementary Information:**

The online version contains supplementary material available at 10.1186/s13750-022-00262-2.

## Background

The efficacy of antibiotics, which are used to treat and prevent bacterial infections, is critical to human health practices [1]. Currently, this efficacy is being hampered by bacteria evolving mechanisms to resist these drugs (antibiotic resistance) which can lead to an increase in morbidity and mortality from bacterial infections [2].

Antimicrobial resistance (AMR) includes resistance by microorganisms to all chemicals with antimicrobial abilities. This includes bacterial, viral, fungal and parasitic organisms. It is, however, often used interchangeably in the current literature with the term antibiotic resistance (resistance of bacterial species to antibiotic drugs). After discussions with stakeholders (discussed further below in “Stakeholder engagement”), this study focused specifically on antibiotic resistant bacteria (ARB).

In recent years, the impact of AMR, and more specifically, antibiotic resistance, has led to AMR being placed (alongside other threats such as climate change, pandemic influenza and global terrorism) on the UK Risk Register [3, 4]. The World Health Organisation has stated that *“a post-antibiotic era—in which common infections and minor injuries can kill—far from being an apocalyptic fantasy, is instead a very real possibility for the twenty-first century”* [5].

Drug-resistant infections are increasing around the globe [1, 6, 7]. As resistance to commonly used antibiotics increases, the reliance on more expensive last resort antibiotics, with more toxic side effects, also increases to successfully treat infections. However, resistance has already been observed to these last resort antibiotics (e.g. colistin) [8]. Furthermore, the development pipeline for new antibiotics has slowed, with few effective antibiotics being brought to market. This is due to the high investment needed by pharmaceutical companies to develop these products combined with the potential of a small return and limited lifespan of the product before resistance develops [9]. In the past three decades only oxazolidinones and cyclic lipopeptides have come to market as novel antimicrobials [10].

If current trends continue, it is likely that treating infections that are currently easily treatable will become impossible and we will enter an era similar to that before antibiotics were discovered. This will lead to not only an inability to prevent and treat bacterial infections, but will increase the risk of severe morbidity and mortality associated with routine medical procedures [11].

In 2014, the UK government commissioned a report that estimated that by 2050, AMR will be the leading cause of death globally with annual global deaths from AMR increasing from 700,000 in 2014 to 10 million. This report also predicted the impact of AMR on the global economy and estimated a loss of up to 100 trillion US dollars of the world’s GDP (a decrease of 2.5 to 3%) by 2050 [2]. In addition, it is estimated that the NHS already spends £180 million per year on treating AMR infections [11].

To date, there has been a large body of research undertaken that investigates the impact of AMR from a clinical perspective [12–17]. The role of the environment in antibiotic resistance evolution and transmission has, however, received comparatively less attention. The use of antibiotics in humans and agriculture has been implicated in the rise of AMR levels and the subsequent release, dissemination and propagation of ARB/antibiotic resistance genes (ARGs) in environmental settings [18].

The United Nations Environment Programme Frontiers report, published in 2017, listed AMR as one of the most critical global environmental pollution issues [19]. The increasing use of antimicrobial compounds, and the release of these into the environment from anthropogenic sources, has increased the rate of the development of novel resistance mechanisms. These novel resistance mechanisms can be associated with mobile genetic elements which are able to facilitate the dissemination of resistance elements through microbial communities [20]. A large proportion of consumed antibiotics are excreted in an active form. This can be as high as 90% [21] and excretion can occur in both urine and faeces from humans and animals. Waste is released into natural environments such as soil and waterways through managed wastewater discharges and run-off from agricultural land [12]. ARB have been detected in polluted environments, along with measurable antibiotic concentrations that can range from ng/L to µg/L [22]. Studies have shown that even at relatively low concentrations of these drugs (similar to those found in environmental settings), selection pressure ARB/ARGs can occur [23–31]. Introduction of human and animal associated antibiotic resistance and selection pressure from antibiotic residues combined with naturally occurring ARGs means that ARGs and ARBs are found ubiquitously throughout environmental settings [20] although their prevalence varies dramatically depending on levels of pollution [32]. In addition, resistance genes that were originally found in environmental bacteria have the potential to be transferred, by horizontal gene transfer, to human-associated bacteria (including pathogens) causing treatment failure in clinical infections [33, 34]. Furthermore, recent research has shown that while person-to-person transmission is an important route via which people become colonised by ARB, the rates and diversity of transmission are not sufficient to sustain currently observed ARB levels in humans. Therefore, ARB and ARG from non-human sources (including food, contact with animals including wildlife and swimming in natural surface waters) are important sources in the wider community [35, 36].

**Fig. 1:**
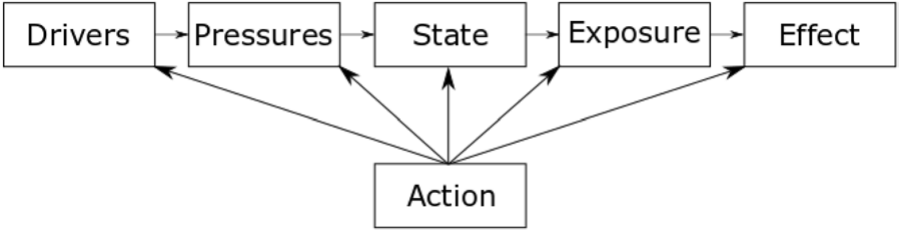
DPSEEA framework. Reproduced from Stanton et al. [38] and originally adapted from Morris et al. [41]

Environmental AMR has been previously mapped using the DPSEEA (Driver–Pressure–State–Exposure–Effect–Action) framework [37–40]. This framework is useful for considering the causal relationships between steps in the pathway that result in high levels of resistance in human healthcare settings, as well as the actions that can be taken at all stages to reduce this effect. The basic DPSEEA framework has been reproduced from the protocol for this map [38] (Fig. 1).

In research investigating environmental AMR: increasing antibiotic use as a result of growing population and increasing resistant infections would constitute the “Driver;” the pollution of the natural environment with antibiotics and co-selecting agents as a result of excretion or inappropriate disposal are “Pressures;” the prevalence or resistance genes and resistant organisms and the concentration of antibiotics relates to the “State;” direct contact with the natural environment is represented by “Exposure” (including consumption or inhalation of both resistant bacteria and resistance genes); the resulting human health outcome of this exposure is the “Effect;” and, finally, any mitigation activity to reduce the human health outcome are the “Action” and may take place at any stage of the framework.

To date, the vast majority of environmental AMR research has been focused on “Pressures” and “State” sections of the DPSEEA model. This research has identified and quantified the release of both chemical and microbial pollutants into the environment, including antimicrobial compounds [42–44] and abundance of ARB [45–47] and ARGs [48–50]. There is also a growing body of evidence investigating the interaction between “Pressure” and “State” (i.e. the concentration of antimicrobials and the resultant effect on ARB and ARGs) [23–31]. In contrast, there is more limited data on the fate of these pollutants in regards to human health (Exposure and Effect).

This study therefore seeks to comprehensively search for, identify and categorise primary research that investigates the links between “State”–“Exposure”–“Effect” in terms of antibiotic resistance. Knowing whether ARB/ARG found in the in environment are transmitted to humans is a key consideration for global policy makers to protect public health. Previous evidence synthesis work in this topic area has been limited. There was one recent systematic review, published in 2020, that investigated different control measures in the environment and their effect on preventing the spread of AMR in the natural environment [51]. However, this study did not investigate the exposure and transmission of ARB to humans from the environment.

We hypothesise that research showing direct transmission (as opposed to implied transmission routes) will focus on where exposure volume is easiest to quantify. This may include: volume of food consumed; volume of drinking water swallowed or volume of water consumed through recreational activities (such as swimming). Tracing direct transmission of ARGs/ARB through define exposure volumes such as these will be easier to quantify than exposure through environments such as soil.

This article describes two linked systematic maps investigating different aspects of exposure and transmission of environmental AMR to humans. We are following ROSES (Reporting standards for systematic evidence syntheses) for systematic map reporting standards (as reported in Additional file 1) and CEE (Collaboration for Environmental Evidence) guidance for methods [52].

## Objective of the review

The aim of this systematic mapping exercise is to provide databases and data on the current evidence relating to environmental AMR and whether this can impact human health. It also aims to identify research gaps and gluts on this topic area.

Our primary research question is: What research evidence is there that humans are exposed to and affected by AMR in the environment?

This research question was subsequently broken down into two further research questions for separate evidence maps. These were:

Map 1: What research evidence is there about ARB exposure and transmission to humans from the environment?

Map 1 aims to explore the “Exposure” and “Effect” sections of the DPSEEA framework. It focuses on studies that investigate quantifiable health outcomes such as colonisation, infection or mortality. In addition, it includes estimated exposure risk assessments (where research focuses on testing the number of resistant bacteria in a particular environment and then estimates exposure by volume of this environment humans consume/ingest/inhale during a particular activity) in humans from the natural environment as this is important to understand potential risk of developing negative human health outcomes through exposure. Both quantifiable and estimated outcomes result from direct contact, inhalation or consumption of ARBs and ARGs from the natural environment.

Map 2: What research evidence is there measuring the prevalence of ARB in the environment in the UK?

Map 2 aims to explore the “State” section of the DPSEEA framework. It is UK based only, as the funder’s scope focuses on UK-based policy research. This map will help to investigate the environments that have been extensively studied and which are under-researched at the time of undertaking. Map 2 will collate studies that investigate the prevalence, percentage or abundance of ARB or ARGs in the natural environment.

### Stakeholder engagement

Prior to developing and initiating the searches, the UK Environment Agency was consulted on the scope of the mapping review, and the review questions. In addition, stakeholders from government organisations, non-government organisations, and industry were invited to comment on the proposed search terms, the sources of grey literature and the inclusion and exclusion criteria. This was done over email and involved members from the organisations: Animal and Plant Health Agency; AstraZeneca; Centre for Environment, Fisheries and Aquaculture Science; Department for Environmental, Food and Rural Affairs; Environment Agency; Food Standards Agency; Joint Nature Conservation Committee; Public Health England; Severn Trent Water and Veterinary Medicines Directorate.

Additionally, a meeting was held in February 2020 with organisations contacted via email together with representatives from: Department of Agriculture, Environment and Rural Affairs; Environment Agency; GlaxoSmithKline; Royal Devon and Exeter Hospital Trust; Severn Trent Water; Welsh Government and Wessex Water. Again, advice was sought on sources of grey literature, the outputs which would be most useful for stakeholders, and any potential means of dissemination of the final outputs through individual organisations.

Finally, a small number of key stakeholders from the Environment Agency were contacted virtually (as a result of the Covid-19 pandemic) and asked to comment on the outputs of the maps, whether they would like to see any other information presented and their opinion of the policy implications of the results.

All suggestions made by stakeholders were discussed by the team to ensure a non-biased inclusion of suggestions from stakeholders.

## Methods

### Deviations from the protocol

The protocol for these two maps was published in 2020 [38]. Deviations from the published protocol were as follows:

1. The inclusion criteria for “outcome” in both maps has been updated in three key ways. First, we have specifically included *intI1* (which encodes a class 1 integron/integrase) as an outcome for both of the maps. The *intI1* gene has been suggested to be a proxy for antibiotic resistance in the environment in a number of publications [53–57] and many studies have, therefore, investigated AMR in the environment using this gene. Second, in Map 2, “abundance” has been included alongside measures of relative abundance (prevalence and percentage) as it is a quantitative measure of AMR in the environment. Finally, we found evidence reporting prevalence, percentage, or abundance of point mutations for Map 2 so these data have been extracted and recorded as an outcome where appropriate, although these were not specifically searched for, unlike with *intI1*. See Table 2 for updated outcomes.
2. In addition to the included articles in Map 1, we also identified a number of articles investigating associations between antibiotic resistance in both the natural environment and human samples. Associations include where authors state in conclusions that their results could infer transmission or an exposure source could pose a risk. These were not included in the final map as co-occurrence of antibiotic resistance in the environment and humans provides poor direct evidence of transmission of resistance from the environment to humans [58]. There were 72 articles identified as “sample comparison” studies. These articles underwent the same screening criteria as the articles included in the final Map 1 database, however they had a number of additional criteria to pass to be included in the final supplementary database:
  - Samples taken from both humans and the environment had to be geographically and temporally related (samples had to be taken in the same year and had to be from the same region of a country). For example, this resulted in studies that investigated historical patient data and compared it to current environmental sampling to be excluded. In addition, studies investigating publicly available global metagenome databases, for example, were excluded because of lack of geographical or temporal links. Finally, studies that only specified that samples came from a country, and not a specific region within the country, were excluded as this was deemed to not be a sufficiently robust link between the two types of samples.
  - With these types of studies, the directionality of transmission is hard to identify without knowledge of exposure events and/or routes. If the authors of the identified studies suggested that the directionality of transmission was from humans to the environment, studies were excluded. If the authors suggested that the directionality of transmission was from the environment to humans or there was no suggestion of direction, studies were included in the supplementary database.

No metadata have been extracted from these studies but a list of the studies that fit the inclusion criteria, except for exposure route and correct directionality of transmission, are included as Additional file 2.

### Search for articles

#### Search terms and strings

Medline via OvidSP was used to develop the searches undertaken in bibliographic databases for both Map 1 and Map 2 by the information specialist (AB). This was undertaken through a process of scoping, checking with the other authors and testing using known includable articles (Additional file 3). Final search strings for the searches for both maps can be found in Additional file 4.

#### Search sources

The search was then adapted for use across multiple databases: AGRIS (via FAO); BIOSIS Citation Index (via Web of Science 1990–present); CAB Abstracts (1973–present); Environment Complete (via EBSCOhost, 1888–present); Epistemonikos (via website: https://www.epistemonikos.org/en/), ProQuest Dissertations and Theses Global (via ProQuest, 1861–present); Explore (via the British Library); Global Health (via OvidSP, 1973–present); GreenFILE (EBSCOHost), SCOPUS (1788–present); Medline (1946–present) and Web of Science Core Collection (SCI Expanded 1990–present; SSCI 1956–present; A&HCI 1975–present; CPCI-S 1990–present; CPCI-SSH 1990–present; ESCI 2015–present) (via Web of Science). Databases were limited by search dates depending on the map (please see details below).

### Supplemental searches

Additional articles were identified through supplementary search methods for both maps.

For Map 2, the following organisational websites recommended by stakeholders were searched: Centre for Environment, Fisheries and Aquaculture Science; Environment Agency; Scottish Environmental Protection Agency; Department of Environment, Food and Rural Affairs; Animal and Plant Health Agency; Veterinary Medicines Directorate; Public Health England; Control of Antimicrobial Resistance Scotland; Health Protection Scotland and Welsh government.

For Map 2, author searches were undertaken in Web of Science Core Collection and SCOPUS for researchers (Gaze, W.; Wellington, E.; and Amos, G.C.). Google Scholar searches were also carried out using Publish or Perish. All of the results from the author searches and google searches were downloaded into Endnote along with the database results and duplicates removed.

#### Search limits

Only studies published in English from both the published and grey literature were considered for both maps, as a result of limited resources and as a result of Map 2 being relevant to UK policy. Date limits were used to cover research articles published from 2009 to present and from 2005 to present for Map 1 and Map 2, respectively. For Map 1, dates prior to 2009 were excluded as eligible research prior to this date was unlikely. For Map 2, the search was extended to include studies from 2005 onwards, as this was when interested in this topic started to increase. The different choices in date selection was agreed upon after expert consultation. For Map 2, to focus on studies in the UK only, a modified version of the MEDLINE Ovid UK search filter [59] was used for the database searches.

#### Estimating comprehensiveness of the search

For both maps, the comprehensiveness of the search was validated by checking that key papers that were known to be included in the final maps were identified by the searches. There were 8 and 5 known included papers for Map 1 and Map 2, respectively. The final searches for both Map 1 and Map 2 were able to identify all key publications. These papers, which were known to be eligible for the map, were provided by topic experts on the mapping team (AL and WG). A list of references for all key publications for both maps can be found in Additional file 3.

#### Search update

These searches were run between October 2019 and February 2020 and have not been updated.

### Article screening and study eligibility criteria

#### Screening process

Title and abstract screening was undertaken by reviewers IS, AL, RG and AB independently with 10% double screening. For Map 1, there was initially 91.8% agreement and for Map 2, 96.1% agreement. All of these were discussed between all reviewers to resolve the disagreements, and refine understanding of the eligibility criteria, before continuing to full text screening. Full text screening was undertaken by IS, AL and RG. As with title and abstract screening, screening for full text was undertaken by authors independently with 10% of full text articles double screened and, again, all disagreements were discussed between all the authors. For articles which were written by the authors of this publication (AL, RG and WG), other members of the screening team (IS and AB) were able to screen or double screen where appropriate.

#### Eligibility criteria

##### Map 1

Table 1 shows the inclusion and exclusion criteria and the justification for Map 1. This has been updated since the publication of the protocol as specified above in “Deviations from the protocol.” The eligibility criteria are expressed in a PEO format (Population, Exposure, Outcome) with both exposure source and exposure route considered important for this question. In addition, we included the following study designs: systematic reviews (reviews with a structured question, search strategy, defined inclusion criteria, quality appraisal and synthesis strategy), experimental studies (randomised exposure trials), observational studies (prospective/retrospective cohort studies, cross-sectional studies, case studies and case series) and modelling studies (for example quantitative microbial risk assessments).

**Table 1:** Eligibility criteria for Map 1

	Inclusion	Exclusion	Justification
Population	Adults, Children	Non-humans (e.g. animals, plants)	Evidence of ARB/ARG transmission to humans from the environment is of interest to relevant stakeholders
Exposure sources	Meat from wild animals, including shellfish (bivalve molluscs, lobster, crab, etc.), fin fish, game)	Meat and animal products from commercially produced animals including fish, shellfish (including shrimp), poultry (including pheasants), pigs, cows, sheep etc., and products including honey, milk, eggs)	While food is produced in the environment, practices during commercial production and processing (e.g. antibiotic use, through poor hygiene in preparation and handling of food) might be the sources of ARB, rather than from the environment Wild animals consumed for their meat are of interest as sources of AMR are more likely to be from the environment. Bivalve molluscs are grown in the environment and are filter-feeders, concentrating contaminants in the environment, and this meat is typically eaten raw or lightly cooked. Shrimp are intensively raised in some parts of the world, and antibiotic usage is poorly regulated. Likewise, pheasants are reared on high levels of antibiotics and then released into the environment [81]
	Plants that are consumed raw (including salad, fruit etc.)	Plants that are always consumed cooked (including grains etc.)	Plants consumed raw pose are more likely to result in transmission of ARB from plants to humans
	Water in the environment (including water used in crop irrigation; aquaculture; ambient surface waters used for recreation; drinking water and wastewater from domestic and industrial sources)	Water from chlorinated swimming pools and spas	Water in swimming pools and spas are treated to remove pathogenic microorganisms, and are not considered the environment
	Soil including that conditioned with faecal matter (sludge/slurry/manure etc. or irrigated with waste water). Exposure may be through activities such as farming, gardening, leisure activities such as playing etc.)	N/A	
	Outdoor air (may contain dust, water droplets etc.)	Studies that have collected air from indoor environments	ARB/ARGs in outdoor environment are more likely to be from natural sources
	Contact with animals or their faeces	Pets, companion animals and commercially produced livestock	ARB/ARGs in/on wild animals are more likely to be from the environment. Those on pets, companion animals and commercially produced animals might be due to antimicrobial usage during animal rearing, for example raw food diet in companion animals is associated with carriage of ARB [82]. Exposure and transmission from pets, companion animals and commercially produced livestock are not of specific interest to relevant stakeholders
Exposure routes	Consumption/ingestion; Inhalation Direct contact		
Outcomes	Mortality caused by infection with ARB, bacteria harbouring ARG(s) or bacteria harbouring *intI1/*Class 1 integron - Infection with ARB, bacteria harbouring ARG(s) or bacteria harbouring *intI1*/Class 1 integron - Colonisation by ARB, bacteria harbouring ARG(s) or bacteria harbouring *intI1*/Class 1 integron - Estimated or measured risk of exposure to ARB, bacteria harbouring ARG(s) or bacteria harbouring *intI1*/Class 1 integron	Infections caused by fungi, parasites or viruses	While fungal, parasitic and viral infections resistant to antimicrobials are of interest to relevant stakeholders, ARB are a priority for regulators Resource constraints mean other types of AMR organisms will not be included Although this is not a human health outcome it represents this risk of exposure that could result in a human health outcome and is, therefore, deemed important to include

*ARB* antibiotic resistant bacteria, *ARG* antibiotic resistance gene, *AMR* antimicrobial resistance

##### Map 2

Table 2 shows the inclusion and exclusion criteria and the justifications for Map 2. This has been updated since the publication of the protocol as specified above in “Deviations from the protocol.” The eligibility criteria has been expressed in a PEO format with both exposure source and exposure route considered important for this question. In addition, we included the following study designs: systematic reviews (reviews with a structured question, search strategy, defined inclusion criteria, quality appraisal and synthesis strategy) and environmental surveillance studies.

**Table 2:** Eligibility criteria for Map 2

	Inclusion	Exclusion	Justification
Population	Bacteria	Fungi, parasites, viruses	ARBs are a priority interest for relevant stakeholders. Resource constraints mean other types of AMR organisms will not be included
Exposure sources	As in Table 1	As in Table 1	As in Table 1
Exposure routes	Exposure to ARB		
Outcomes	Prevalence/percentage/abundance of ARB Prevalence/percentage/abundance of ARGs Prevalence/percentage/abundance of *intI1*/Class 1 integron Prevalence/percentage/abundance of point mutations	Presence of ARB/ARGs/*intI1*/point mutations with no quantification	

*ARB* antibiotic resistant bacteria, *ARG* antibiotic resistance gene, *AMR* antimicrobial resistance

### Study validity assessment

Studies were not assessed for their validity. Data coded (such as study designs), however, may indicate some aspects of study validity.

### Meta-data extraction and coding strategy

Pilot data extraction tables were developed by all the authors and trialled with known included articles prior to extraction of full text for both maps. As a result of this trialling, the authors were able to make adjustments to the data extraction spreadsheet. This included what information to extract from publications, for example. Excel was used for extracting the data and creating the two map databases. All data from included publications was extracted by one author (IS) to ensure consistency between extractions. Trialling extractions of a small number of the known include articles was undertaken by IS for both Map 1 and 2 and cross checked by all other authors (AL, RG, AB, WG) before proceeding to extracting all included articles. If there was uncertainty during the extraction process of the included articles, other authors were consulted throughout this process. IS has not authored any papers included in either of the maps and authors (WG, AL, RG) who had authored included articles were not consulted on these specific articles, instead AB was consulted. If data in the publications were unclear or missing, authors of the study were contacted to attempt to obtain these data.

For Map 1, data extracted were: citation details; year published; study type, exposure source main, exposure source sub-category; outcome; species; study site; date conducted; longitude; latitude and hyperlink.

For Map 2, data extracted were: citation details; year published; exposure source main; exposure source sub-category; outcome; methods; sample comparison; sampling site(s); species/diversity information; latitude; longitude; study type and hyperlink.

Every reasonable attempt was made to find full text articles for screening which included searching the University of Exeter Library holdings, extensive internet searching and applying to the British Library for copies but a small number were not obtained, 30 for Map 1 and 6 for Map 2 (Additional file 5).

### Data mapping method

Results are presented graphically using EviAtlas [60] to display heatmaps, bar graphs and a geographical map for each of the two systematic maps. Heatmaps were used to identify knowledge gaps and gluts in the two evidence maps.

For both maps, meta-data (e.g. methodology, location, date) were extracted. Methodology, bacterial species and date of publication were presented as bar graphs. Location of studies (either reported or estimated based on author affiliations) were displayed on a geographical map.

For Map 1, exposure environments were classed as air, animal, food, other, sediment, soil, and water. Each of these were further categorised into 29 subcategories (please see subcategories in “Results” section). Exposure route categories were: consumption/ingestion, direct contact, inhalation, and no information. Four broad categories of health outcome were used: colonisation, exposure risk, infection and mortality. Heatmaps were produced to display the number of studies reporting data on environmental exposure and health outcome categories. In addition, the species of bacteria reported by each study were recorded where available, and displayed as (bar chart/heatmap).

For Map 2, the same classifications for environments as Map 1 were used, and these were categorised into 40 subcategories (please see subcategories in “Results” section). Again, heatmaps were produced to display the number of studies reporting data on measures of antibiotic resistance in different environmental compartments. The species of bacteria investigated were recorded, where reported, and displayed as a bar chart. Results were discussed with all authors to ensure unbiased reporting of the narrative synthesis of results and, where authors of this publication had been involved in studies reported in the maps (AL, RG, WG), the remaining authors (IS, AB) interpreted and reported on these publications.

## Review findings

### Map 1: What research evidence is there about ARB exposure and transmission to humans from the environment?

#### Description of review process

**Fig. 2:**
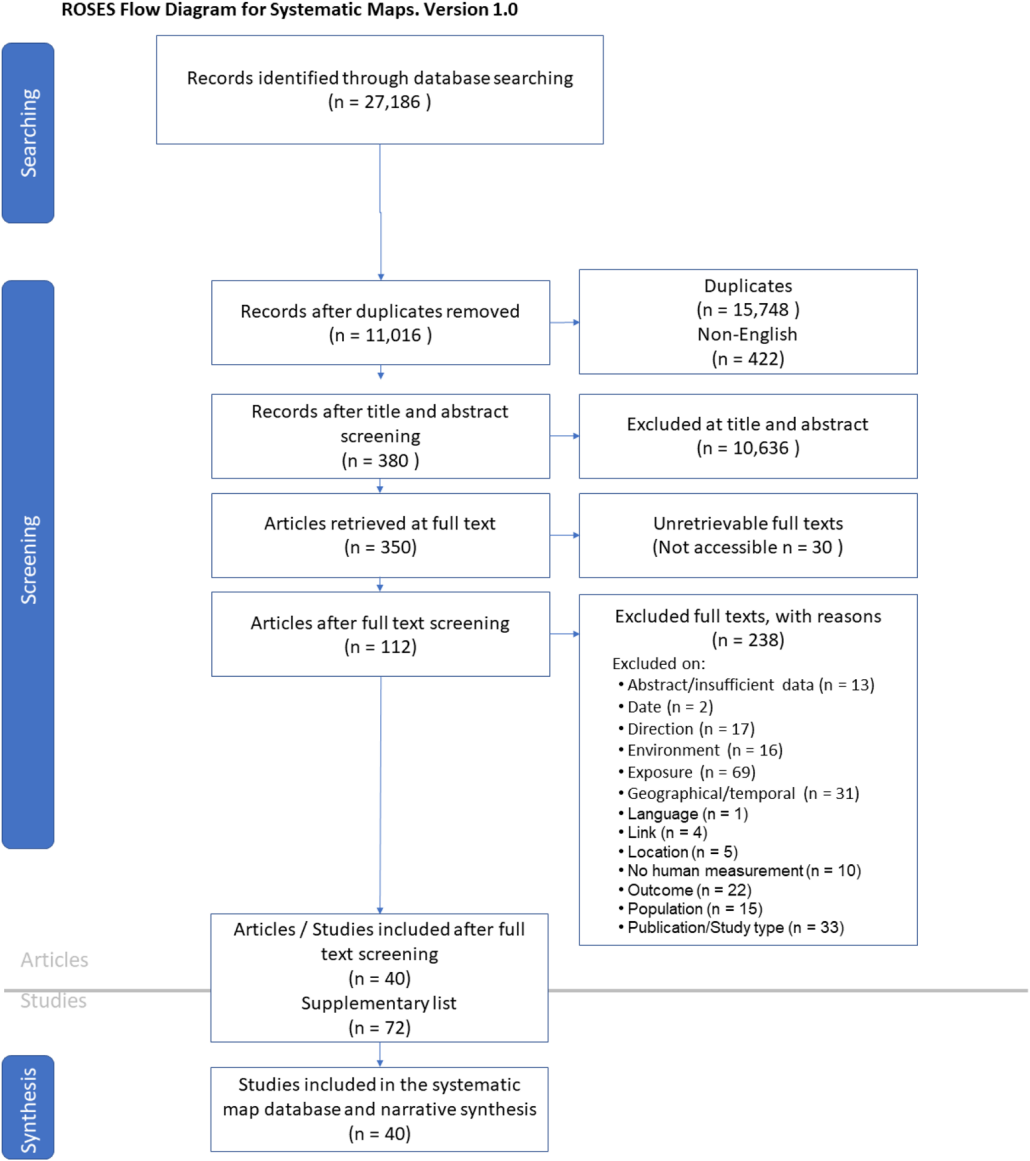
ROSES flow diagram from systematic maps for Map 1 [61]

For Map 1, the search yielded 27,186 hits which equated to 11,016 unique titles and abstracts for screening. 380 of these were considered eligible for full text screening of which 350 full texts were able to be retrieved. 40 were eligible for inclusion in the map (Fig. 2).

A total of 238 articles were excluded at full text (Additional file 6) and a further 72 articles highlighted in Additional file 2 for their relevance to the search question (although they do not fit the inclusion criteria—see details below). The full Map 1 database for all included articles, with all metadata, can be found in Additional file 7.

#### Geographical map

**Fig. 3:**
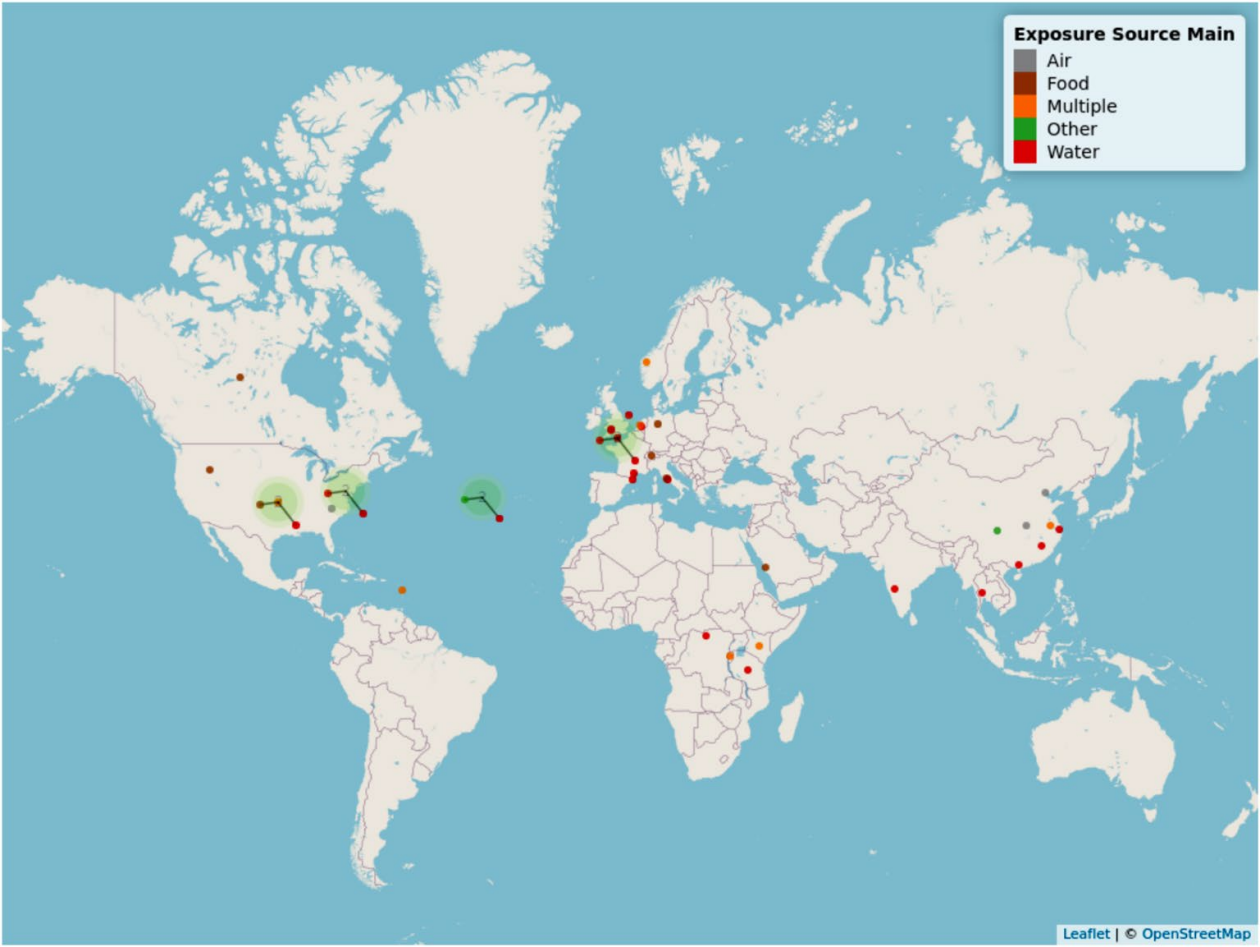
Geographical distribution of studies identified in Map 1. The different colours of the points indicate the different exposure source(s) which can be identified in the key. Points attached to lines indicate where multiple studies have been clustered together as a result of geographical proximity. Where the lines of multiple points join is a better indication of study location than the point location in this instance. An interactive map (where points can be clicked on and details of each study and a hyperlink to the publication) can be found as an html file in Additional file 8

The geographical location of included studies can be viewed in Fig. 3. To view an interactive version of this map, please download the html file provided as an additional file (“Map 1 geographical interactive—Additional file 8”).

Articles were not evenly distributed around the globe, with clusters found in Europe (n = 15), South East Asia (n = 8) and North America (n = 9). There were also a small number of studies undertaken in Africa. There were no studies investigating direct transmission of AMR from the environment to humans in South America and Oceania. In general, research study sites tended to be located where convenient and close to researchers’ location (based on author affiliation information). Therefore, the uneven geographical distribution of study sites is likely caused by where researchers undertaking this type of research are based.

A number of studies specified sampling in multiple countries in the United Kingdom. To avoid confusion these studies have been placed at the coordinates (latitude and longitude): 50.15852, − 1.258472. Studies with no information on where the sample sites were, were placed at the coordinates 38.49942, − 38.8872.

#### Publications over time

**Fig. 4:**
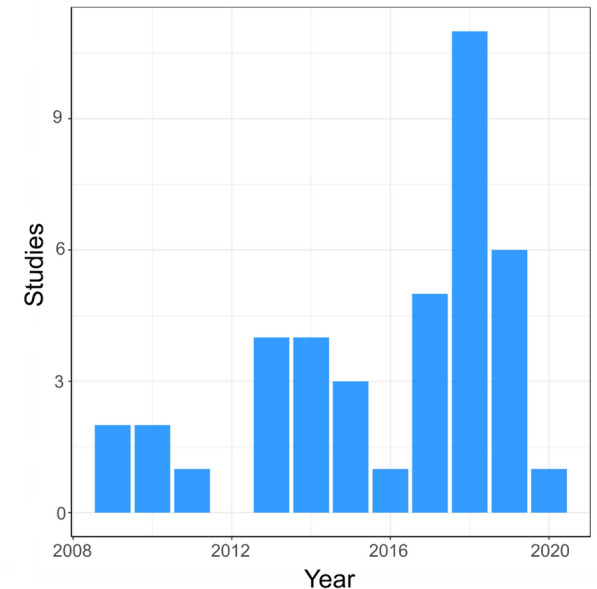
Map 1—year of publication. Bar chart showing the number of studies investigating transmission of antibiotic resistance from the environment to humans over time (between 2009 to present)

Figure 4 shows a trend of increasing number of publications over time with fluctuations throughout the time period, with a peak of 11 in 2018. The lack of a clear increase of studies over time is likely a reflection of this being an under researched topic area.

#### Study type

**Fig. 5:**
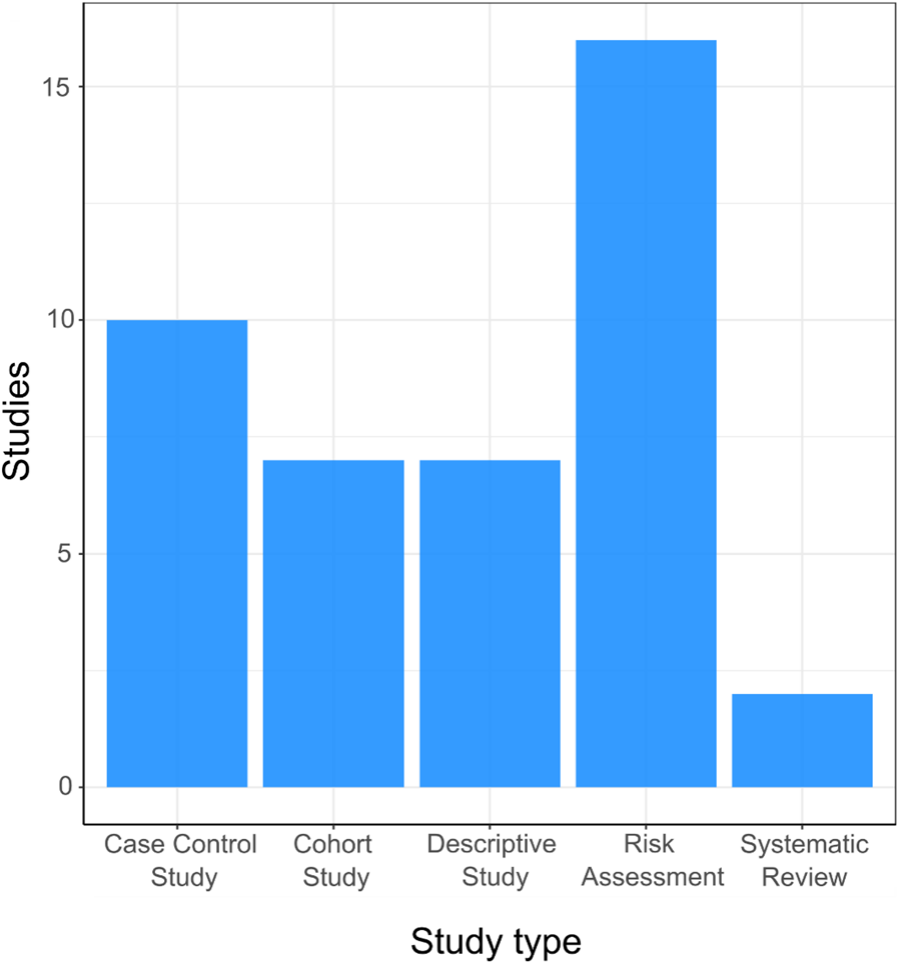
Map 1—study type. Bar chart showing the number of studies using different methodologies to investigate the transmission of antibiotic resistance from the environment to humans

The number of different study types associated with the 40 included publications can be seen in Fig. 5. The most commonly used study type were risk assessments (resulting in an estimated or measured exposure risk outcome) (n = 16), with systematic reviews being the least commonly used study type (n = 2).

#### Exposure route

**Fig. 6:**
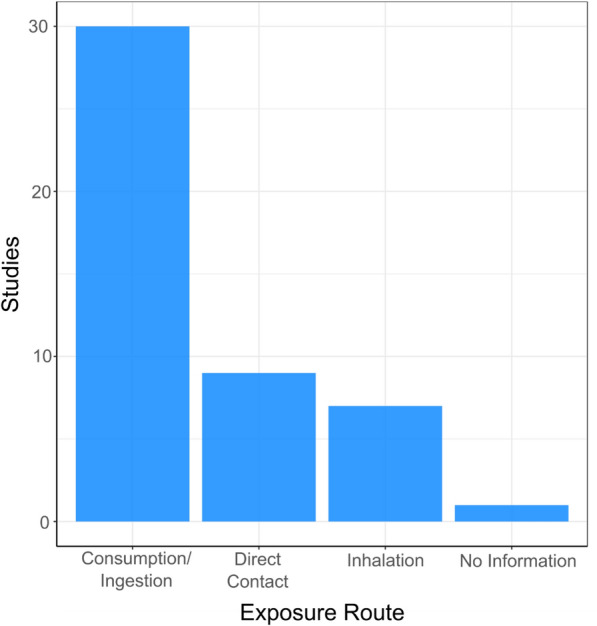
Map 1—exposure routes. Bar chart displaying the number of studies exploring different routes of human exposure to environmental antibiotic resistance

The bar graph in Fig. 6 shows the number of studies exploring different exposure routes. Most studies reported a specific exposure route. However, for nine exposure routes (n = 7 from studies with one exposure route investigated and n = 2 from a study investigating two exposure routes), the exposure route was not explicit but could be inferred from other information in the paper. For one study, it was not possible to infer the exposure route from the information provided which has resulted in “No information” on the base graph [62]. Information on which studies the exposure route was inferred can be found in the systematic map database for Map 1 (see Additional file 7). Consumption/ingestion was the most common (n = 30), followed by direct contact (n = 9) and inhalation (n = 7). Of the 40 studies included in the map, 7 of these studies investigated two exposure routes, with remainder (n = 33) exploring only one type of exposure route.

#### Outcome

**Fig. 7:**
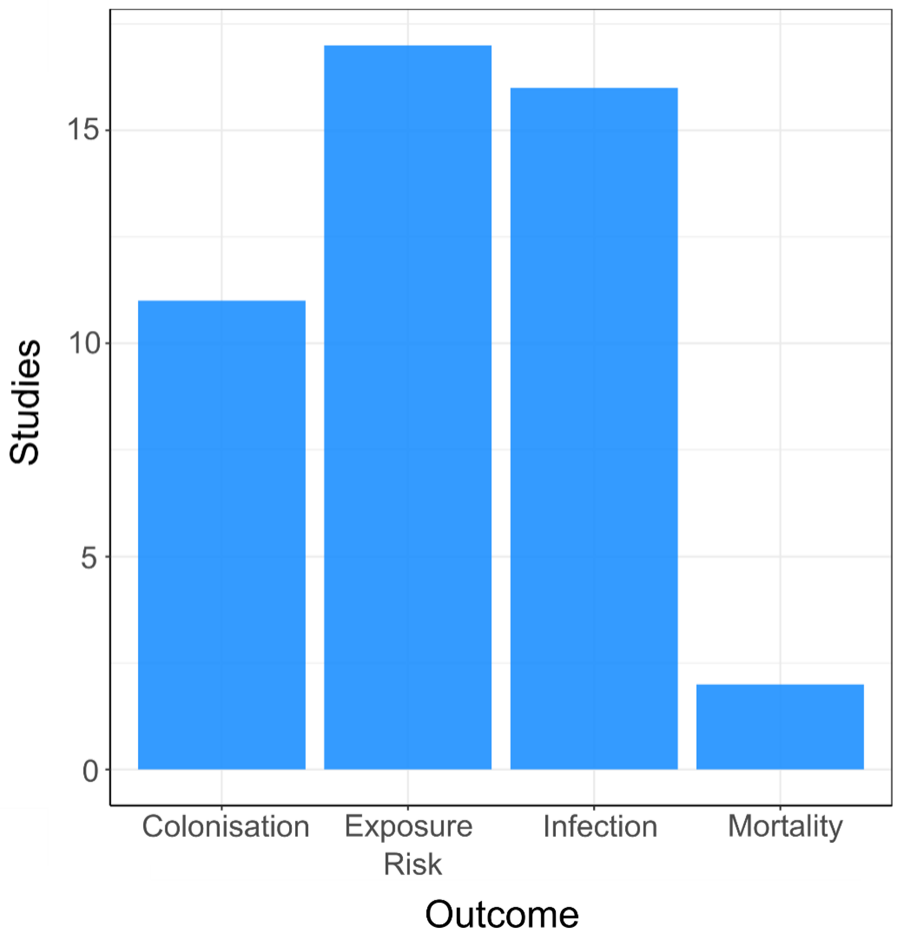
Map 1—human health outcomes. Bar chart showing the number of different human health outcomes investigated in the included studies. Exposure risk = Measured or estimated exposure risk

Figure 7 shows the number of different outcomes reported in the included studies. Colonisation (n = 11), estimated/measured exposure risk (n = 17) and infection (n = 16) are reported a similar number of times with mortality being reported significantly fewer times (2 studies). Out of the 40 studies identified, 6 of these reported two different outcomes with 34 measuring only one outcome.

#### Species studied

**Fig. 8:**
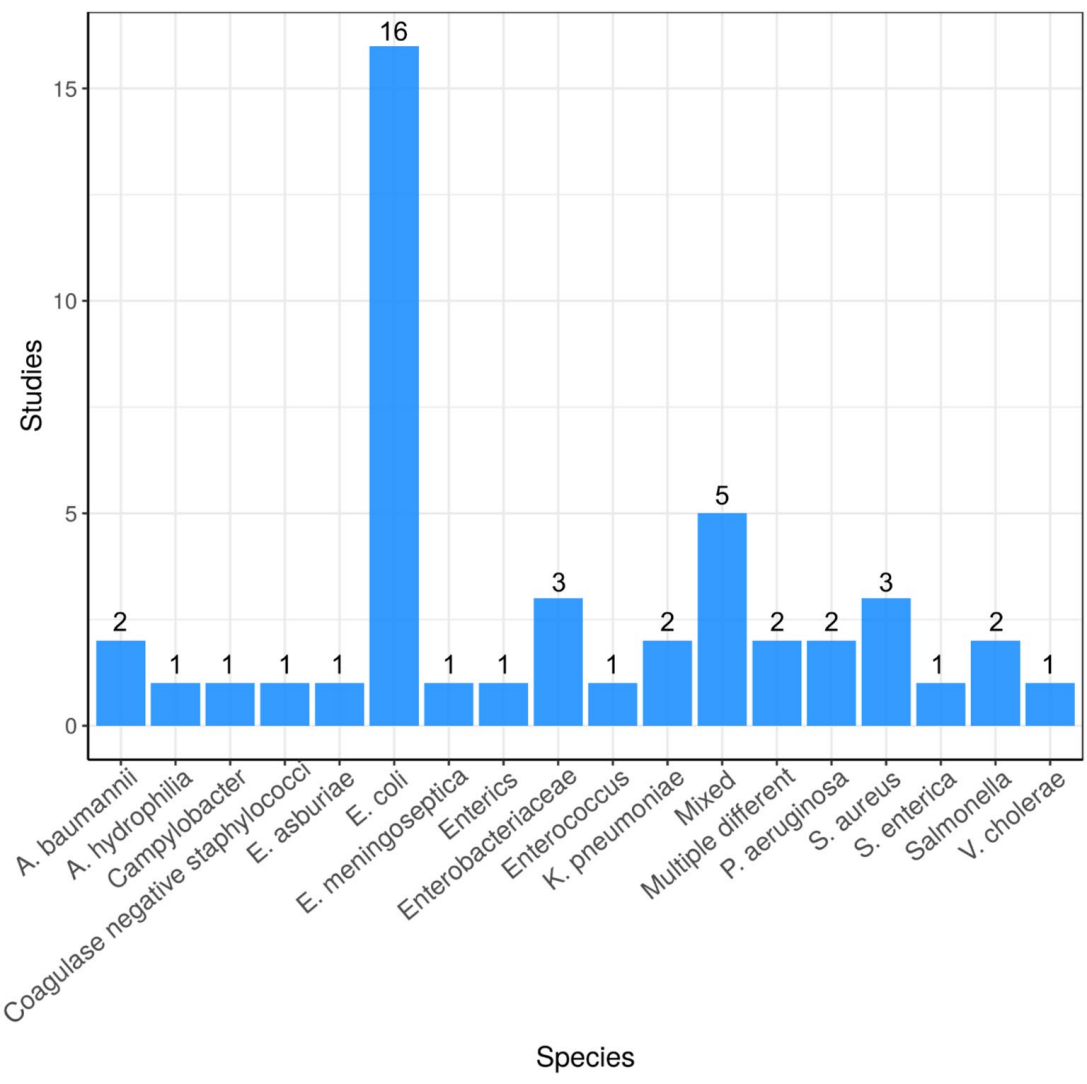
Bar chart showing number of different bacterial species studied. “Mixed” = when experimental designed took a whole community approach (e.g. metagenome sequencing)

The different bacterial species investigated in the included studies are reported in Fig. 8. *E. coli* is the most highly studied species having been reported in 16 publications with the majority of other species studied only investigated by one publication.

#### Exposure (main categories) by health outcome

**Fig. 9:**
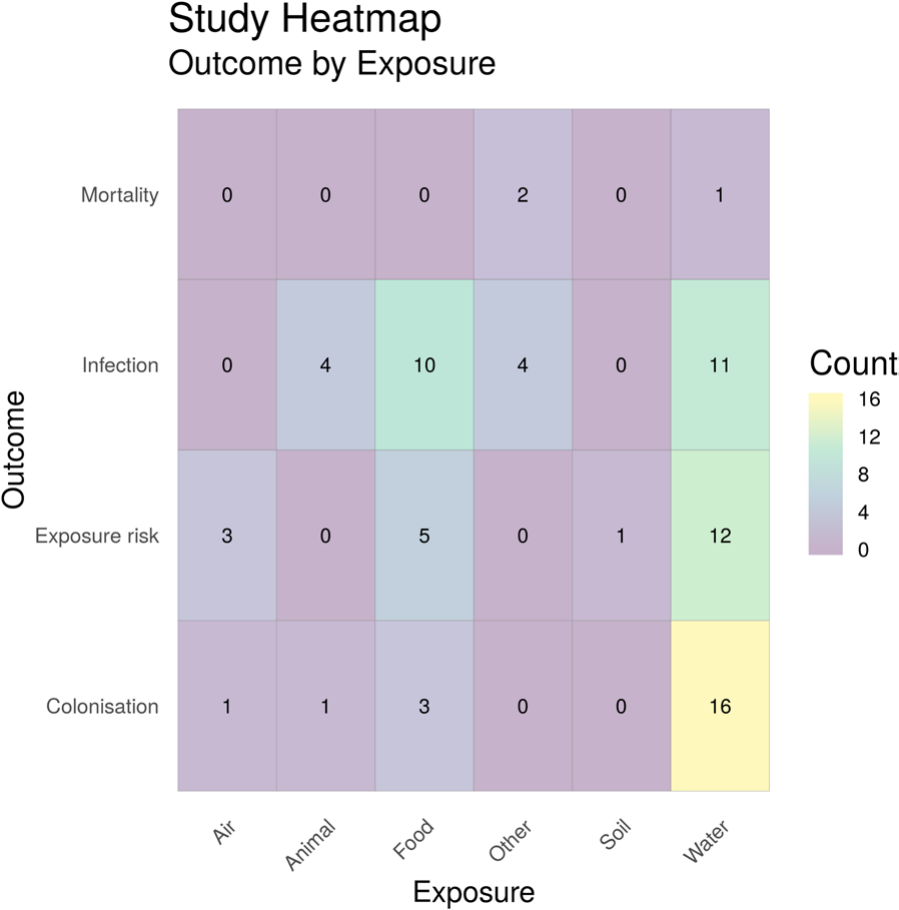
Heatmap showing the main categories of environmental exposure sources by human health outcome. “Exposure risk” = estimated or measured exposure risk, i.e. where articles have determined the number of ARG/ARGs in a certain volume of the environmental matrices and determines the number of ARB/ARGs humans are exposed to based on volume consumed/inhaled. “Animal” = Amphibians, animal bite, reptiles, wild birds and wild animals. “Food” = wild meat and plants consumed raw. “Other” = Accident (fall, road traffic accident), earthquake

Figure 9 shows a heatmap of the main categories of environmental exposure sources by human health outcome. Water environments were the most researched natural environment, followed by eligible food sources. For water environments, colonisation (n = 16) was the most frequently researched outcome, followed by estimated/measured exposure risk (n = 12) and infection (n = 11) and only one study investigated exposure to water and mortality. In contrast, the most frequently studied outcome for exposure via food was infection (n = 10), whereas colonisation from food was only reported in three studies. Out of the 40 studies identified in Map 1, 6 of these reported two outcomes. In addition, 13 studies investigated two or more exposure sources per study.

**Fig. 10:**
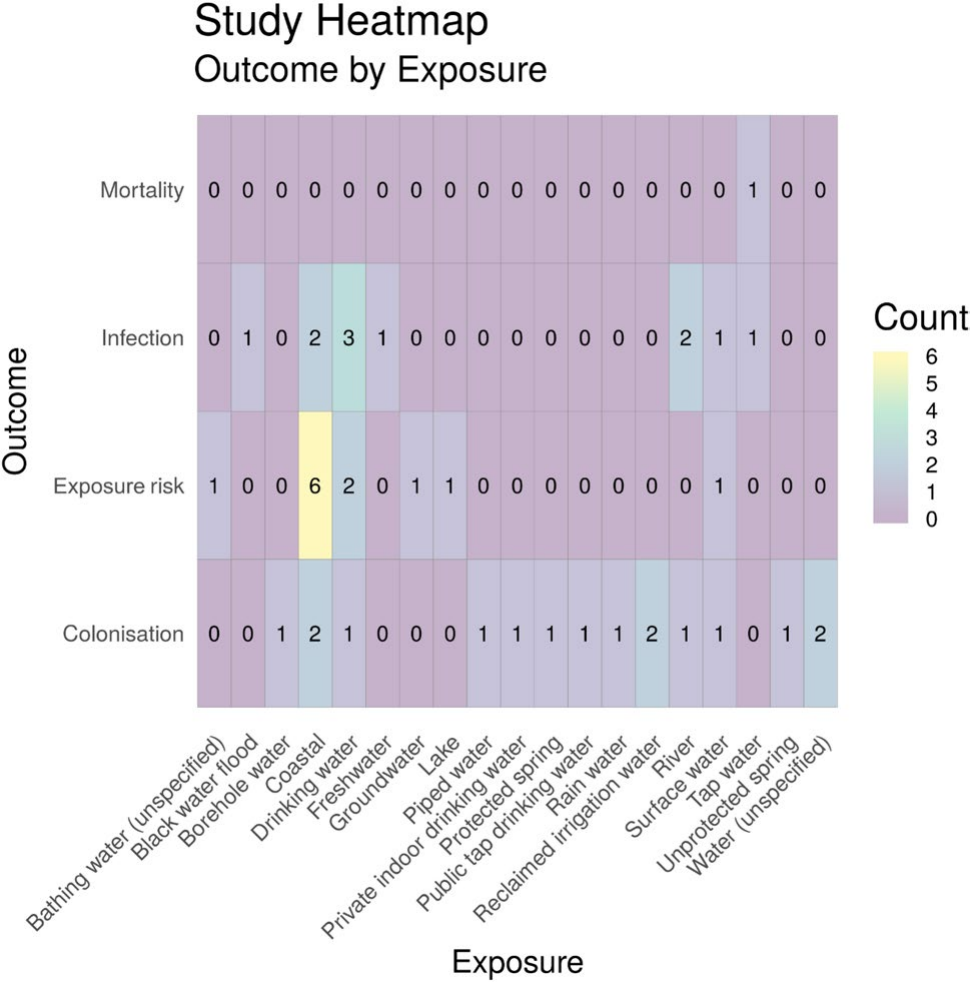
Heatmap showing the number of studies investigating health outcomes by exposure to water environment subcategories. “Exposure risk” = Estimated or measured exposure risk

**Fig. 11:**
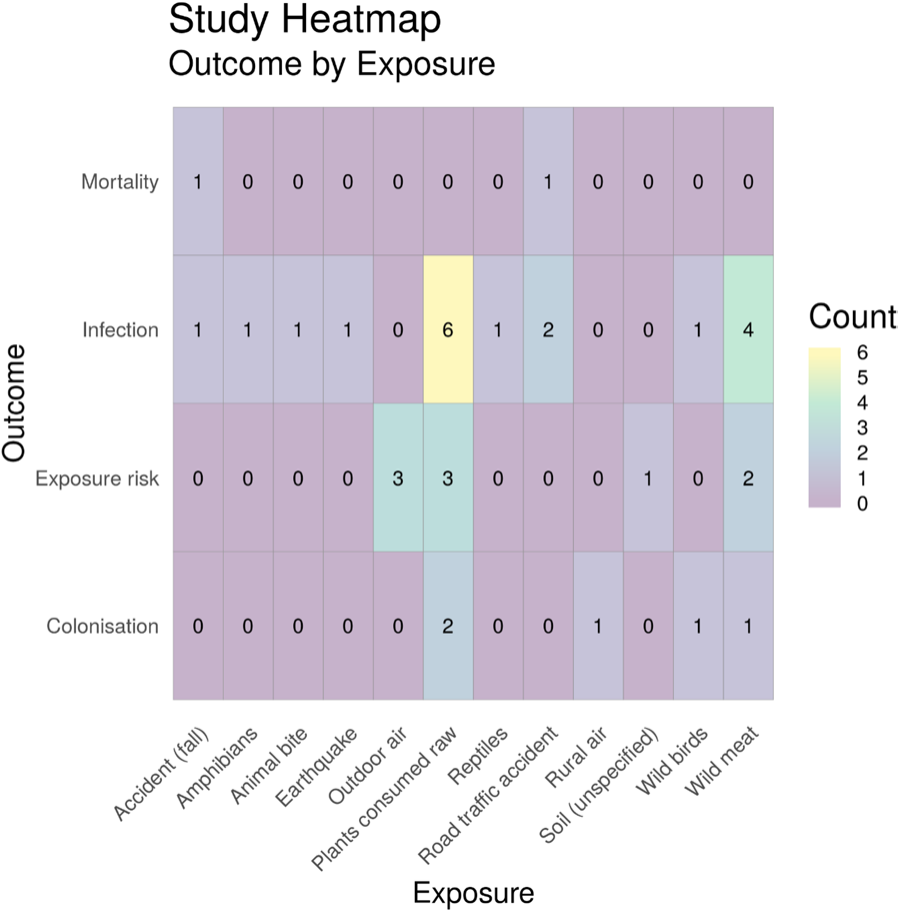
Heatmap showing the number of studies investigating health outcomes by exposure to ‘other’ environment subcategories. “Exposure risk” = estimated or measured exposure risk

To identify the corresponding publications that are presented in the heatmap, Fig. 9, below [and subsequent heatmaps for Map 1 (Figs. 10 and 11)], we have provided a column in the Additional file 7 database titled “Corresponding heatmap figure” (column AC). This column shows the figure number(s) in this study that each publication is displayed in. In addition, to further interrogate this data, columns D, F, H, J, L, N and P display the exposure type(s) in each publication and columns U and V display the outcome which align with the X and Y axis of Fig. 9, respectively. For Figs. 10 and 11, the exposure sub categories can be viewed in columns E, G, I, K, M, O and Q.

#### Exposure (sub-categories) by health outcome

Two further heatmaps were produced to investigate exposure sub-categories and capture the diversity of research into different water environments (Fig. 10), and ‘other’ categories (Fig. 11). There are 19 different eligible water sources reported in this map with the most commonly reported being coastal water (n = 10). The most frequently studied health outcome with exposure to coastal water was estimated or measured exposure risk (n = 6). Across all aquatic exposure environments the most frequently reported health outcome was colonisation (n = 16). Only one study was identified that investigated mortality due to antibiotic resistance and exposure to water (tap water) [63].

Figure 11 shows all other exposure source subcategories by outcome. The most studied exposure source here was plants consumed raw (n = 11), followed by exposure to wild meat (n = 7). Both of these showed infection as the most frequently reported outcome. Considering all of the exposure sources, infection was the most commonly reported outcome overall (n = 18). Again, very few studies (n = 2) investigated mortality associated with exposure to antibiotic resistance in these environmental sub-categories.

### Map 2: What research evidence is there measuring the prevalence of ARB in the environment in the UK?

#### Description of review process

**Fig. 12:**
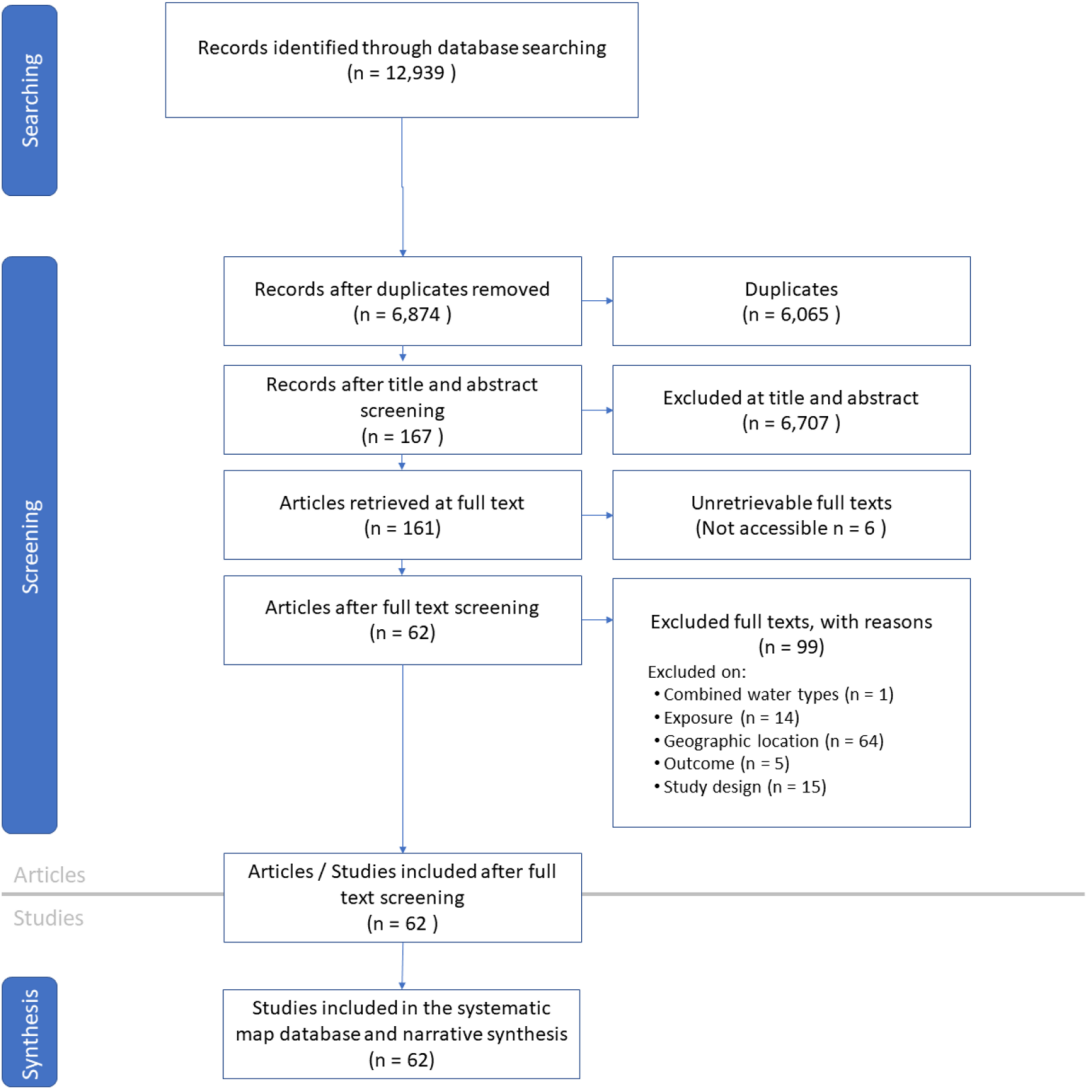
ROSES flow diagram for systematic maps for Map 2 [61]

For Map 2, the search yield 12,939 hits and 6874 unique titles and abstract for screening once duplicates have been removed. 167 of these studies were selected for full text screening, and 62 were included in the map (Fig. 12). The full Map 2 database, with extracted metadata, can be found in Additional file 7.

#### Geographical map

**Fig. 13:**
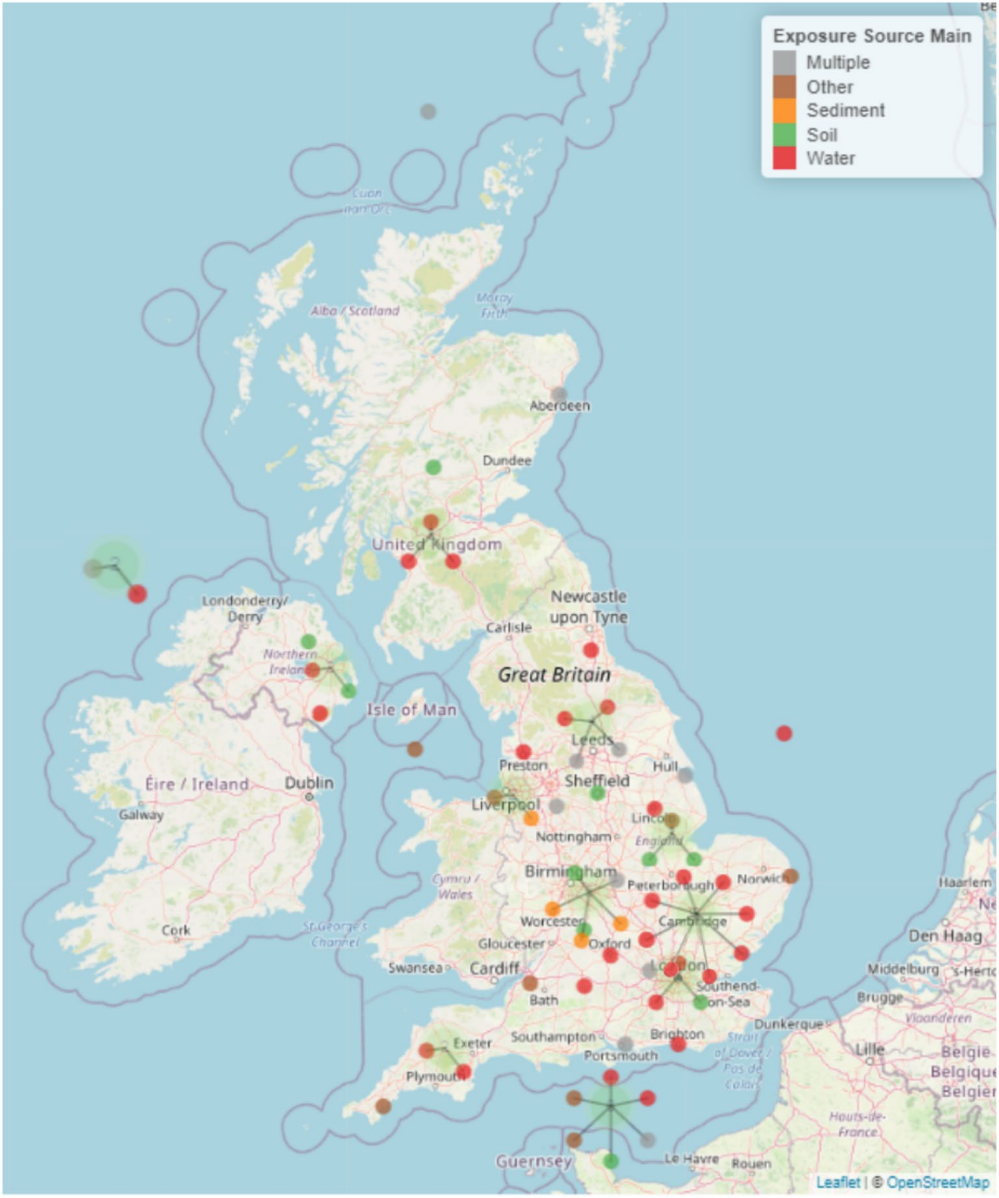
Geographical distribution of studies identified in Map 2. The different colours of the points indicate the different exposure source(s) which can be identified in the key. Points attached to lines indicate where multiple studies have been clustered together as a result of geographical proximity. Where the lines of multiple points join is a better indication of study location than the point location in this instance. An interactive map (where points can be clicked on and details of each study and a hyperlink to the publication) can be found as an html file in Additional file 9

The sampling locations of the 62 included articles can be seen in Fig. 13. To view an interactive version of this map, please download the html file provided as an additional file (“Map 2 geographical interactive—Additional file 9”).

In contrast to Map 1, study sites were more evenly distributed around the UK for Map 2. However, there is still a trend that sampling is undertaken relatively close to where researchers are based according to author affiliation information.

For studies that do not specify the sampling site and instead use terms such as UK, England, Wales, Scotland or N. Ireland we have chosen a set of nominal longitude/latitude points to represent these studies. Nominal coordinates were placed in the sea to avoid confusion with studies with specified sampling sites. The nominal latitudes and longitudes were:

- England: 53.96486, 1.615305.
- N. Ireland: 55.51571, − 9.35855.
- Scotland: 59.61634, − 4.27592.
- Wales: N/A (no studies specified just Welsh study sites without specifying the exact site).

United Kingdom/Multiple countries sampled (e.g. sample sites in England and Wales): 50.15852, − 1.25847.

#### Publications over time

**Fig. 14:**
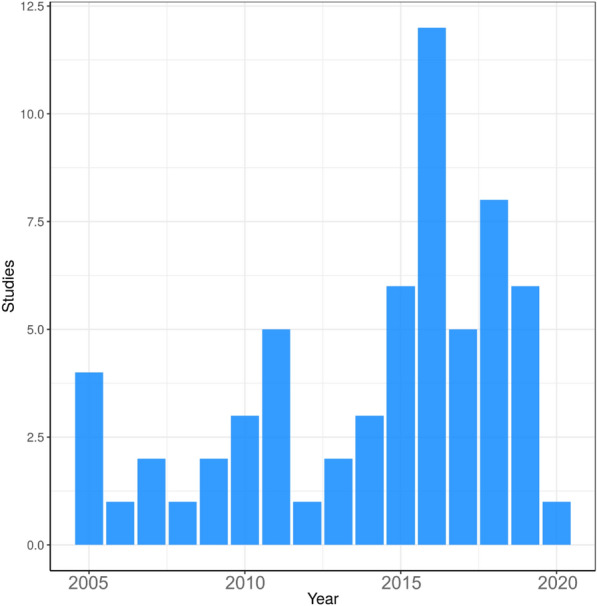
Map 2—date of publication. Bar chart showing the number of studies reporting the abundance of antibiotic resistance in the environment over time (between 2005 to present)

Like Map 1, there is a weak increasing trend of published articles over time for Map 2 (Fig. 14). The greatest number of articles were published in 2016 (12 studies).

#### Methodology

**Fig. 15:**
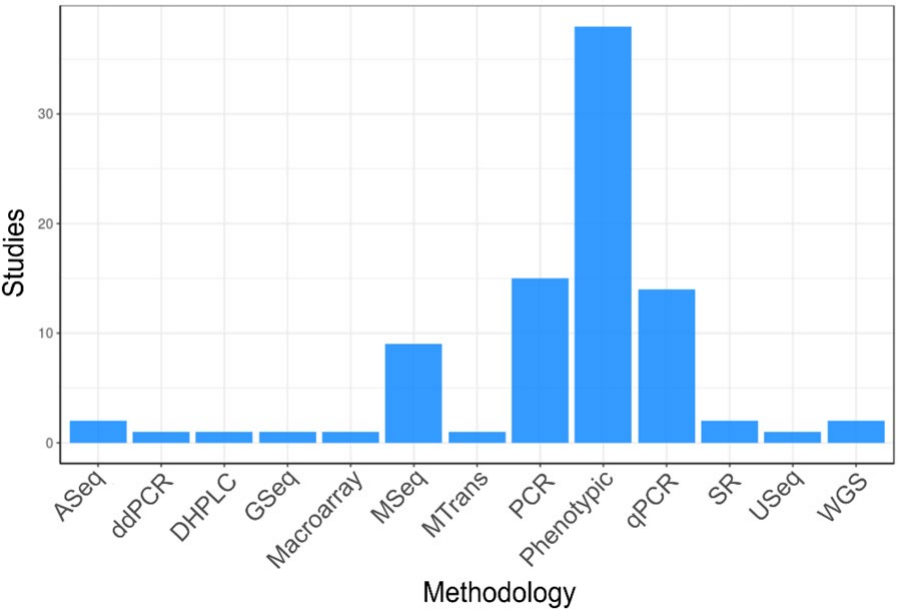
Map 2—methodologies. Bar graph showing the number of studies reporting using different methods to measure antibiotic resistance in the natural environment. *ASeq* amplicon sequencing, *ddPCR* digital droplet polymerase chain reaction, *DHPLC* denaturing high performance liquid chromatography, *GSeq* genomic sequencing, *MSeq* metagenomic sequencing, *MTrans* metatranscriptomics, *PCR* polymerase chain reaction, *qPCR* quantitative polymerase chain reaction, *SR* systematic review, *USeq* unspecified sequencing, *WGS* whole genome sequencing

Figure 15 shows the different methodologies used when researching antibiotic resistance in the environment. Phenotypic methods (such as disk diffusion assays and plating data) are the most commonly used methods with 37 studies reporting using this method. The other three most popular methodologies are molecular methods: polymerase chain reaction (PCR) (15 studies); quantitative PCR (qPCR) (14 studies) and metagenomic sequencing (9 studies). All other methodologies used are only found in a small number of studies and include other (non-metagenomic) sequencing technologies and systematic reviews.

#### Bacterial species

**Fig. 16:**
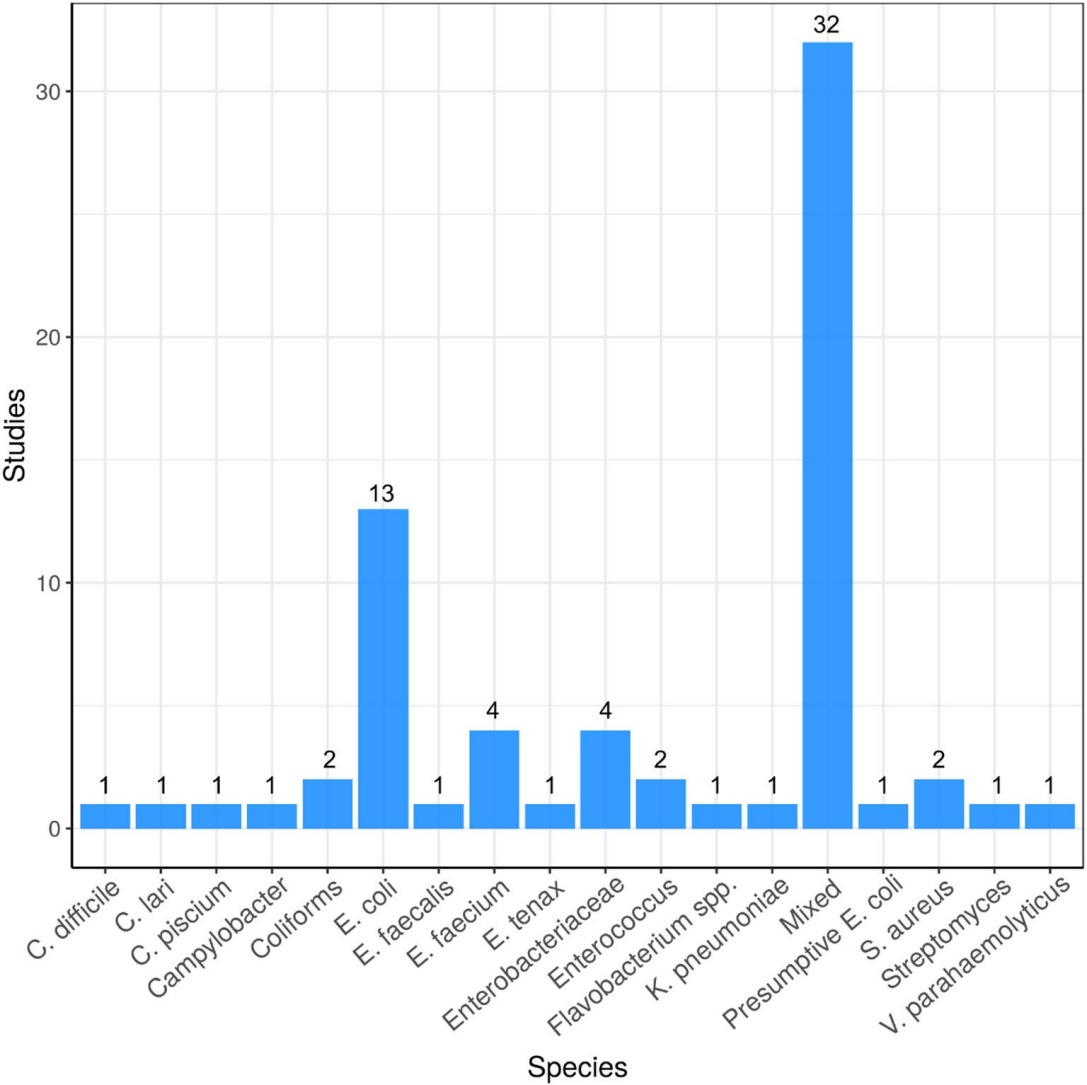
Map 2—bacterial species. Bar chart showing the number of studies that investigate antibiotic resistance in particular bacterial species sampled from the environment. “Mixed” = when experimental designed took a whole community approach (e.g. metagenome sequencing)

Data were extracted from included studies on environmental bacteria species that were resistant to antibiotics. Most studies investigated mixed communities of bacteria (n = 32) (Fig. 16). Bacterial species commonly found in faeces, and/or transmitted by the faecal–oral route are also common in these studies (*E. coli*, Enterobacteriaceae, coliforms, *E. faecalis*, *E. faecium*, *Enterococcus* spp., *Campylobacter* spp. (n = 27)).

#### Exposure (main categories) vs outcome

**Fig. 17:**
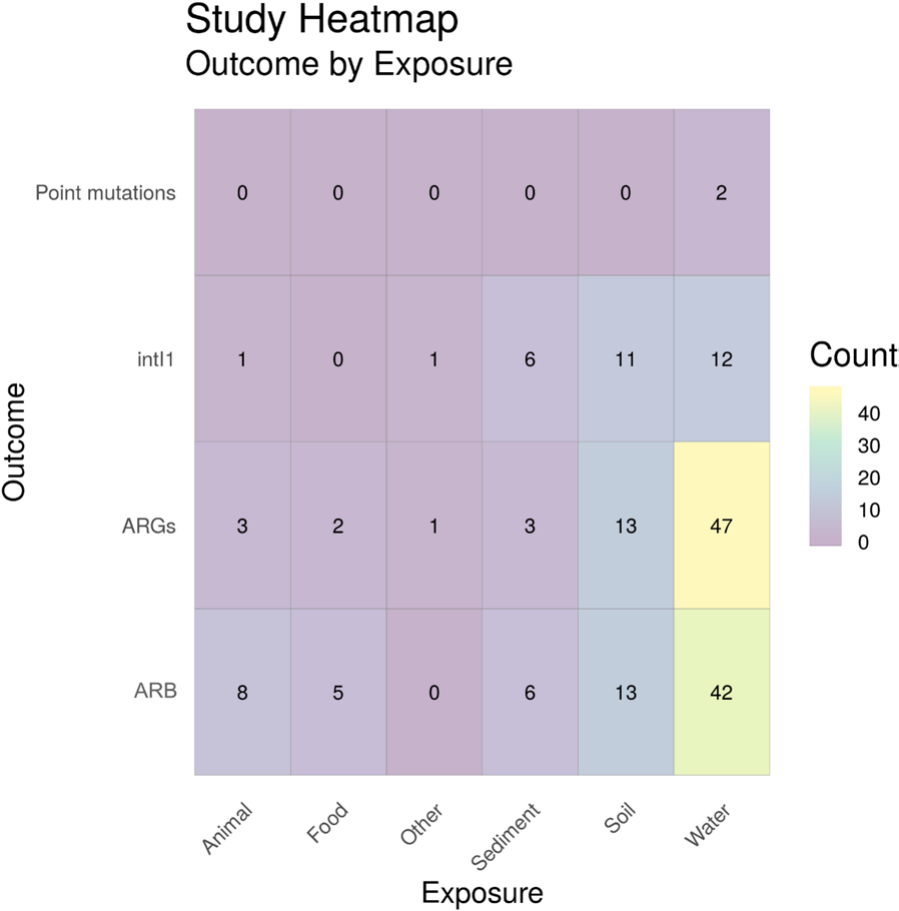
Heatmap showing main exposure sources vs outcome. ARGs = Antibiotic resistance genes, ARB = antibiotic resistance bacteria (e.g. phenotypic resistance) and intI1 = study either measure the gene intI1 or the class 1 integron/integrase. “Other” = animal faeces, food (wild meat or plants consumed raw), plankton and animal lesion

Figure 17 is a heatmap showing the main exposure categories by outcome. As can be seen in the heatmap, water is the natural environment that is most studied of all natural environments in the UK with ARGs being the most reported resistance metric for water environments. Conversely, when looking at all environmental compartments, AMR bacteria are quantified the most frequently (in all Map 2 heatmaps and the database, AMR refers to phenotypic resistance and ARGs refers to where molecular methods were used to identify specific genes). Of the 62 full text articles studies 26 investigated more than one outcome and 26 studies investigated more than one exposure.

**Fig. 18:**
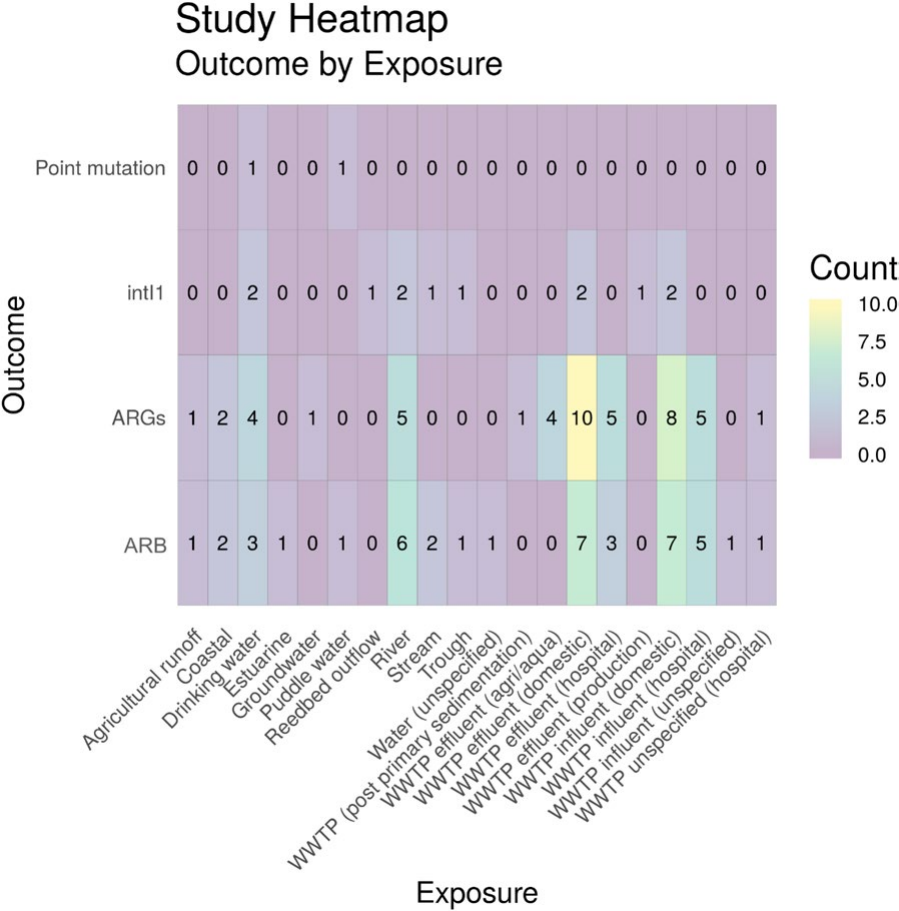
Heatmap showing water exposure sub categories vs outcome. ARGs = Antibiotic resistance genes, ARB = antibiotic resistance bacteria (e.g. phenotypic resistance) and intI1 = study either measure the gene intI1 or the class 1 integron/integrase. WWTP = wastewater treatment plant

**Fig. 19:**
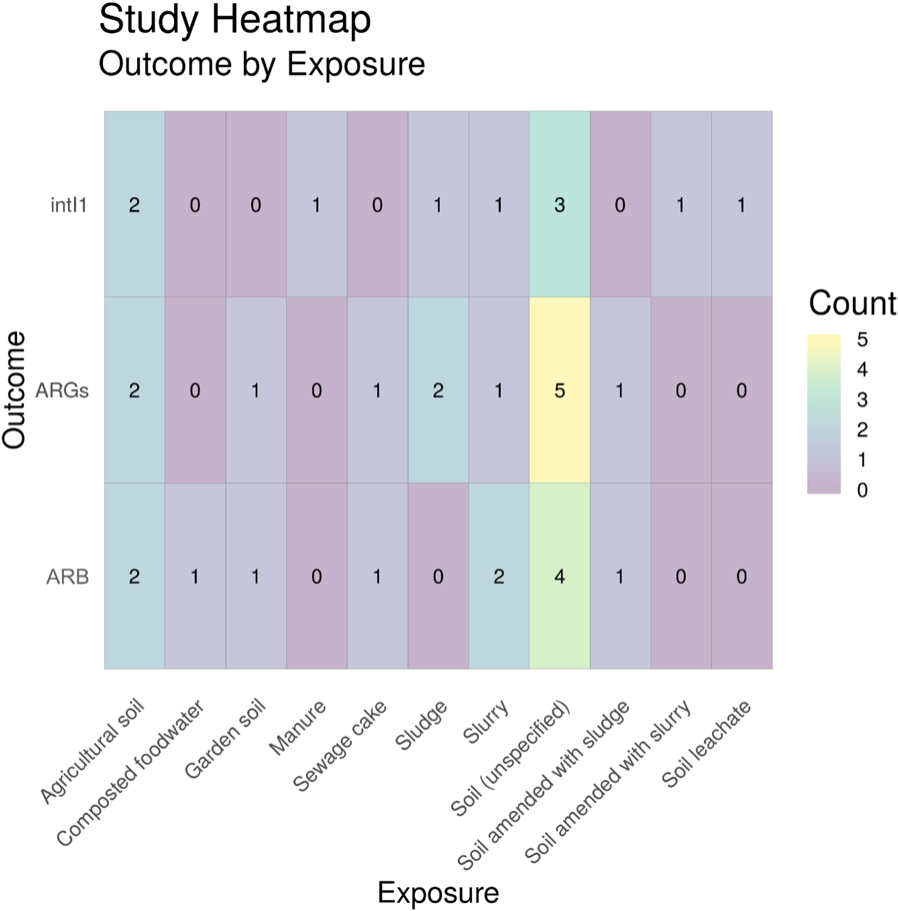
Heatmap showing soil exposure sub categories vs outcome. ARGs = Antibiotic resistance genes, ARB = antibiotic resistance bacteria (e.g. phenotypic resistance) and intI1 = study either measure the gene intI1 or the class 1 integron/integrase

**Fig. 20:**
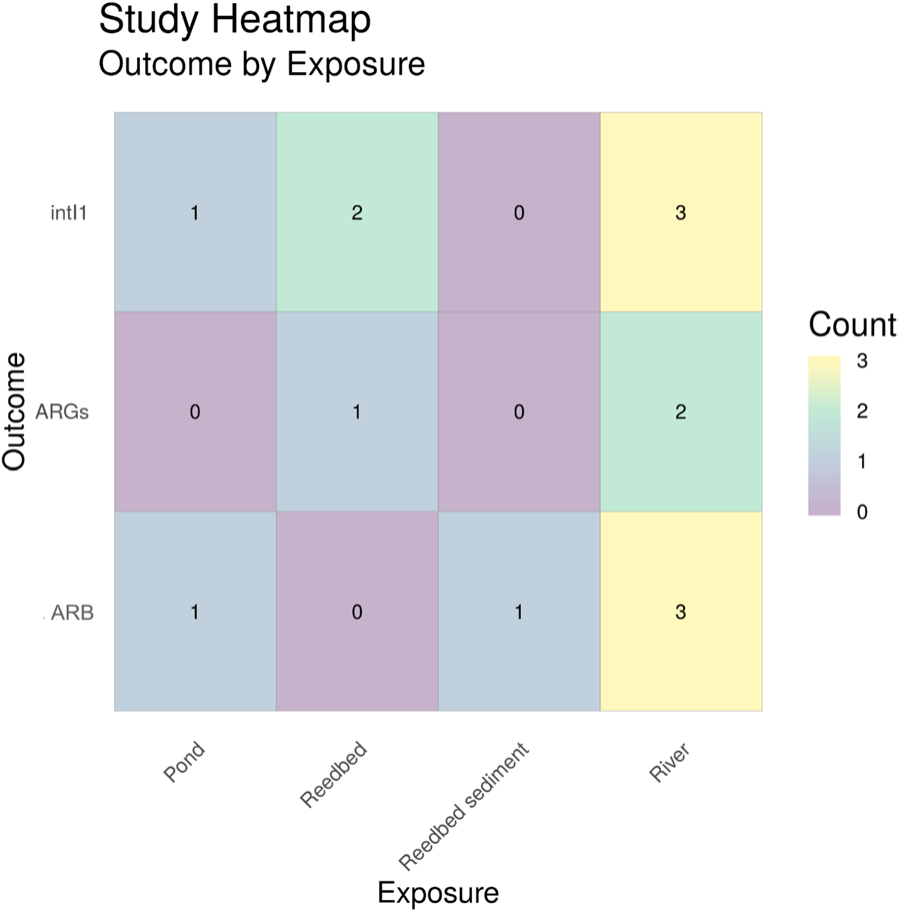
Heatmap showing sediment exposure sub categories vs outcome. ARGs = Antibiotic resistance genes, ARB = antibiotic resistance bacteria (e.g. phenotypic resistance) and intI1 = study either measure the gene intI1 or the class 1 integron/integrase

**Fig. 21:**
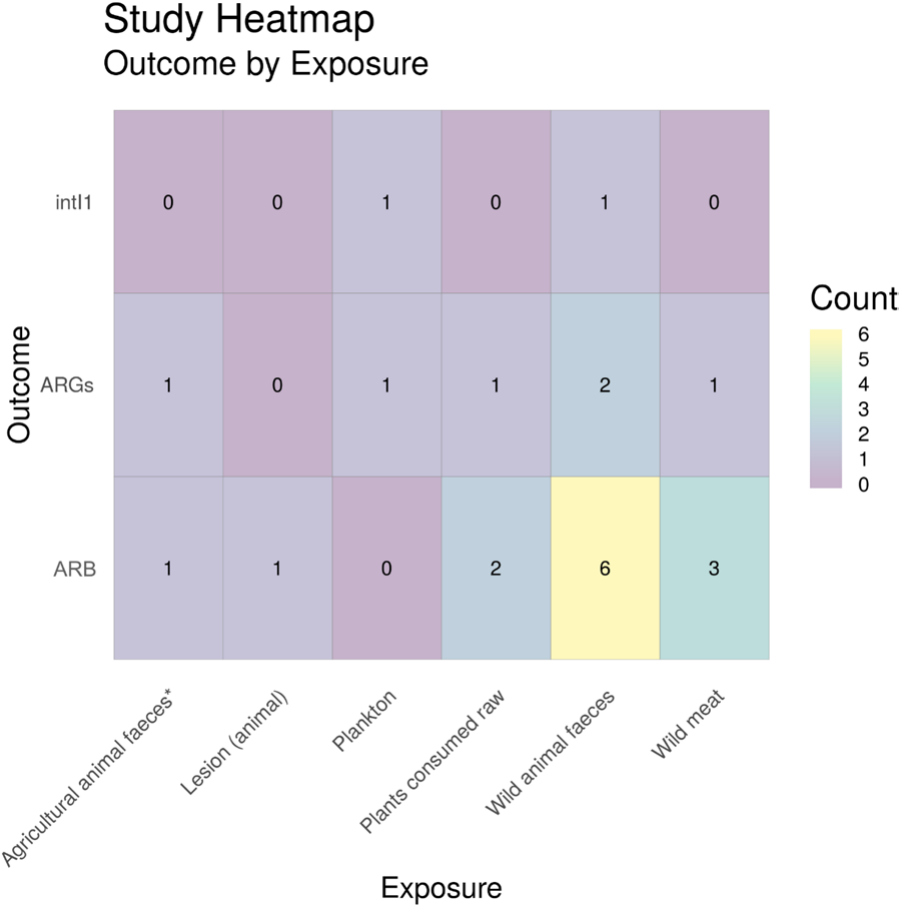
Heatmap showing ‘other’ exposure sub categories vs outcome. ARGs = Antibiotic resistance genes, ARB = antibiotic resistance bacteria (e.g. phenotypic resistance) and intI1 = study either measure the gene intI1 or the class 1 integron/integrase. * = Agricultural animals that have not been treated with antibiotics

To identify the corresponding publications that are presented in the heatmap below [and subsequent heatmaps for Map 2 (Figs. 18, 19, 20 and 21)], we have provided a column in the Additional file 7 database titled “Corresponding heatmap figure” (column Y). This column shows the figure number(s) in this study that each publication is displayed in. In addition, to further interrogate this data, columns D, F, H, J and L display the exposure type(s) in each publication and columns N and O display the outcome which align with the X and Y axis of Fig. 17, respectively. For Figs. 18, 19, 20 and 21, the exposure sub categories can be viewed in columns E, G, I, K and M.

#### Exposure (sub-categories) vs outcome

##### Water

As seen in Fig. 17, water was the most highly studied environmental setting in terms of total numbers of articles. However, as can be seen in Fig. 18, it is also an extremely diverse research area with many different types of water environments being studied. This is particularly evident when looking at the other main exposure sources in the following sections (see Figs. 19, 20 and 21 below for soil, sediment and other, respectively). Even within just wastewater treatment plants, there are many different types of wastewater being researched. This is related to both influent and effluent and different sources of wastewater (e.g. domestic, hospital, production and agriculture/aquaculture). The most commonly studied outcome in a water environment was ARGs in domestic wastewater treatment plant effluent (n = 10). Of the 62 full text articles, 18 investigated multiple water exposures and 15 investigated more than one outcome.

##### Soil

As can be seen in Fig. 19, unspecified soil is the most common environmental substrate researched, with 12 different soil environments being tested for the relevant resistance outcomes, followed by agricultural soil.

##### Sediment

At total of 14 sediment sites were tested for either *intI1*/class 1 integrons, ARGs or ARB with river sediment being the most commonly studied sediment environment (8 articles) and reedbed sediment being the least studied (1 article) (Fig. 20).

##### Other

Finally, Fig. 21 shows a heatmap of remaining relevant exposure sources (classed as “other”). This heatmap shows a variety of different exposure sources including different types of food (both wild meat and plants consumed raw), wild animal sources (both faeces and lesions on the animal), agricultural animal faeces (which normally wouldn’t be included as a relevant exposure source as agricultural animals are often treated with antibiotics, however the study included here states that no antibiotics were used in the rearing of these animals [64]) and plankton. Wild animal faeces was by far the most studied environmental matrix (n = 9), followed by wild meat food sources (n = 4).

#### Knowledge gaps and clusters

Generally, for both maps most work has investigated water environments but within this there is a diverse range of sample types. For Map 1, research on water environments (e.g. coastal or drinking water) has focused on those with which humans interact so may pose a greater risk of transmission. Conversely, in Map 2 there is a focus towards sample sites and types of sample matrices with a high density of bacteria, such as wastewater treatment plant influent and effluent. This could be as a result of bias of sampling by researchers towards samples that are likely to have a high density of bacteria which could increase the likelihood of ARB/ARG detection.

Air environments are under researched in both maps. Presumably, even though a large number of the population is in daily contact with outdoor air, the density of bacteria in this environment is low and there may, therefore, be more methodological challenges in obtaining accurate data.

Whilst it could be argued that air environments are not worth investigating because the low abundance of ARB/ARGs, humans are in constant contact with air environments. Whilst concentrations may be low, over a lifetime exposure to ARB/ARGs in air could be high through constant exposure. It is, therefore, deemed important to overcome methodological challenges fill knowledge gaps such as these.

Both maps also illustrate clustered sampling sites, presumably close to where the researchers are based. This has resulted in knowledge gaps for environments in certain countries (Map 1) and certain regions of the UK (Map 2).

##### Map 1

There is a clear lack of global empirical evidence for the transmission of AMR from the natural environment to humans with only 40 relevant articles being collated for Map 1 with a lack of data coming from certain areas of the globe. However, there were 72 supplementary articles that compares ARB/ARGs in both clinical samples and environmental samples but do not investigate an exposure route which shows a growing interest in this research area. More effort must occur, therefore, to establish transmission routes from environmental to clinical setting.

As previously stated, water was the most highly studied environment (n = 40) with food (excluding animals/fish that are reared on high levels of antibiotics and crops that are always consumed cooked) being the next most studied matrix (n = 18). For estimated exposure risk assessments, these environments are easier to quantify than say exposure and transmission from soil and wild animals as there are defined quantifiable volumes of water and food ingested or consumed. For example, Leonard et al. 2015 identified and collated a number of publications reporting volume of water ingested during water sport sessions. Using densities of third generation cephalosporin resistant *E. coli* in bathing waters, exposure risk was calculated based on volume of water ingested and concentration of resistant *E. coli* [65]. Similarly, O’Flaherty et al. 2019 quantified the number of antibiotic resistance *E. coli* found on lettuce and created a model to estimate exposure risk to humans based on the consumption of a nominal amount of lettuce [66].

When undertaking other types of research other than estimated exposure risk studies (such as cohort studies), food (n = 12) and water (n = 28) exposure sources are also easier to quantify contact with and have a comparator group, compared to air (n = 1), animal (n = 3) and soil (n = 0) where two groups of “exposed” and “not exposed” are harder to define. Similarly, from all study types, ingestion/consumption (n = 30) is the most studied exposure route whereas direct contact (n = 9) and inhalation (n = 7) are studied less frequently as a result of being able to easily identify a comparator group.

In regards to outcomes, “estimated exposure risk,” “colonisation” and “infection” are reported to a similar degree, whereas there are significantly fewer studies investigating “mortality,” presumably because it is difficult to trace these human health outcomes back to the natural environment. One of the two articles reporting this were as a result of two types of accidents resulting in environmental exposure [67, 68]. Because infections do not necessarily occur at the time of exposure, epidemiological tracing of outbreaks is extremely challenging as individuals may be colonised for weeks, months or years before infection. In addition, infections may occur via transmission from exposed individuals to more vulnerable people.

Finally, the majority of research articles investigate *E. coli* which are used as a faecal indicator species that indicate if the environment has been impacted by anthropogenic pollution [69]. Although a number of strains of *E. coli* are human gut commensals, others are important opportunistic pathogens [70]. Commensal *E. coli* not associated with infections may colonise the gut with no adverse human health outcomes but may be able to transfer ARGs it harbours, via horizontal gene transfer, to pathogenic organisms in the gut making the infection more difficult to treat. Molecular epidemiology approaches focusing on ARG or mobile genetic elements may help in attributing an environmental origin of AMR. However, this still poses many challenges due to the complexity of gene transfer events within microbial populations over time.

##### Map 2

There are more studies included in Map 2 (UK based) than in Map 1 (global database). This shows that the quantification of ARB, ARGs, *intI1* and point mutations is significantly better researched than empirical evidence of transmission from the environment to humans. Despite the topic area being better researched, there is still a bias towards quantifying AMR in water environments and more specifically different types of wastewater environments.

In terms of what is being measured, ARGs (n = 78) and ARB (n = 74) are both frequently studied in various natural environments, with *intI1* (n = 31) being targeted less often. This is unsurprising as, although qPCR targeting *intI1* has used as a proxy for environmental resistance [53–57], other methodologies (such as metagenomics) targeting multiple ARGs at once and phenotypic methodologies (such as plating and antibiotic susceptibility testing) are significantly cheaper to undertake. In addition, qPCR can also be used for specific ARGs. Point mutations are rarely characterised in the publications identified in this study (n = 1 study, n = 2 environments). Whilst point mutations are an important mechanism deployed by bacteria to resist antibiotics, they are not transferable as in the case of ARGs associated with mobile genetic elements and cannot, therefore, undergo horizontal gene transfer. Although mutation based resistance is extremely important, acquisition of ARGs through horizontal gene transfer is of greater concern [71]. Horizontal gene transfer represents the mode of ARG acquisition by many bacterial pathogens, including ESKAPE pathogens [72] and Gram-negative opportunists such as epidemic *E.coli* strains [73].

In regards to species targeted in Map 2, mixed communities are targeted most frequently targeted by studies (32 studies). By taking a whole community approach and investigating mixed populations, the resistome of a particular environment can be explored. *E. coli* are the second most frequently investigated species (13 studies) as they are faecal indicator organism and are often used by surveillance studies of the natural environment to investigate anthropogenic pollution [69].

### Limitations of the map

Limitations of this review are:

1. Searches were undertaken in English and articles were excluded if they were not published in English. This is unlikely to affect Map 2 which was collating publications undertaken in natural environments in the UK. Map 1 was, however, a global map and, therefore, excluding articles not written in English may result in a bias in articles retrieved.
2. Despite best efforts to obtain full texts of all publications designated for full text screening, there were a handful of articles that were not able to be retrieved. There were 30 (7.9% of total full text articles screened) and 6 (3.6% total of full text articles screened) articles for Map 1 and Map 2, respectively. A list of these files can be found in Additional file 7.
3. For Map 1, as discussed previously, we excluded articles which met the inclusion criteria fully except there is no exposure route. We have termed these “sample comparison studies” as they do not show direct evidence of transmission from the environment to humans but show similarities between AMR in environmental niches and humans. This could be as a result of showing direct transmission has occurred is difficult to quantify. As the number of included studies is relatively low for Map 1, we have included a list of the “sample comparison studies” in Additional file 2 as we expect they may be useful for policy makers and researchers alike and should be investigated to provide a better picture on research investigating transmission even though transmission route has not been investigate or only hypothesised.
4. Finally, for both maps, only 10% of references were cross reviewed as a result of the number of search hits retrieved and limited resources available. However, there was 91.8% (Map 1) and 96.1% (Map 2) agreement between reviewers and where disagreements occurred, discussions occurred between the project team to review these.

### Limitations of evidence base

As this was a research mapping exercise, and no critical appraisal was undertaken of gathered research papers. The research gathered and presented here is not necessarily of good quality but does summarise the current research landscape. In addition, these maps could provide a starting point for future evidence synthesis work that includes critical appraisal and meta-analyses.

#### Map 1

As a result of the limited number of studies found globally for Map 1, the evidence base is not distributed evenly across the globe. It is generally, therefore, clustered where researchers are based identified through author affiliations in articles. This may create a bias as to the choice of the surrounding natural environments and socioeconomic status of those affected.

#### Map 2

As a result of limited resources and in response to the funder focus, we limited Map 2 to UK specific studies, with the aim of this being useful for UK policy makers. This means that the results of Map 2 will not be representative of the global research as a whole. Whilst the UK has overwhelmingly studied water environments, this may not be the case elsewhere where a broader coverage of environments may have been studied.

## Conclusions

### Implications for policy and management

Research on environmental AMR is a rapidly growing field and these maps identify and catalogue recent research that investigates environmental AMR and impacts on human health. This is relevant to various decision-makers both globally and in the UK.

The United Nations Environment Programme highlighted the environmental dimension of AMR as one of the most serious environmental pollution issues of our time [19], emphasising AMR alongside plastic pollution at United Nations Environment Assembly (UNEA)-3 in 2017 [74]. Environmental policy is often more focused on chemicals than microorganisms, with the EU Water Framework Directive Priority Substances Watch List identifying antibiotics of concern, in part due to their capacity to select for AMR at environmental concentrations [75, 76]. A recent European Food Safety Authority (EFSA) Opinion on AMR in food producing environments summarised the State of environmental AMR but did not attempt to assess human exposure or transmission risk [77]. To the best of our knowledge this map constitutes the first co-ordinated effort to collate human Exposure and Effect data on AMR in the environment, while other policy facing studies focus on AMR as environmental pollutants or Pressures and the State of AMR abundance and diversity in the environment.

Policy-makers are able to search this map to find evidence that may support or contradict current or planned initiatives to manage environmental AMR and/or public health. For example, there is currently no statutory requirement for structural surveillance of environmental AMR in the UK [78], although the UK government has recently allocated ~ £20 million to develop food-borne and environmental AMR surveillance as part of the PATH-SAFE project [79]. In addition, the UK Environment Agency has recently secured £2 million for the development of an AMR monitoring programme in the environment which will be taking place over the next 2 years [80].

Articles identified by these two maps evidencing transmission from the environment to humans may be used to support the need for surveillance and policy in this area to protect human health. Likewise, this mapping exercise identifies topics where little evidence exists to underpin policy and management, where further research is necessary.

### Implications for research

Knowledge gluts in water and soil environments have been identified, and these areas may be suitable for full systematic review. In addition, identifying knowledge gaps in the research base could help funding bodies to (a) develop grants to target specific under represented areas and (b) identify novel research proposals targeting these under researched areas. Subsequently, primary research could then be conducted to round out the research base with under-represented areas. For example, this could target specific environmental compartments that, to date, have been less frequently researched (e.g. air, animals, soils for Map 1 and air for Map 2).

## Supplementary Information

**Additional file 1.** ROSES for Systematic Map Reports.

**Additional file 2.** Map 1 supplementary articles.

**Additional file 3.** Test set of articles for search benchmarking.

**Additional file 4.** Search strategies.

**Additional file 5.** Unobtainable articles.

**Additional file 6.** Excluded references.

**Additional file 7.** Extraction sheets.

**Additional file 8.** Map 1 interactive geographical map of studies.

**Additional file 9.** Map 2 interactive geographical map of studies.

## Data Availability

All data is provided as additional files.
