# Supplementary material for: Existing evidence on antibiotic resistance exposure and transmission to humans from the environment: a systematic map
Source: Environ Evid. 2022 Mar 12;11:8. doi: 10.1186/s13750-022-00262-2 (PMC8917330; doi:10.1186/s13750-022-00262-2)
Supplement: Supplementary file 3 — Additional file 3. Test set of articles for search benchmarking. [file 13750_2022_262_MOESM3_ESM.docx]

Map 1

Key references

Abdolmaleki, Z., Mashak, Z., & Safarpoor Dehkordi, F. (2019). Phenotypic and genotypic characterization of antibiotic resistance in the methicillin-resistant Staphylococcus aureus strains isolated from hospital cockroaches. *Antimicrob Resist Infect Control, 8*(1), 54. doi:10.1186/s13756-019-0505-7

Chen, M. M., Boardman, W. S., Smith, I., Goodman, A. E., & Brown, M. H. (2015). Characterisation of beta-lactam resistance mediated by blaZ in staphylococci recovered from captive and free-ranging wallabies. *J Glob Antimicrob Resist, 3*(3), 184-189. doi:10.1016/j.jgar.2015.05.002

Gomi, R., Matsuda, T., Matsumura, Y., Yamamoto, M., Tanaka, M., Ichiyama, S., & Yoneda, M. (2017). Whole-Genome Analysis of Antimicrobial-Resistant and Extraintestinal Pathogenic Escherichia coli in River Water. *Appl Environ Microbiol, 83*(5). doi:10.1128/AEM.02703-16

Hatosy, S. M., & Martiny, A. C. (2015). The Ocean as a Global Reservoir of Antibiotic Resistance Genes. *Applied and Environmental Microbiology, 81*(21), 7593-7599. doi:10.1128/aem.00736-15

Leonard, A. F. C., Zhang, L., Balfour, A. J., Garside, R., Hawkey, P. M., Murray, A. K., . . . Gaze, W. H. (2018). Exposure to and colonisation by antibiotic-resistant E. coli in UK coastal water users: Environmental surveillance, exposure assessment, and epidemiological study (Beach Bum Survey). *Environ Int, 114*, 326-333. doi:10.1016/j.envint.2017.11.003

Lorenzin, G., Piccinelli, G., Carlassara, L., Scolari, F., Caccuri, F., Caruso, A., & De Francesco, M. A. (2018). Myroides odoratimimus urinary tract infection in an immunocompromised patient: an emerging multidrug-resistant micro-organism. *Antimicrobial Resistance & Infection Control, 7*(1), 96. doi:10.1186/s13756-018-0391-4

Varela, A. R., Manageiro, V., Ferreira, E., Guimarães, M. A., da Costa, P. M., Caniça, M., & Manaia, C. M. (2015). Molecular evidence of the close relatedness of clinical, gull and wastewater isolates of quinolone-resistant Escherichia coli. *Journal of Global Antimicrobial Resistance, 3*(4), 286-289. doi:10.1016/j.jgar.2015.07.008

Zurfluh, K., Nüesch-Inderbinen, M. T., Poirel, L., Nordmann, P., Hächler, H., & Stephan, R. (2015). Emergence of Escherichia coli producing OXA-48 β-lactamase in the community in Switzerland. *Antimicrobial Resistance and Infection Control, 4*(1), 9. doi:10.1186/s13756-015-0051-x

Map 2

Key references

Amos, G. C. A., Gozzard, E., Carter, C. E., Mead, A., Bowes, M. J., Hawkey, P. M., . . . Wellington, E. M. H. (2015). Validated predictive modelling of the environmental resistome. *The ISME Journal, 9*(6), 1467-1476. doi:10.1038/ismej.2014.237

Dickinson, A. W., Power, A., Hansen, M. G., Brandt, K. K., Piliposian, G., Appleby, P., . . . Vos, M. (2019). Heavy metal pollution and co-selection for antibiotic resistance: A microbial palaeontology approach. *Environment international, 132*, 105117. doi:https://dx.doi.org/10.1016/j.envint.2019.105117

Gaze, W. H., Zhang, L., Abdouslam, N. A., Hawkey, P. M., Calvo-Bado, L., Royle, J., . . . Wellington, E. M. H. (2011). Impacts of anthropogenic activity on the ecology of class 1 integrons and integron-associated genes in the environment. *The ISME Journal, 5*(8), 1253-1261. doi:10.1038/ismej.2011.15

Raven, K. E., Ludden, C., Gouliouris, T., Blane, B., Naydenova, P., Brown, N. M., . . . Peacock, S. J. (2019). Genomic surveillance of Escherichia coli in municipal wastewater treatment plants as an indicator of clinically relevant pathogens and their resistance genes. *Microbial genomics, 5*(5). doi:https://dx.doi.org/10.1099/mgen.0.000267

Xu, Z., Shah, H. N., Misra, R., Chen, J., Zhang, W., Liu, Y., . . . Mkrtchyan, H. V. (2018). The prevalence, antibiotic resistance and mecA characterization of coagulase negative staphylococci recovered from non-healthcare settings in London, UK. *Antimicrobial Resistance & Infection Control, 7*(1), 73. doi:10.1186/s13756-018-0367-4
