# Supplementary material for: Existing evidence on antibiotic resistance exposure and transmission to humans from the environment: a systematic map
Source: Environ Evid. 2022 Mar 12;11:8. doi: 10.1186/s13750-022-00262-2 (PMC8917330; doi:10.1186/s13750-022-00262-2)
Supplement: Supplementary file 4 — Additional file 4. Search strategies. [file 13750_2022_262_MOESM4_ESM.docx]

Search Strategies for Map 1

Project: AMR, Map 1

**Database: Ovid MEDLINE(R) ALL**

Coverage: 1946-present

Date searched: 3^rd^ Feb 2020

Search Strategy:

--------------------------------------------------------------------------------

1 amr.tw. (3127)

2 anti biotic*.tw. (122)

3 antibiotic*.tw. (323419)

4 Enterobacteria*.tw. (23434)

5 antimicrobial*.tw. (154843)

6 microbial*.tw. (161446)

7 antibacter*.tw. (71506)

8 Macrolide*.tw. (15677)

9 betalactam*.tw. (842)

10 Beta lactam*.tw. (42213)

11 Bacter*.tw. (829630)

12 drug*.tw. (1557039)

13 multidrug*.tw. (55241)

14 Resistan*.tw. (965953)

15 Coresist*.tw. (157)

16 Select*.tw. (1846582)

17 Coselect*.tw. (200)

18 Resistome*.tw. (752)

19 Mobilome*.tw. (166)

20 select.tw. (98408)

21 selection.tw. (415255)

22 produce.tw. (407995)

23 producing.tw. (233921)

24 14 or 15 or 17 or 18 or 19 or 20 or 21 or 22 or 23 (1981566)

25 5 or 7 (208242)

26 14 and 25 (70893)

27 2 or 3 or 4 or 6 or 8 or 9 or 10 or 11 or 12 or 13 (2640299)

28 ((Resistan* or Coresist* or Coselect* or Resistome* or Mobilome* or select or selection or produce or producing) adj1 (anti biotic* or antibiotic* or Enterobacteria* or microbial* or Macrolide* or betalactam* or Beta lactam* or Bacter* or drug* or multidrug*)).tw. (185970)

29 1 or 26 or 28 (226730)

30 15 or 16 or 17 or 18 or 19 or 22 or 23 (2398001)

31 ((antimicrobial* or antibacter*) adj1 (Coresist* or Select* or Coselect* or Resistome* or Mobilome* or produce or producing)).tw. (1752)

32 29 or 31 (227717)

33 exp Drug Resistance, Bacterial/ (86572)

34 32 or 33 (267394)

35 human*.tw. (2665887)

36 public*.tw. (556662)

37 patient*.tw. (6498789)

38 clinical*.tw. (3680504)

39 35 or 36 or 37 or 38 (10395551)

40 airborne.tw. (19846)

41 Food*.tw. (446966)

42 water*.tw. (796631)

43 soil*.tw. (150553)

44 wildlife*.tw. (15251)

45 wastewater*.tw. (49292)

46 effluent*.tw. (34586)

47 wetland*.tw. (10845)

48 environment*.tw. (960886)

49 ocean*.tw. (36003)

50 sea.tw. (80443)

51 seas.tw. (2951)

52 aquatic*.tw. (49358)

53 river*.tw. (61358)

54 natural.tw. (533080)

55 stream*.tw. (65904)

56 sewage*.tw. (18974)

57 influent*.tw. (27614)

58 estuar*.tw. (13015)

59 pond*.tw. (16107)

60 or/40-59 (2727634)

61 amr.ti. (113)

62 anti biotic*.ti. (19)

63 antibiotic*.ti. (98978)

64 Enterobacteria*.ti. (7005)

65 antimicrobial*.ti. (55146)

66 microbial*.ti. (47055)

67 antibacter*.ti. (26469)

68 Macrolide*.ti. (4793)

69 betalactam*.ti. (165)

70 lactam*.ti. (18751)

71 Bacter*.ti. (258717)

72 drug*.ti. (393670)

73 multidrug*.ti. (19326)

74 Resistan*.ti. (290979)

75 Coresist*.ti. (22)

76 Select*.ti. (264576)

77 produc*.ti. (392202)

78 or/61-77 (1705728)

79 Expos*.tw. (1152005)

80 Transmi*.tw. (492925)

81 inhal*.tw. (106436)

82 consum*.tw. (452216)

83 contact*.tw. (377910)

84 transfer*.tw. (623617)

85 Infect*.tw. (1681603)

86 impact*.tw. (1043412)

87 health*.tw. (2618250)

88 79 or 80 or 81 or 82 or 83 or 84 or 85 or 86 or 87 (6974105)

89 ((human* or public* or patient* or clinical*) adj4 (Expos* or Transmi* or inhal* or consum* or contact* or transfer* or Infect* or impact* or health*)).tw. (1062235)

90 34 and 60 and 78 and 89 (5217)

91 Comment/ (826657)

92 letter/ (1060619)

93 editorial/ (516499)

94 exp animals/ not humans.sh. (4669577)

95 91 or 92 or 93 or 94 (6408540)

96 90 not 95 (4568)

97 96 (4568)

98 limit 97 to yr="2008 -Current" (3784)

Project: AMR

**Database: CAB Abstracts**

Platform: Ovid SP

Coverage: 1973-present

Date searched: 3^rd^ Feb 2020

Search Strategy:

--------------------------------------------------------------------------------

1 amr.tw. (843)

2 anti biotic*.ti,ab. (67)

3 antibiotic*.ti,ab. (92388)

4 Enterobacteria*.ti,ab. (10562)

5 antimicrobial*.ti,ab. (80871)

6 microbial*.ti,ab. (162834)

7 antibacter*.ti,ab. (42771)

8 Macrolide*.ti,ab. (3321)

9 betalactam*.ti,ab. (110)

10 Beta lactam*.ti,ab. (6769)

11 Bacter*.ti,ab. (439489)

12 drug*.ti,ab. (203564)

13 multidrug*.ti,ab. (10426)

14 Resistan*.ti,ab. (540232)

15 Coresist*.ti,ab. (38)

16 Select*.ti,ab. (790270)

17 Coselect*.ti,ab. (84)

18 Resistome*.ti,ab. (271)

19 Mobilome*.ti,ab. (49)

20 select.ti,ab. (44039)

21 selection.ti,ab. (258333)

22 produce.ti,ab. (202424)

23 producing.ti,ab. (140005)

24 14 or 15 or 17 or 18 or 19 or 20 or 21 or 22 or 23 (1076472)

25 5 or 7 (108695)

26 14 and 25 (29793)

27 2 or 3 or 4 or 6 or 8 or 9 or 10 or 11 or 12 or 13 (780835)

28 ((Resistan* or Coresist* or Coselect* or Resistome* or Mobilome* or select or selection or produce or producing) adj1 (anti biotic* or antibiotic* or Enterobacteria* or microbial* or Macrolide* or betalactam* or Beta lactam* or Bacter* or drug* or multidrug*)).ti,ab. (51999)

29 1 or 26 or 28 (69484)

30 human*.ti,ab. (560823)

31 public*.ti,ab. (228399)

32 patient*.ti,ab. (341676)

33 clinical*.ti,ab. (367315)

34 30 or 31 or 32 or 33 (1262142)

35 airborne.ti,ab. (15995)

36 Food*.ti,ab. (608963)

37 water*.ti,ab. (1099725)

38 soil*.ti,ab. (900682)

39 wildlife*.ti,ab. (35405)

40 wastewater*.ti,ab. (79049)

41 effluent*.ti,ab. (52936)

42 wetland*.ti,ab. (41460)

43 environment*.ti,ab. (806587)

44 ocean*.ti,ab. (36884)

45 sea.ti,ab. (87992)

46 seas.ti,ab. (3167)

47 aquatic*.ti,ab. (75351)

48 river*.ti,ab. (179976)

49 natural.ti,ab. (485511)

50 stream*.ti,ab. (76417)

51 sewage*.ti,ab. (34300)

52 influent*.ti,ab. (20996)

53 estuar*.ti,ab. (23809)

54 pond*.ti,ab. (42916)

55 or/35-54 (3236887)

56 amr.ti. (37)

57 anti biotic*.ti. (9)

58 antibiotic*.ti. (24220)

59 Enterobacteria*.ti. (1806)

60 antimicrobial*.ti. (33431)

61 microbial*.ti. (50742)

62 antibacter*.ti. (15634)

63 Macrolide*.ti. (714)

64 betalactam*.ti. (19)

65 lactam*.ti. (2249)

66 Bacter*.ti. (121583)

67 drug*.ti. (36093)

68 multidrug*.ti. (2907)

69 Resistan*.ti. (184391)

70 Coresist*.ti. (7)

71 Select*.ti. (130630)

72 produc*.ti. (485399)

73 or/56-72 (1011197)

74 Expos*.ti,ab. (384343)

75 Transmi*.ti,ab. (200745)

76 inhal*.ti,ab. (11870)

77 consum*.ti,ab. (432495)

78 contact*.ti,ab. (102333)

79 transfer*.ti,ab. (230691)

80 Infect*.ti,ab. (798876)

81 impact*.ti,ab. (535793)

82 health*.ti,ab. (662800)

83 74 or 75 or 76 or 77 or 78 or 79 or 80 or 81 or 82 (2673331)

84 ((Expos* or Transmi* or inhal* or consum* or contact* or transfer* or Infect* or impact* or health*) adj3 (human* or public* or patient* or clinical*)).ti,ab. (248250)

85 29 and 55 and 73 and 84 (3316)

86 85 (3316)

87 limit 86 to yr="2008 -Current" (2938)

Project: AMR

**Database: Global Health**

Platform: Ovid SP

Coverage: 1973-present

Date searched: 3^rd^ Feb 2020

Search Strategy:

--------------------------------------------------------------------------------

1 amr.tw. (1040)

2 anti biotic*.ti,ab. (67)

3 antibiotic*.ti,ab. (102164)

4 Enterobacteria*.ti,ab. (12901)

5 antimicrobial*.ti,ab. (87022)

6 microbial*.ti,ab. (54210)

7 antibacter*.ti,ab. (36944)

8 Macrolide*.ti,ab. (5641)

9 betalactam*.ti,ab. (279)

10 Beta lactam*.ti,ab. (18840)

11 Bacter*.ti,ab. (232587)

12 drug*.ti,ab. (239766)

13 multidrug*.ti,ab. (22316)

14 Resistan*.ti,ab. (233964)

15 Coresist*.ti,ab. (95)

16 Select*.ti,ab. (266075)

17 Coselect*.ti,ab. (49)

18 Resistome*.ti,ab. (247)

19 Mobilome*.ti,ab. (36)

20 select.ti,ab. (15083)

21 selection.ti,ab. (47611)

22 produce.ti,ab. (56308)

23 producing.ti,ab. (46731)

24 14 or 15 or 17 or 18 or 19 or 20 or 21 or 22 or 23 (365987)

25 5 or 7 (110018)

26 14 and 25 (42184)

27 2 or 3 or 4 or 6 or 8 or 9 or 10 or 11 or 12 or 13 (541631)

28 ((Resistan* or Coresist* or Coselect* or Resistome* or Mobilome* or select or selection or produce or producing) adj1 (anti biotic* or antibiotic* or Enterobacteria* or microbial* or Macrolide* or betalactam* or Beta lactam* or Bacter* or drug* or multidrug*)).ti,ab. (76719)

29 1 or 26 or 28 (98723)

30 human*.ti,ab. (490491)

31 public*.ti,ab. (192213)

32 patient*.ti,ab. (689095)

33 clinical*.ti,ab. (437847)

34 30 or 31 or 32 or 33 (1393939)

35 airborne.ti,ab. (6757)

36 Food*.ti,ab. (348807)

37 water*.ti,ab. (223002)

38 soil*.ti,ab. (38255)

39 wildlife*.ti,ab. (6649)

40 wastewater*.ti,ab. (24045)

41 effluent*.ti,ab. (14497)

42 wetland*.ti,ab. (3861)

43 environment*.ti,ab. (212046)

44 ocean*.ti,ab. (4040)

45 sea.ti,ab. (13573)

46 seas.ti,ab. (327)

47 aquatic*.ti,ab. (13652)

48 river*.ti,ab. (23790)

49 natural.ti,ab. (119345)

50 stream*.ti,ab. (11149)

51 sewage*.ti,ab. (19068)

52 influent*.ti,ab. (7504)

53 estuar*.ti,ab. (2270)

54 pond*.ti,ab. (6277)

55 or/35-54 (816847)

56 amr.ti. (40)

57 anti biotic*.ti. (6)

58 antibiotic*.ti. (24905)

59 Enterobacteria*.ti. (3529)

60 antimicrobial*.ti. (34559)

61 microbial*.ti. (13494)

62 antibacter*.ti. (13476)

63 Macrolide*.ti. (1455)

64 betalactam*.ti. (45)

65 lactam*.ti. (7164)

66 Bacter*.ti. (56610)

67 drug*.ti. (48294)

68 multidrug*.ti. (7118)

69 Resistan*.ti. (85305)

70 Coresist*.ti. (11)

71 Select*.ti. (28872)

72 produc*.ti. (100438)

73 or/56-72 (353952)

74 Expos*.ti,ab. (233578)

75 Transmi*.ti,ab. (150720)

76 inhal*.ti,ab. (14061)

77 consum*.ti,ab. (219935)

78 contact*.ti,ab. (57155)

79 transfer*.ti,ab. (62922)

80 Infect*.ti,ab. (658928)

81 impact*.ti,ab. (213030)

82 health*.ti,ab. (730584)

83 74 or 75 or 76 or 77 or 78 or 79 or 80 or 81 or 82 (1677476)

84 ((Expos* or Transmi* or inhal* or consum* or contact* or transfer* or Infect* or impact* or health*) adj4 (human* or public* or patient* or clinical*)).ti,ab. (391082)

85 29 and 55 and 73 and 84 (3989)

86 85 (3989)

87 limit 86 to yr="2008 -Current" (3484)

Project: AMR

**Database: BIOSIS Citation Index**

Platform: Web of Sciece

Coverage: 1990-present

Date searched: 3^rd^ Feb 2020

TS also searches the major concepts and concept codes so unable to search just the abstract.

Ran the search, downloaded all the results into Endnote, then searched for the human terms in the title or abstract. 6379 refs found but when searched in endnote it went down to 3555. Then searched for amr terms either in title or abstract, down to 3460

--------------------------------------------------------------------------------

TS=("anti biotic*" NEAR/1 (Resistan* or Coresist* or Coselect* or Resistome* or Mobilome* or select or selection or produce or producing)) OR TS=(antibiotic* NEAR/1 (Resistan* or Coresist* or Coselect* or Resistome* or Mobilome* or select or selection or produce or producing)) OR TS=(Enterobacteria* NEAR/1 (Resistan* or Coresist* or Coselect* or Resistome* or Mobilome* or select or selection or produce or producing)) OR TS=(microbial* NEAR/1 (Resistan* or Coresist* or Coselect* or Resistome* or Mobilome* or select or selection or produce or producing)) OR TS=(Macrolide* NEAR/1 (Resistan* or Coresist* or Coselect* or Resistome* or Mobilome* or select or selection or produce or producing)) OR TS=( betalactam* NEAR/1 (Resistan* or Coresist* or Coselect* or Resistome* or Mobilome* or select or selection or produce or producing)) OR TS=(“beta lactam*” NEAR/1 (Resistan* or Coresist* or Coselect* or Resistome* or Mobilome* or select or selection or produce or producing)) OR TS=(Bacter* NEAR/1 (Resistan* or Coresist* or Coselect* or Resistome* or Mobilome* or select or selection or produce or producing)) OR TS=( drug* NEAR/1 (Resistan* or Coresist* or Coselect* or Resistome* or Mobilome* or select or selection or produce or producing)) OR TS=(multidrug* NEAR/1 (Resistan* or Coresist* or Coselect* or Resistome* or Mobilome* or select or selection or produce or producing))

OR

TS=((antimicrobial* OR antibacter*) AND resistan*)

OR

TS=amr

AND

TS=(human*) OR TS=(public*) TS=(patient*) TS=(clinical*)

AND

TS=(airborne OR Food* water* OR soil* OR wildlife* OR wastewater* OR effluent* OR wetland* OR environment* OR ocean* OR sea OR seas OR aquatic* OR river* OR natural OR

stream* OR sewage* OR influent* OR estuar* OR pond*)

AND

TI=(amr OR anti biotic* OR antibiotic* OR Enterobacteria* OR antimicrobial* OR microbial* OR antibacter* OR Macrolide* OR betalactam* OR lactam* OR Bacter* OR drug* OR multidrug* OR Resistan* OR Coresist* OR Select* OR produc*)

AND

TS=(Expos* NEAR/3 (human* or public* or patient* or clinical)) OR TS=(Transmi* NEAR/3 (human* or public* or patient* or clinical)) OR TS=(inhal* NEAR/3 (human* or public* or patient* or clinical)) OR TS=(consum* NEAR/3 (human* or public* or patient* or clinical)) OR TS=(contact* NEAR/3 (human* or public* or patient* or clinical)) OR TS=(transfer* NEAR/3 (human* or public* or patient* or clinical)) OR TS=(Infect* NEAR/3 (human* or public* or patient* or clinical)) OR TS=(impact* NEAR/3 (human* or public* or patient* or clinical)) OR TS=(health* NEAR/3 (human* or public* or patient* or clinical))

Project: AMR

**Database: Web of Science Core Collection**

SCI Expanded 1990-present

SSCI 1956-present

A&HCI 1975-present

CPCI-S 1990-present

CPCI-SSH 1990-present

ESCI 2015-present

Coverage: 1946-present

Date searched: 3^rd^ Feb 2020

--------------------------------------------------------------------------------

TS=("anti biotic*" NEAR/1 (Resistan* or Coresist* or Coselect* or Resistome* or Mobilome* or select or selection or produce or producing)) OR TS=(antibiotic* NEAR/1 (Resistan* or Coresist* or Coselect* or Resistome* or Mobilome* or select or selection or produce or producing)) OR TS=(Enterobacteria* NEAR/1 (Resistan* or Coresist* or Coselect* or Resistome* or Mobilome* or select or selection or produce or producing)) OR TS=(microbial* NEAR/1 (Resistan* or Coresist* or Coselect* or Resistome* or Mobilome* or select or selection or produce or producing)) OR TS=(Macrolide* NEAR/1 (Resistan* or Coresist* or Coselect* or Resistome* or Mobilome* or select or selection or produce or producing)) OR TS=( betalactam* NEAR/1 (Resistan* or Coresist* or Coselect* or Resistome* or Mobilome* or select or selection or produce or producing)) OR TS=(“beta lactam*” NEAR/1 (Resistan* or Coresist* or Coselect* or Resistome* or Mobilome* or select or selection or produce or producing)) OR TS=(Bacter* NEAR/1 (Resistan* or Coresist* or Coselect* or Resistome* or Mobilome* or select or selection or produce or producing)) OR TS=( drug* NEAR/1 (Resistan* or Coresist* or Coselect* or Resistome* or Mobilome* or select or selection or produce or producing)) OR TS=(multidrug* NEAR/1 (Resistan* or Coresist* or Coselect* or Resistome* or Mobilome* or select or selection or produce or producing))

OR

TS=((antimicrobial* OR antibacter*) AND resistan*)

OR

TS=amr

AND

TI=(human*) OR AB=(human) OR TI=(public*) OR AB=(public) OR TI=(patient*) OR AB=(patient) OR TI=(clinical*) OR AB=(clinical*)

AND

TS=(airborne OR Food* water* OR soil* OR wildlife* OR wastewater* OR effluent* OR wetland* OR environment* OR ocean* OR sea OR seas OR aquatic* OR river* OR natural OR

stream* OR sewage* OR influent* OR estuar* OR pond*)

AND

TI=(amr OR anti biotic* OR antibiotic* OR Enterobacteria* OR antimicrobial* OR microbial* OR antibacter* OR Macrolide* OR betalactam* OR lactam* OR Bacter* OR drug* OR multidrug* OR Resistan* OR Coresist* OR Select* OR produc*)

AND

TS=(Expos* NEAR/3 (human* or public* or patient* or clinical)) OR TS=(Transmi* NEAR/3 (human* or public* or patient* or clinical)) OR TS=(inhal* NEAR/3 (human* or public* or patient* or clinical)) OR TS=(consum* NEAR/3 (human* or public* or patient* or clinical)) OR TS=(contact* NEAR/3 (human* or public* or patient* or clinical)) OR TS=(transfer* NEAR/3 (human* or public* or patient* or clinical)) OR TS=(Infect* NEAR/3 (human* or public* or patient* or clinical)) OR TS=(impact* NEAR/3 (human* or public* or patient* or clinical)) OR TS=(health* NEAR/3 (human* or public* or patient* or clinical))

Project: AMR

**Database: SCOPUS**

Platform: Elsevier

Coverage: 1788-present

Date searched: 5^th^ Feb 2020

( ( TITLE-ABS ( amr ) )  OR  ( TITLE-ABS ( antimicrobial*  OR  antibacter* )  AND  resistan* )  OR  ( TITLE-ABS ( "anti biotic*"  W/1  ( resistan*  OR  coresist*  OR  coselect*  OR  resistome*  OR  mobilome*  OR  select  OR  selection  OR  produce  OR  producing ) )  OR  TITLE-ABS ( antibiotic*  W/1  ( resistan*  OR  coresist*  OR  coselect*  OR  resistome*  OR  mobilome*  OR  select  OR  selection  OR  produce  OR  producing ) )  OR  TITLE-ABS ( enterobacteria*  W/1  ( resistan*  OR  coresist*  OR  coselect*  OR  resistome*  OR  mobilome*  OR  select  OR  selection  OR  produce  OR  producing ) )  OR  TITLE-ABS ( microbial*  W/1  ( resistan*  OR  coresist*  OR  coselect*  OR  resistome*  OR  mobilome*  OR  select  OR  selection  OR  produce  OR  producing ) )  OR  TITLE-ABS ( macrolide*  W/1  ( resistan*  OR  coresist*  OR  coselect*  OR  resistome*  OR  mobilome*  OR  select  OR  selection  OR  produce  OR  producing ) )  OR  TITLE-ABS ( betalactam*  W/1  ( resistan*  OR  coresist*  OR  coselect*  OR  resistome*  OR  mobilome*  OR  select  OR  selection  OR  produce  OR  producing ) )  OR  TITLE-ABS ( "beta lactam*"  W/1  ( resistan*  OR  coresist*  OR  coselect*  OR  resistome*  OR  mobilome*  OR  select  OR  selection  OR  produce  OR  producing ) )  OR  TITLE-ABS ( bacter*  W/1  ( resistan*  OR  coresist*  OR  coselect*  OR  resistome*  OR  mobilome*  OR  select  OR  selection  OR  produce  OR  producing ) )  OR  TITLE-ABS ( drug*  W/1  ( resistan*  OR  coresist*  OR  coselect*  OR  resistome*  OR  mobilome*  OR  select  OR  selection  OR  produce  OR  producing ) )  OR  TITLE-ABS ( multidrug*  W/1  ( resistan*  OR  coresist*  OR  coselect*  OR  resistome*  OR  mobilome*  OR  select  OR  selection  OR  produce  OR  producing ) ) ) )  AND  ( TITLE-ABS ( airborne  OR  food*  OR  water*  OR  soil*  OR  wildlife*  OR  wastewater*  OR  effluent*  OR  wetland*  OR  environment*  OR  ocean*  OR  sea  OR  seas  OR  aquatic*  OR  river*  OR  natural  OR  stream*  OR  sewage*  OR  influent*  OR  estuar*  OR  pond* ) )  AND  ( TITLE-ABS ( expos*  W/2  ( human*  OR  public*  OR  patient*  OR  clinical* ) )  OR  TITLE-ABS ( transmi*  W/2  ( human*  OR  public*  OR  patient*  OR  clinical* ) )  OR  TITLE-ABS ( inhal*  W/2  ( human*  OR  public*  OR  patient*  OR  clinical* ) )  OR  TITLE-ABS ( consum*  W/2  ( human*  OR  public*  OR  patient*  OR  clinical* ) )  OR  TITLE-ABS ( contact*  W/2  ( human*  OR  public*  OR  patient*  OR  clinical* ) )  OR  TITLE-ABS ( transfer*  W/2  ( human*  OR  public*  OR  patient*  OR  clinical* ) )  OR  TITLE-ABS ( infect*  W/2  ( human*  OR  public*  OR  patient*  OR  clinical* ) )  OR  TITLE-ABS ( impact*  W/2  ( human*  OR  public*  OR  patient*  OR  clinical* ) )  OR  TITLE-ABS ( health*  W/2  ( human*  OR  public*  OR  patient*  OR  clinical* ) ) )  AND  ( TITLE ( amr  OR  "anti biotic*"  OR  antibiotic*  OR  enterobacteria*  OR  antimicrobial*  OR  microbial*  OR  antibacter*  OR  macrolide*  OR  betalactam*  OR  lactam*  OR  bacter*  OR  drug*  OR  multidrug* ) )

Project: AMR

**Database: GREEFile**

Platform:EBSCOHost

Coverage: No dates given

Date searched: 4th Feb 2020

--------------------------------------------------------------------------------

TI("anti biotic*" N1 (Resistan* or Coresist* or Coselect* or Resistome* or Mobilome* or select or selection or produce or producing)) OR TI(antibiotic* N1 (Resistan* or Coresist* or Coselect* or Resistome* or Mobilome* or select or selection or produce or producing)) OR TI(Enterobacteria* N1 (Resistan* or Coresist* or Coselect* or Resistome* or Mobilome* or select or selection or produce or producing)) OR TI(microbial* N1 (Resistan* or Coresist* or Coselect* or Resistome* or Mobilome* or select or selection or produce or producing)) OR TI(Macrolide* N1 (Resistan* or Coresist* or Coselect* or Resistome* or Mobilome* or select or selection or produce or producing)) OR TI( betalactam* N1 (Resistan* or Coresist* or Coselect* or Resistome* or Mobilome* or select or selection or produce or producing)) OR TI(“beta lactam*” N1 (Resistan* or Coresist* or Coselect* or Resistome* or Mobilome* or select or selection or produce or producing)) OR TI(Bacter* N1 (Resistan* or Coresist* or Coselect* or Resistome* or Mobilome* or select or selection or produce or producing)) OR TI( drug* N1 (Resistan* or Coresist* or Coselect* or Resistome* or Mobilome* or select or selection or produce or producing)) OR TI(multidrug* N1 (Resistan* or Coresist* or Coselect* or Resistome* or Mobilome* or select or selection or produce or producing))

OR

TI ((antimicrobial* OR antibacter*) AND resistan*) OR AB ((antimicrobial* OR antibacter*) AND resistan*)

OR

TI amr OR AB amr

AND

TI (Human* OR public* OR patient* OR clinical*) OR AB (Human* OR public* OR patient* OR clinical*)

AND

TI(airborne OR Food* OR water* OR soil* OR wildlife* OR wastewater* OR effluent* OR wetland* OR environment* OR ocean* OR sea OR seas OR aquatic* OR river* OR natural OR

stream* OR sewage* OR influent* OR estuar* OR pond*) OR AB (airborne OR Food* OR water* OR soil* OR wildlife* OR wastewater* OR effluent* OR wetland* OR environment* OR ocean* OR sea OR seas OR aquatic* OR river* OR natural OR stream* OR sewage* OR influent* OR estuar* OR pond*)

AND

TI (Expos* or Transmi* or inhal* or consum* or contact* or transfer* or Infect* or impact* or health*) OR AB (Expos* or Transmi* or inhal* or consum* or contact* or transfer* or Infect* or impact* or health*)

Project: AMR

**Database: Environment Complete**

Platform:EBSCOHost

Coverage: 1888-present

Date searched: 4th Feb 2020

--------------------------------------------------------------------------------

TI("anti biotic*" N1 (Resistan* or Coresist* or Coselect* or Resistome* or Mobilome* or select or selection or produce or producing)) OR TI(antibiotic* N1 (Resistan* or Coresist* or Coselect* or Resistome* or Mobilome* or select or selection or produce or producing)) OR TI(Enterobacteria* N1 (Resistan* or Coresist* or Coselect* or Resistome* or Mobilome* or select or selection or produce or producing)) OR TI(microbial* N1 (Resistan* or Coresist* or Coselect* or Resistome* or Mobilome* or select or selection or produce or producing)) OR TI(Macrolide* N1 (Resistan* or Coresist* or Coselect* or Resistome* or Mobilome* or select or selection or produce or producing)) OR TI( betalactam* N1 (Resistan* or Coresist* or Coselect* or Resistome* or Mobilome* or select or selection or produce or producing)) OR TI(“beta lactam*” N1 (Resistan* or Coresist* or Coselect* or Resistome* or Mobilome* or select or selection or produce or producing)) OR TI(Bacter* N1 (Resistan* or Coresist* or Coselect* or Resistome* or Mobilome* or select or selection or produce or producing)) OR TI( drug* N1 (Resistan* or Coresist* or Coselect* or Resistome* or Mobilome* or select or selection or produce or producing)) OR TI(multidrug* N1 (Resistan* or Coresist* or Coselect* or Resistome* or Mobilome* or select or selection or produce or producing))

OR

TI ((antimicrobial* OR antibacter*) AND resistan*) OR AB ((antimicrobial* OR antibacter*) AND resistan*)

OR

TI amr OR AB amr

AND

TI (Human* OR public* OR patient* OR clinical*) OR AB (Human* OR public* OR patient* OR clinical*)

AND

TI(airborne OR Food* OR water* OR soil* OR wildlife* OR wastewater* OR effluent* OR wetland* OR environment* OR ocean* OR sea OR seas OR aquatic* OR river* OR natural OR

stream* OR sewage* OR influent* OR estuar* OR pond*) OR AB (airborne OR Food* OR water* OR soil* OR wildlife* OR wastewater* OR effluent* OR wetland* OR environment* OR ocean* OR sea OR seas OR aquatic* OR river* OR natural OR stream* OR sewage* OR influent* OR estuar* OR pond*)

AND

TI (Expos* or Transmi* or inhal* or consum* or contact* or transfer* or Infect* or impact* or health*) OR AB (Expos* or Transmi* or inhal* or consum* or contact* or transfer* or Infect* or impact* or health*)

Project: AMR
**Database: Epistemonikos**
Platform: Website: https://www.epistemonikos.org/en/
Coverage: No dates given
Date searched: 3^rd^ Feb 2020

Only systematic reviews. Downloaded searches separately into one endnote library and de-duped in there

title:(title:(amr OR "anti biotic*" OR antibiotic* OR enterobacteria* OR antimicrobial* OR microbial* OR antibacter* OR macrolide* OR betalactam* OR lactam* OR bacter* OR drug* OR multidrug*)) AND (title:(airborne OR Food* OR water* OR soil* OR wildlife* OR wastewater* OR effluent* OR wetland* OR environment* OR ocean* OR sea OR seas OR aquatic* OR river* OR natural OR stream* OR sewage* OR influent* OR estuar* OR pond*) OR abstract:(airborne OR Food* OR water* OR soil* OR wildlife* OR wastewater* OR effluent* OR wetland* OR environment* OR ocean* OR sea OR seas OR aquatic* OR river* OR natural OR stream* OR sewage* OR influent* OR estuar* OR pond*)) AND (title:(human* OR public* OR patient* OR clinical*) OR abstract:(human* OR public* OR patient* OR clinical*)) 437

OR

title:(title:(amr OR "anti biotic*" OR antibiotic* OR enterobacteria* OR antimicrobial* OR microbial* OR antibacter* OR macrolide* OR betalactam* OR lactam* OR bacter* OR drug* OR multidrug*)) AND (title:(airborne OR Food* OR water* OR soil* OR wildlife* OR wastewater* OR effluent* OR wetland* OR environment* OR ocean* OR sea OR seas OR aquatic* OR river* OR natural OR stream* OR sewage* OR influent* OR estuar* OR pond*) OR abstract:(airborne OR Food* OR water* OR soil* OR wildlife* OR wastewater* OR effluent* OR wetland* OR environment* OR ocean* OR sea OR seas OR aquatic* OR river* OR natural OR stream* OR sewage* OR influent* OR estuar* OR pond*)) AND title:(Expos* or Transmi* or inhal* or consum* or contact* or transfer* or Infect* or impact* or health*) OR abstract: :(Expos* or Transmi* or inhal* or consum* or contact* or transfer* or Infect* or impact* or health*)

Project: AMR

**Database: ProQuest Dissertations and Theses**

Platform: ProQuest

Coverage: 1861-present

Date searched: 5th Feb 2020

TI("anti biotic*" Near/1 (Resistan* or Coresist* or Coselect* or Resistome* or Mobilome* or select or selection or produce or producing)) OR TI(antibiotic* NEAR/1 (Resistan* or Coresist* or Coselect* or Resistome* or Mobilome* or select or selection or produce or producing)) OR TI(Enterobacteria* NEAR/1 (Resistan* or Coresist* or Coselect* or Resistome* or Mobilome* or select or selection or produce or producing)) OR TI(microbial* NEAR/1 (Resistan* or Coresist* or Coselect* or Resistome* or Mobilome* or select or selection or produce or producing)) OR TI(Macrolide* NEAR/1 (Resistan* or Coresist* or Coselect* or Resistome* or Mobilome* or select or selection or produce or producing)) OR TI( betalactam* NEAR/1 (Resistan* or Coresist* or Coselect* or Resistome* or Mobilome* or select or selection or produce or producing)) OR TI(“beta lactam*” NEAR/1 (Resistan* or Coresist* or Coselect* or Resistome* or Mobilome* or select or selection or produce or producing)) OR TI(Bacter* NEAR/1 (Resistan* or Coresist* or Coselect* or Resistome* or Mobilome* or select or selection or produce or producing)) OR TI( drug* NEAR/1 (Resistan* or Coresist* or Coselect* or Resistome* or Mobilome* or select or selection or produce or producing)) OR TI(multidrug* NEAR/1 (Resistan* or Coresist* or Coselect* or Resistome* or Mobilome* or select or selection or produce or producing))

OR

AB("anti biotic*" NEAR/1 (Resistan* or Coresist* or Coselect* or Resistome* or Mobilome* or select or selection or produce or producing)) OR AB(antibiotic* NEAR/1 (Resistan* or Coresist* or Coselect* or Resistome* or Mobilome* or select or selection or produce or producing)) OR AB(Enterobacteria* NEAR/1 (Resistan* or Coresist* or Coselect* or Resistome* or Mobilome* or select or selection or produce or producing)) OR AB(microbial* NEAR/1 (Resistan* or Coresist* or Coselect* or Resistome* or Mobilome* or select or selection or produce or producing)) OR AB(Macrolide* NEAR/1 (Resistan* or Coresist* or Coselect* or Resistome* or Mobilome* or select or selection or produce or producing)) OR AB( betalactam* NEAR/1 (Resistan* or Coresist* or Coselect* or Resistome* or Mobilome* or select or selection or produce or producing)) OR AB(“beta lactam*” NEAR/1 (Resistan* or Coresist* or Coselect* or Resistome* or Mobilome* or select or selection or produce or producing)) OR AB(Bacter* NEAR/1 (Resistan* or Coresist* or Coselect* or Resistome* or Mobilome* or select or selection or produce or producing)) OR AB( drug* NEAR/1 (Resistan* or Coresist* or Coselect* or Resistome* or Mobilome* or select or selection or produce or producing)) OR AB(multidrug* NEAR/1 (Resistan* or Coresist* or Coselect* or Resistome* or Mobilome* or select or selection or produce or producing))

OR

TI(antimicrobial* OR antibacter*) AND TI(resistan*) OR AB(antimicrobial* OR antibacter*) AND AB(resistan*)

OR

TI(amr) OR AB(amr)

AND

TI (airborne OR Food* OR water* OR soil* OR wildlife* OR wastewater* OR effluent* OR wetland* OR environment* OR ocean* OR sea OR seas OR aquatic* OR river* OR natural OR

stream* OR sewage* OR influent* OR estuar* OR pond*)

AND

TI( amr  OR  "anti biotic*"  OR  antibiotic*  OR  enterobacteria*  OR  antimicrobial*  OR  microbial*  OR  antibacter*  OR  macrolide*  OR  betalactam*  OR  lactam*  OR  bacter*  OR  drug*  OR  multidrug* )

Project: AMR
**Database: Explore the British Library**
Platform: Website: http://explore.bl.uk/primo_library/libweb/action/search.do?vid=BLVU1#
Coverage: No dates given
Date searched: 5th Feb 2020

Antimicrob*(title) and resist* (anywhere) =theses 111 hits, reports = 0. Downloaded to endnote and did further search as it didn’t perform the search as written (didn’t all have antimicrob* in title), searched for this in endnote, 64 hits and downloaded into endnote screening library

Search Strategies for Map 2

Project: AMR, Map 2

**Database: AGRIS**

Platforms: FAO website. <https://agris.fao.org/agris-search/info.action>

Coverage: No date given

Date searched: 31^st^ Oct 2019

antimicrobial) +(resistant) +(human) +(health) +(air) 4

antimicrobial) +(resistant) +(human) +(health) +(water) 58

antimicrobial) +(resistant) +(human) +(health) +(soil) 12

antimicrobial) +(resistant) +(human) +(health) +(nature) 9

antimicrobial) +(resistant) +(human) +(health) +(environment) 106

Project: AMR, Map 2

**Database: BIOSIS Citation Index (1969-present) and Web of Science Core Collection**

SCI Expanded 1990-present

SSCI 1956-present

A&HCI 1975-present

CPCI-S 1990-present

CPCI-SSH 1990-present

ESCI 2015-present

Platforms: Clarivate

Coverage:

Date searched: 31^st^ Oct 2019

TS=(amr)

OR

TS=(anti biotic* NEAR/2 resist*) Or TS=(anti biotic* NEAR/2 coresist*) Or TS=(anti biotic* NEAR/2 select*) Or TS=(anti biotic* NEAR/2 resistome*) Or TS=(anti biotic* NEAR/2 coselect*) Or TS=(anti biotic* NEAR/2 mobilome*)

OR

TS=(antibiotic* NEAR/2 resist*) Or TS=(antibiotic* NEAR/2 coresist*) Or TS=(antibiotic* NEAR/2 select*) Or TS=(antibiotic* NEAR/2 resistome*) Or TS=(antibiotic* NEAR/2 coselect*) or TS=(antibiotic* NEAR/2 mobilome*)

OR

TS=(enterobacteri* NEAR/2 resist*) Or TS=(enterobacteri* NEAR/2 coresist*) Or TS=(enterobacteri* NEAR/2 select*) Or TS=(enterobacteri* NEAR/2 resistome*) Or TS=(enterobacteri* NEAR/2 coselect*) Or TS=(enterobacteri* NEAR/2 mobilome*)

OR

TS=(entero bacteri* NEAR/2 resist*) Or TS=(entero bacteri* NEAR/2 coresist*) Or TS=(entero bacteri* NEAR/2 select*) Or TS=(entero bacteri* NEAR/2 resistome*) Or TS=(entero bacteri* NEAR/2 coselect*) Or TS=(entero bacteri* NEAR/2 mobilome*)

OR

TS=(antibacter*NEAR/2 resist*) Or TS=(antibacter*NEAR/2 coresist*) Or TS=(antibacter*NEAR/2 select*) Or TS=(antibacter*NEAR/2 resistome*) Or TS=(antibacter*NEAR/2 coselect*) Or TS=(antibacter*NEAR/2 mobilome*)

OR

TS=(anti bacter*NEAR/2 resist*) Or TS=(anti bacter*NEAR/2 coresist*) Or TS=(anti bacter*NEAR/2 select*) Or TS=(anti bacter*NEAR/2 resistome*) Or TS=(anti bacter*NEAR/2 coselect*) Or TS=(anti bacter*NEAR/2 mobilome*)

OR

TS=(microbial*NEAR/2 resist*) Or TS=(microbial*NEAR/2 coresist*) Or TS=(microbial*NEAR/2 select*) Or TS=(microbial*NEAR/2 resistome*) Or TS=(microbial*NEAR/2 coselect*) Or TS=(microbial*NEAR/2 mobilome*)

OR

TS=(Macrolide*NEAR/2 resist*) Or TS=(Macrolide*NEAR/2 coresist*) Or TS=(Macrolide*NEAR/2 select*) Or TS=(Macrolide*NEAR/2 resistome*) Or TS=(Macrolide*NEAR/2 coselect*) Or TS=(Macrolide*NEAR/2 mobilome*)

OR

TS=(Betalactam*NEAR/2 resist*) Or TS=(Betalactam*NEAR/2 coresist*) Or TS=(Betalactam*NEAR/2 select*) Or TS=(Betalactam*NEAR/2 resistome*) Or TS=(Betalactam*NEAR/2 coselect*) Or TS=(Betalactam*NEAR/2 mobilome*)

OR

TS=(Beta lactam*NEAR/2 resist*) Or TS=(Beta lactam*NEAR/2 coresist*) Or TS=(Beta lactam*NEAR/2 select*) Or TS=(Beta lactam*NEAR/2 resistome*) Or TS=(Beta lactam*NEAR/2 coselect*) Or TS=(Beta lactam*NEAR/2 mobilome*)

AND

TS=(water* OR soil* OR wastewater* OR air* OR effluent* OR wetland* OR aquatic* OR river* OR sediment* OR lake* OR sea* OR sewage* OR influent* OR environment* OR natur*)

AND

TS=("national health service" or nhs)

OR

TS=(gb or "g.b." or uk or "u.k." or “northern ireland*” or “nothern irish*” or scotland* or scottish* or welsh)

OR

AD=("national health service" or nhs)

OR

AD=(gb or "g.b." or uk or "u.k." or “northern ireland*” or “nothern irish*” or scotland* or scottish* or welsh)

OR

TS=(britain or (british not "british columbia"))

OR

TS=(united kingdom* or (england not "new england"))

OR

TS=((wales or "south wales") not "new south wales")

OR

AD=(britain or (british not "british columbia"))

OR

AD=(united kingdom* or (england not "new england"))

OR

AD=((wales or "south wales") not "new south wales")

Project: AMR, Map 2

**Database: CAB Abstracts and Global Health**

Platforms: OVIDSp

Coverage: 1973-present

Date searched: 31^st^ Oct 2019

1 amr.tw. (787)

2 anti biotic*.tw. (65)

3 antibiotic*.tw. (174399)

4 Enterobacteria*.tw. (181656)

5 entero bacteria*.tw. (14)

6 antimicrobial*.tw. (84574)

7 microbial*.tw. (270949)

8 antibacter*.tw. (80722)

9 anti bacter*.tw. (2266)

10 Macrolide*.tw. (14702)

11 betalactam*.tw. (109)

12 beta lactam*.tw. (38719)

13 resist*.tw. (620924)

14 coresist*.tw. (35)

15 select*.tw. (793960)

16 resistome*.tw. (252)

17 coselect*.tw. (84)

18 mobilome*.tw. (45)

19 13 or 14 or 15 or 16 or 17 or 18 (1311458)

20 ((resist* or coresist* or select* or resistome* or coselect* or mobilome*) adj2 (anti biotic* or antibiotic* or Enterobacteria* or entero bacteria* or antimicrobial* or microbial* or antibacter* or anti bacter* or Macrolide* or betalactam* or beta lactam*)).tw. (28903)

21 uk/ or british isles/ (178765)

22 ("national health service" or nhs).ti,ab,in. (3548)

23 (gb or "g.b." or britain or (british not "british columbia") or uk or "u.k." or united kingdom* or (england not "new england") or northern ireland* or nothern irish* or scotland* or scottish* or ((wales or "south wales") not "new south wales") or welsh*).ti,ab,jw,in. (531777)

24 21 or 22 or 23 (561637)

25 water*.tw. (1335888)

26 soil*.tw. (984018)

27 wastewater*.tw. (117172)

28 air*.tw. (259186)

29 effluent*.tw. (63830)

30 wetland*.tw. (59237)

31 aquatic*.tw. (437305)

32 river*.tw. (188245)

33 sediment*.tw. (119219)

34 lake*.tw. (109884)

35 sea*.tw. (705843)

36 sewage*.tw. (45001)

37 influent*.tw. (22435)

38 environment*.tw. (988113)

39 natur*.tw. (940214)

40 1 or 20 (29119)

41 25 or 26 or 27 or 28 or 29 or 30 or 31 or 32 or 33 or 34 or 35 or 36 or 37 or 38 or 39 (3840346)

42 40 and 41 (11630)

43 soil biology/ (9542)

44 exp water/ (433392)

45 air microbiology/ (1046)

46 exp wild animals/ (82528)

47 41 or 46 (3880059)

48 24 and 40 and 47 (601)

Project: AMR, Map 2

**Database: GREEFile (no date given) and Environment Complete (1888-present)**

Platforms: EBSCOHost

Coverage:

Date searched: 5^th^ Nov 2019

TI=amr or AB=amr or KW=AMR

OR

TI antibiotic* OR TI enterobacteri* OR TI “entero bacteri*” OR TI antibacter* OR TI “anti bacter*” OR TI microbial* OR TI Betalactam OR TI “Beta lactam*”

OR

AB antibiotic* OR AB enterobacteri* OR AB “entero bacteri*” OR AB antibacter* OR AB “anti bacter*” OR AB microbial* OR AB Betalactam OR AB “Beta lactam*”

OR

KW antibiotic* OR KW enterobacteri* OR KW “entero bacteri*” OR KW antibacter* OR KW “anti bacter*” OR KW microbial* OR KW Betalactam OR KW “Beta lactam*”)

AND

TI resist* or TI coresist or TI select* or TI resistom* or TI coselect* or TI mobilome*

AB resist* or AB coresist or AB select* or AB resistom* or AB coselect* or AB mobilome*

KW resist* or KW coresist or KW select* or KW resistom* or KW coselect* or KW mobilome*

AND

TI water* OR TI soil* OR TI wastewater* OR TI air* OR TI effluent* OR TI wetland* OR TI aquatic* OR TI river* OR TI sediment* OR TI lake* OR TI sea* OR TI sewage* OR TI influent* OR TI environment* OR TI natur*

AND

TI "national health service" or TI nhs or AB "national health service" or AB nhs or TI gb or TI "g.b." or TI uk or TI "u.k." or TI “northern ireland*” or TI “nothern irish*” or TI scotland* or TI scottish* or TI welsh or AB gb or AB "g.b." or AB uk or AB "u.k." or AB “northern ireland*” or AB “nothern irish*” or AB scotland* or AB scottish* or AB welsh

or

MH gb or MH "g.b." or MH uk or MH "u.k." or MH “northern ireland*” or MH “nothern irish*” or MH scotland* or MH scottish* or MH welsh

or

AFFIL("g.b" or uk or "u.k." or "northern Ireland* or "northern irish*" or scotland* or scottish* or welsh or britain or "united kingdom*" )

or

TI britain or AB Britain or MH Britain

or

TI (british not "british columbia")

or

AB (british not "british columbia")

or

MH (british not "british columbia")

or

TI “united kingdom*” or AB “united kingdom*” or MH “united kingdom*”

or

TI (england not "new england") or AB (england not "new england") or MH (england not "new england")

or

TI ((wales or "south wales") not "new south wales")

or

AB ((wales or "south wales") not "new south wales")

or

MH ((wales or "south wales") not "new south wales")

With systematic review filter on left

Project: AMR, Map 2

**Database: Epistemonikos**

Platforms: Website. <https://www.epistemonikos.org/>

Coverage: Not given

Date: 5^th^ Nov 2019

(title:(antimicrob* resist*) OR abstract:(antimicrob* resist*)) AND (title:(water*) OR abstract:(water*)) 6

(title:(antimicrob* resist*) OR abstract:(antimicrob* resist*)) AND (title:(air*) OR abstract:(air*)) 4

(title:(antimicrob* resist*) OR abstract:(antimicrob* resist*)) AND (title:(soil*) OR abstract:(soil*)) 1 (already captured)

(title:(antimicrob* resist*) OR abstract:(antimicrob* resist*)) AND (title:(environment*) OR abstract:(environment*)) 28

(title:(antimicrob* resist*) OR abstract:(antimicrob* resist*)) AND (title:(natur*) OR abstract:(natur*)) 28

Project: AMR, Map 2

**Database: Explore the British Library**

Platforms: Website. <http://explore.bl.uk/primo_library/libweb/action/search.do?vid=BLVU1>

Coverage: Not given

Date: 5^th^ Nov 2019

Antimicrobial (Anywhere)

AND

Resist* anywhere

Restricted to:

Reports (14)

Theses: 117

Project: AMR, Map 2

**Database: Google Scholar via Publish or Perish**

Date: 5^th^ Nov 2019

antimicrobial resistance and UK = in title = 65. In keywords = 200

Project: AMR, Map 2

**Database: Medline ALL**

Platforms: OVIDSp

Coverage: 1946-present

Date: 30^th^ Oct 2019

1. amr.tw. (2883)

2 anti biotic*.tw. (123)

3 antibiotic*.tw. (316751)

4 Enterobacteria*.tw. (22881)

5 entero bacteria*.tw. (28)

6 antimicrobial*.tw. (150300)

7 microbial*.tw. (156238)

8 antibacter*.tw. (69500)

9 anti bacter*.tw. (3258)

10 Macrolide*.tw. (15393) classes of antibiotic

11 betalactam*.tw. (838)

12 beta lactam*.tw. (41415)

13 resist*.tw. (984707)

14 coresist*.tw. (152)

15 select*.tw. (1809631)

16 resistome*.tw. (671) genetic expression terms

17 coselect*.tw. (197)

18 mobilome*.tw. (158)

19 13 or 14 or 15 or 16 or 17 or 18 (2678427)

20 ((resist* or coresist* or select* or resistome* or coselect* or mobilome*) **adj2** (anti biotic* or antibiotic* or Enterobacteria* or entero bacteria* or antimicrobial* or microbial* or antibacter* or anti bacter* or Macrolide* or betalactam* or beta lactam*)).tw. (74780)

21 drug resistance, microbial/ or exp drug resistance, bacterial/ (139706)

22 exp United Kingdom/ (356927)

23 ("national health service" or nhs).ti,ab,in. (177209)

24 (gb or "g.b." or britain or (british not "british columbia") or uk or "u.k." or united kingdom* or (england not "new england") or northern ireland* or nothern irish* or scotland* or scottish* or ((wales or "south wales") not "new south wales") or welsh*).ti,ab,jw,in. (1968943)

25 22 or 23 or 24 (2146128)

26 water*.tw. (779639)

27 soil*.tw. (146235)

28 wastewater*.tw. (47494)

29 air*.tw. (425393)

30 effluent*.tw. (33874)

31 wetland*.tw. (10484)

32 aquatic*.tw. (47732)

33 river*.tw. (59520)

34 sediment*.tw. (96575)

35 lake*.tw. (36397)

36 sea*.tw. (752700)

37 sewage*.tw. (18556)

38 influent*.tw. (26766)

39 environment*.tw. (935167)

40 natur*.tw. (1019126)

41 exp Environmental Microbiology/ (111162)

42 26 or 27 or 28 or 29 or 30 or 31 or 32 or 33 or 34 or 35 or 36 or 37 or 38 or 39 or 40 or 41 (3650978)

43 1 or 20 or 21 (178476)

44 25 and 42 and 43 (2371)

45 limit 44 to yr="2005 -Current" (1810)

Project: AMR, Map 2

**Database: PQDT**

Platforms: ProQuest

Coverage: 1861-present

Date: 30^th^ Oct 2019

TI(anti biotic* NEAR/2 resist*) Or TI(anti biotic* NEAR/2 coresist*) Or TI(anti biotic* NEAR/2 select*) Or TI(anti biotic* NEAR/2 resistome*) Or TI(anti biotic* NEAR/2 coselect*) Or TI(anti biotic* NEAR/2 mobilome*)

OR
AB(antibiotic* NEAR/2 resist*) Or AB(antibiotic* NEAR/2 coresist*) Or AB(antibiotic* NEAR/2 select*) Or AB(antibiotic* NEAR/2 resistome*) Or AB(antibiotic* NEAR/2 coselect*) Or AB(antibiotic* NEAR/2 mobilome*)

OR

TI(enterobacteri* NEAR/2 resist*) Or TI(enterobacteri* NEAR/2 coresist*) Or TI(enterobacteri* NEAR/2 select*) Or TI(enterobacteri* NEAR/2 resistome*) Or TI(enterobacteri* NEAR/2 coselect*) Or TI(enterobacteri* NEAR/2 mobilome*)

OR

AB(enterobacteri* NEAR/2 resist*) Or AB(enterobacteri* NEAR/2 coresist*) Or AB(enterobacteri* NEAR/2 select*) Or AB(enterobacteri* NEAR/2 resistome*) Or AB(enterobacteri* NEAR/2 coselect*) Or AB(enterobacteri* NEAR/2 mobilome*)

OR

TI(entero bacteri* NEAR/2 resist*) Or TI(entero bacteri* NEAR/2 coresist*) Or TI(entero bacteri* NEAR/2 select*) Or TI(entero bacteri* NEAR/2 resistome*) Or TI(entero bacteri* NEAR/2 coselect*) Or TI(entero bacteri* NEAR/2 mobilome*)

OR

AB(entero bacteri* NEAR/2 resist*) Or AB(entero bacteri* NEAR/2 coresist*) Or AB(entero bacteri* NEAR/2 select*) Or AB(entero bacteri* NEAR/2 resistome*) Or AB(entero bacteri* NEAR/2 coselect*) Or AB(entero bacteri* NEAR/2 mobilome*)

OR

TI(antibacter*NEAR/2 resist*) Or TI(antibacter*NEAR/2 coresist*) Or TI(antibacter*NEAR/2 select*) Or TI(antibacter*NEAR/2 resistome*) Or TI(antibacter*NEAR/2 coselect*) Or TI(antibacter*NEAR/2 mobilome*) 0

OR

TI(microbial*NEAR/2 resist*) Or TI(microbial*NEAR/2 coresist*) Or TI(microbial*NEAR/2 select*) Or TI(microbial*NEAR/2 resistome*) Or TI(microbial*NEAR/2 coselect*) Or TI(microbial*NEAR/2 mobilome*)

OR

TI(Macrolide*NEAR/2 resist*) Or TI(Macrolide*NEAR/2 coresist*) Or TI(Macrolide*NEAR/2 select*) Or TI(Macrolide*NEAR/2 resistome*) Or TI(Macrolide*NEAR/2 coselect*) Or TI(Macrolide*NEAR/2 mobilome*)

OR

TI(water* OR soil* OR wastewater* OR air* OR effluent* OR wetland* OR aquatic* OR river* OR sediment* OR lake* OR sea* OR sewage* OR influent* OR environment* OR natur*)

AND

TI("national health service" or nhs or gb or "g.b." or uk or "u.k." or “northern ireland*” or “nothern irish*” or scotland* or scottish* or welsh)

OR

TI britain or “united kingdom*”

AB britain or “united kingdom*”

LOC britain or “united kingdom*”

ULO britain or “united kingdom*”

OR

TI (british not "british columbia")

AB (british not "british columbia")

LOC (british not "british columbia")

ULO (british not "british columbia")

OR

TI (england not "new england")

AB (england not "new england")

LOC (england not "new england")

ULO (england not "new england")

OR

TI ((wales or "south wales") not "new south wales")

AB ((wales or "south wales") not "new south wales")

LOC ((wales or "south wales") not "new south wales")

ULO ((wales or "south wales") not "new south wales")

Project: AMR, Map 2

**Database: SCOPUS**

Platforms: Elsevier

Coverage: 1788-present

Date: 5^th^ Nov 2019

( ( ( TITLE-ABS-KEY ( amr ) )  OR  ( TITLE-ABS-KEY ( ( "anti biotic"  OR  antibiotic*  OR  enterobacteria*  OR  "entero bacteria*"  OR  antimicrobial*  OR  microbial*  OR  antibacter*  OR  "anti bacter*"  OR  macrolide*  OR  betalactam*  OR  "beta lactam*" )  W/2  resist* ) )  OR  ( TITLE-ABS-KEY ( ( "anti biotic"  OR  antibiotic*  OR  enterobacteria*  OR  "entero bacteria*"  OR  antimicrobial*  OR  microbial*  OR  antibacter*  OR  "anti bacter*"  OR  macrolide*  OR  betalactam*  OR  "beta lactam*" )  W/2  coresist* ) )  OR  ( TITLE-ABS-KEY ( ( "anti biotic"  OR  antibiotic*  OR  enterobacteria*  OR  "entero bacteria*"  OR  antimicrobial*  OR  microbial*  OR  antibacter*  OR  "anti bacter*"  OR  macrolide*  OR  betalactam*  OR  "beta lactam*" )  W/2  select* ) )  OR  ( TITLE-ABS-KEY ( ( "anti biotic"  OR  antibiotic*  OR  enterobacteria*  OR  "entero bacteria*"  OR  antimicrobial*  OR  microbial*  OR  antibacter*  OR  "anti bacter*"  OR  macrolide*  OR  betalactam*  OR  "beta lactam*" )  W/2  resistome* ) )  OR  ( TITLE-ABS-KEY ( ( "anti biotic"  OR  antibiotic*  OR  enterobacteria*  OR  "entero bacteria*"  OR  antimicrobial*  OR  microbial*  OR  antibacter*  OR  "anti bacter*"  OR  macrolide*  OR  betalactam*  OR  "beta lactam*" )  W/2  coselect* ) )  OR  ( TITLE-ABS-KEY ( ( "anti biotic"  OR  antibiotic*  OR  enterobacteria*  OR  "entero bacteria*"  OR  antimicrobial*  OR  microbial*  OR  antibacter*  OR  "anti bacter*"  OR  macrolide*  OR  betalactam*  OR  "beta lactam*" )  W/2  "co select*" ) )  OR  ( TITLE-ABS-KEY ( ( "anti biotic"  OR  antibiotic*  OR  enterobacteria*  OR  "entero bacteria*"  OR  antimicrobial*  OR  microbial*  OR  antibacter*  OR  "anti bacter*"  OR  macrolide*  OR  betalactam*  OR  "beta lactam*" )  W/2  "co resist*" ) )  OR  ( TITLE-ABS-KEY ( ( "anti biotic"  OR  antibiotic*  OR  enterobacteria*  OR  "entero bacteria*"  OR  antimicrobial*  OR  microbial*  OR  antibacter*  OR  "anti bacter*"  OR  macrolide*  OR  betalactam*  OR  "beta lactam*" )  W/2  mobilome* ) ) )  AND  ( TITLE-ABS-KEY ( water*  OR  soil*  OR  wastewater*  OR  air*  OR  effluent*  OR  wetland*  OR  aquatic*  OR  river*  OR  sediment*  OR  lake*  OR  sea*  OR  sewage*  OR  influent*  OR  environment*  OR  natur* ) ) )  AND  ( ( TITLE-ABS-KEY ( "national health service"  OR  nhs ) )  OR  ( TITLE-ABS-KEY ( gb  OR  "g.b."  OR  uk  OR  "u.k."  OR  "northern ireland*"  OR  "nothern irish*"  OR  scotland*  OR  scottish*  OR  welsh ) )  OR  ( TITLE-ABS-KEY ( britain ) )  OR  ( TITLE-ABS-KEY ( ( british  AND not  "british columbia" ) ) )  OR  ( TITLE-ABS-KEY ( "united kingdom*" ) )  OR  ( TITLE-ABS-KEY ( ( ( wales  OR  "south wales" )  not  "new south wales" ) ) )  OR  ( AFFIL ( nhs ) )  OR  ( AFFIL ( nhs ) )  OR  ( AFFIL ( gb ) )  OR  ( AFFIL ( "g.b"  OR  uk ) )  OR  ( AFFIL ( "u.k."  OR  "northern ireland*" ) )  OR  ( AFFIL ( "northern irish*"  OR  scotland*  OR  scottish*  OR  welsh  OR  britain  OR  "united kingdom*" ) )  OR  ( AFFIL ( england ) )  OR  ( AFFIL ( british ) )  OR  ( AFFIL ( wales ) )  OR  ( AFFIL ( "national health service*" ) ) )

| **CEFAS** | **Antimicrobial in title** | **Rowe 2016 (FT downloaded)** | <https://www.cefas.co.uk/data-and-publications/publications/> | **19^th^ May** |
| --- | --- | --- | --- | --- |
| **Env Agency** | **antimicrobial** | **Review on airborne AMR** | <https://www.gov.uk/search/all?keywords=&organisations%5B%5D=environment-agency&public_timestamp%5Bfrom%5D=&public_timestamp%5Bto%5D=> | **19^th^ May** |
| **SEPA** | **antimicrobial** | **Nothing relevant** | <https://www.sepa.org.uk/library/> | **20^th^ May** |
| **DEFRA** |  | **Defra 2006** | <http://sciencesearch.defra.gov.uk/Default.aspx?Location=None&Module=FilterSearchNewLook&Completed=0> | **20^th^ May**  **One project to chase** |
| **APHA** | **Antimicrobial** |  | <https://www.gov.uk/government/organisations/animal-and-plant-health-agency/about/research> | **Had to search each year’s pdf list of publications. 20^th^ May** |
| **HPS** | **Antimicrobial** | **Hps 2019** | <https://www.hps.scot.nhs.uk/publications/> | **20^th^ May. Downloaded one ref** |
| **Welsh Govt** | **Antimicrobial** | **One publication** | <https://gov.wales/publications> | **20^th^ May. Downloaded one ref** |
| **Veterinary Medicines Directory** | **Antimicrobial** | **Nothing found** | <https://www.gov.uk/government/organisations/veterinary-medicines-directorate> | **20^th^ May** |
| **Public Health England** | **Antimicrobial** | **Nothing found** | https://www.gov.uk/government/organisations/public-health-england | **20^th^ May** |
