# Supplementary material for: Existing evidence on antibiotic resistance exposure and transmission to humans from the environment: a systematic map
Source: Environ Evid. 2022 Mar 12;11:8. doi: 10.1186/s13750-022-00262-2 (PMC8917330; doi:10.1186/s13750-022-00262-2)
Supplement: Supplementary file 5 — Additional file 5. Unobtainable articles. [file 13750_2022_262_MOESM5_ESM.docx]

Unobtainable References

**Map 1 (30)**

AALI, R., NIKAEEN, M., KHANAHMAD, H., HEJAZI, Z., KAZEMI, M. & HASSANZADEH, A. 2014. Occurrence of tetracycline resistant bacteria and resistance gene (tetW) in hospital and municipal wastewaters. *Fresenius Environmental Bulletin,* 23**,** 2560-2566.

ADAMS, R. J., MATHYS, D. A., MOLLENKOPF, D. F., WHITTLE, A., DANIELS, J. B. & WITTUM, T. E. 2017. Carbapenemase-producing Aeromonas veronii disseminated in the environment of an equine specialty hospital. *Vector Borne and Zoonotic Diseases,* 17**,** 439-442.

BORGES, C. A., TARLTON, N. J. & RILEY, L. W. 2019. Escherichia coli from Commercial Broiler and Backyard Chickens Share Sequence Types, Antimicrobial Resistance Profiles, and Resistance Genes with Human Extraintestinal Pathogenic Escherichia coli. *Foodborne Pathogens & Disease,* 16**,** 813-822.

ELHAG, W. I., SAEED, H. A. & OMER, E. F. E. 2009. Bacterial etiology and antimicrobials susceptibility of diarrhea among displaced communities during 2006-2008. *Bahrain Medical Bulletin,* 31.

FERNANDES, S. A., CAMARGO, C. H., FRANCISCO, G. R., BUENO, M. F. C., GARCIA, D. O., DOI, Y. & CASAS, M. R. T. 2017. Prevalence of Extended-Spectrum -Lactamases CTX-M-8 and CTX-M-2-Producing Salmonella Serotypes from Clinical and Nonhuman Isolates in Brazil. *Microbial Drug Resistance,* 23**,** 580-589.

GRECU, C., GRECU, A., SERBAN, I. L., HURJUI, I., DELIANU, C., MARANDUCA, M. A., POPOVICI, D., GRADINARU, I., MITREA, M. & HURJUI, L. L. 2018. PREVALENCE OF NASAL CARRIAGE OF STAPHYLOCOCCUS AUREUS WITH SPECIAL REFERENCE TO NUMBER OF METHICILLIN RESISTANCE AND ANTIMICROBIAL EVALUATION AMONG APPARENTLY PEOPLE WITH GOOD HEALTH STATUS. *Medical-Surgical Journal-Revista Medico-Chirurgicala,* 122**,** 819-825.

HASAN, B., LAURELL, K., RAKIB, M. M., AHLSTEDT, E., HERNANDEZ, J., CACERES, M. & JARHULT, J. D. 2016. Fecal Carriage of Extended-Spectrum -Lactamases in Healthy Humans, Poultry, and Wild Birds in Leon, NicaraguaA Shared Pool of bla(CTX-M) Genes and Possible Interspecies Clonal Spread of Extended-Spectrum -Lactamases-Producing Escherichia coli. *Microbial Drug Resistance,* 22**,** 682-687.

HEUDORF, U., VOIGT, K., WESTPHAL, T., STEUL, K., SCHMITHAUSEN, R. & EXNER, M. 2018. Multi-drug resistant pathogens in surface waters - a case history from Frankfurt am Main and its consequences. *Umweltmedizin Hygiene Arbeitsmedizin,* 23**,** 373-379.

JENSEN, L. B., ANGULO, F. J., MOLBAK, K. & WEGENER, H. C. 2009. Human health risks associated with antimicrobial use in animals. *Guide to antimicrobial use in animals*.

JIMÉNEZ-CASTELLANOS, J.-C. A. 2017. *Characterisation and inhibition of antimicrobial drug-resistance in Klebsiella pneumoniae.* [Great Britain] : University of Bristol, 2017.

KARDOS, N. 2015. *Overuse of Antibiotics: Non-medical Applications*, Caister Academic Press, 32 Hewitts Lane, Wymondham Nr 18 0ja, Uk.

KEEN, P. L. & FUGÈRE, R. 2017. *Antimicrobial Resistance in Wastewater Treatment Processes*, wiley.

KEEN, P. L. & MONTFORTS, M. H. M. M. 2011. Antimicrobial resistance in the environment. *Antimicrobial resistance in the environment,* 602.

KIM, M. C., CHA, M. H., RYU, J. G. & WOO, G. J. 2017. Characterization of Vancomycin-Resistant Enterococcus faecalis and Enterococcus faecium Isolated from Fresh Produces and Human Fecal Samples. *Foodborne Pathogens & Disease,* 14**,** 195-201.

KOCK, R., FRITZEMEIER, J., HEINZE, S., HERR, C., HORMANSDORFER, S., KANDLER, U., KUTZORA, S., TEICHERT, U. & WISCHNEWSKI, N. 2019. Occurrence and zoonotic transmission of multidrug-resistant bacteria in Germany. *Umweltmedizin Hygiene Arbeitsmedizin,* 24**,** 71-81.

KUANG, D., ZHANG, J., XU, X., SHI, W., YANG, X., SU, X., SHI, X. & MENG, J. 2018. Increase in Ceftriaxone Resistance and Widespread Extended-Spectrum beta-Lactamases Genes Among Salmonella enterica from Human and Nonhuman Sources. *Foodborne Pathogens & Disease,* 15**,** 770-775.

LOPEZ, M., KADLEC, K., SCHWARZ, S. & TORRES, C. 2012. First detection of the staphylococcal trimethoprim resistance gene dfrK and the dfrK-carrying transposon Tn559 in enterococci. *Microbial Drug Resistance-Mechanisms Epidemiology & Disease,* 18**,** 13-8.

MCLAIN, J. E., ROCK, C. M. & GERBA, C. P. 2017. Environmental Antibiotic Resistance Associated with Land Application of Biosolids. *Antimicrobial Resistance in Wastewater Treatment Processes.* wiley.

MUKHERJEE, N. 2018. *Source Attribution, Antibiotic Resistance and Virulence Properties of <i>Salmonella</i> Serotypes Isolated from Clinically Diagnosed Human Salmonellosis Cases from Tennessee.* 10979152 Ph.D., The University of Memphis.

PARKS, N. 2010. Dishing the dirt on antibiotic resistance. *Frontiers in Ecology & the Environment,* 8**,** 6-6.

SALEH, T. H., SABBAH, M. A., JASEM, K. A. & HAMMAD, Z. N. 2011. Identification of virulence factors in isolated from Iraq during the 2007-2009 outbreak. *Canadian Journal of Microbiology,* 57**,** 1024-1031.

SHI, J., DENG, H. & WANG, M. 2016. Preliminary studies on the pollution levels of antibiotic resistance genes in lower reaches of the Yangtze River. *Journal of Computational and Theoretical Nanoscience,* 13**,** 5971-5974.

SIGNORINI, M. L., ROSSLER, E., DIAZ DAVID, D. C., OLIVERO, C. R., ROMERO-SCHARPEN, A., SOTO, L. P., ASTESANA, D. M., BERISVIL, A. P., ZIMMERMANN, J. A., FUSARI, M. L., FRIZZO, L. S. & ZBRUN, M. V. 2018. Antimicrobial Resistance of Thermotolerant Campylobacter Species Isolated from Humans, Food-Producing Animals, and Products of Animal Origin: A Worldwide Meta-Analysis. *Microbial Drug Resistance-Mechanisms Epidemiology & Disease,* 24**,** 1174-1190.

SIJA, A., MOITRAIYEE, M. & PAROMITA, C. 2017. A review on antibiotics consumption, physico-chemical properties and their sources in Asian soil. (Soil Biology Series). *Antibiotics and antibiotics resistance genes in soils: monitoring, toxicity, risk assessment and management*.

SJOLUND-KARLSSON, M., HOWIE, R. L., BLICKENSTAFF, K., BOERLIN, P., BALL, T., CHALMERS, G., DUVAL, B., HARO, J., RICKERT, R., ZHAO, S., FEDORKA-CRAY, P. J. & WHICHARD, J. M. 2013. Occurrence of beta-lactamase genes among non-Typhi Salmonella enterica isolated from humans, food animals, and retail meats in the United States and Canada. *Microbial Drug Resistance-Mechanisms Epidemiology & Disease,* 19**,** 191-7.

SLIMAN, J. A., EBERLY, B. J., TAMASHIRO, D. A., CALIMLIM, P. S. & WHELEN, A. C. 2008. Community-acquired methicillin-resistant Staphylococcus aureus (CA-MRSA) investigation on a United States Navy ship. *Hawaii Journal of Public Health,* 1**,** 62-67.

WHETSTONE, H. M. 2014. Special Issue: Antibiotic resistance: seeking solutions in a time of growing concern. (Special Issue: Antibiotic resistance: seeking solutions in a time of growing concern.). *Futures,* 32**,** 1-37.

WHITE, D. G. & MCDERMOTT, P. F. 2009. Antimicrobial resistance in food-borne pathogens. *Food borne microbes: shaping the host ecosystem*.

XI, C., BUSH, K., LACHMAYR, K. L., ZHANG, Y. & FORD, T. E. 2009. *INTERACTIONS BETWEEN ENVIRONMENTAL MICROBIAL ECOSYSTEMS AND HUMANS: THE CASE OF THE WATER ENVIRONMENT AND ANTIBIOTIC RESISTANCE*, Amer Soc Microbiology, 1752 N Street Nw, Washington, Dc 20036-2904 USA.

YANG, X., LIU, J., HUANG, Y., MENG, J., LEI, G., JIA, Y., HUANG, W., WANG, Y., ZHANG, L., LV, H. & HE, S. 2018. Prevalence, Molecular Characterization, and Antimicrobial Susceptibility of Methicillin-Resistant Staphylococcus aureus from Different Origins in Sichuan Province, China, 2007-2015. *Foodborne Pathogens & Disease,* 15**,** 705-710.

**Map 2 (6)**

ADAMOU, P., NEUMANN, A. & GRAHAM, D. 2018. Advanced oxidation treatment using clay minerals to remove antibiotic resistance genes in wastewater. *Abstracts of Papers American Chemical Society,* 256**,** 415.

ALHARBI, S. A. 2012. The isolation of MRSA from soils adjacent to hospitals. *Journal of Food Agriculture & Environment,* 10**,** 1100-1102.

BAHO, S. E., HOOSEN, H., SAMARASINGHE, S., WALSH, S., LOBO-BEDMAR, M., DEL AGUILA, C., FENOY, S., IZQUIERDO, F., MAGNET, A. & PENA-FERNANDEZ, A. 2016. Presence of antibiotic-resistant bacteria in faecal samples collected in urban parks in Leicester, UK. *Toxicology Letters (Shannon),* 258**,** S183.

CARSON, M., MEREDITH, A. L., SHAW, D. J., GIOTIS, E. S., LLOYD, D. H. & LOEFFLER, A. 2012. Foxes as a potential wildlife reservoir for mecA-positive Staphylococci. *Vector Borne & Zoonotic Diseases,* 12**,** 583-7.

DESTIANI, R. & TEMPLETON, M. 2016. Antibiotic-resistant bacteria and genes in drinking water. *Abstracts of Papers American Chemical Society,* 252**,** 741.

MCCLUSKEY, S. & KNAPP, C. W. 2017. Selection of tetracycline and ampicillin resistance genes during long-term soil-copper exposure. *Antibiotic Resistance Genes in Natural Environments and Long-Term Effects.* Nova Science Publishers, Inc.
