# Supplementary material for: Existing evidence on antibiotic resistance exposure and transmission to humans from the environment: a systematic map
Source: Environ Evid. 2022 Mar 12;11:8. doi: 10.1186/s13750-022-00262-2 (PMC8917330; doi:10.1186/s13750-022-00262-2)
Supplement: Supplementary file 6 — Additional file 6. Excluded references. [file 13750_2022_262_MOESM6_ESM.docx]

**Excluded references by reason for exclusion**

Map 1.

**1. Abstract or insufficient data (13)**

2009. 4. Resistant E. coli infections in humans come from food animals. *Food & Environment Electronic Digest***,** 4-4.

BADER, O., TUENNERMANN, J., KUHNS, M., REICHARD, U., LUGERT, R., BUCHHEIDT, D., WEIG, M. & GROSS, U. 2013. Correlation of azole drug resistance in Aspergillus fumigatus derived from environmental and clinical sources in Germany. *IJMM International Journal of Medical Microbiology,* 303**,** 100-101.

DALLAL, M. M. S., GHAHREMANI, R., SEPAHI, A. A. & RAJABI, Z. 2019. Frequency, antimicrobial resistance and serotyping of Shigella-contaminated food samples in foodborne disease outbreaks in Iran. *Journal of School of Public Health & Institute of Public Health Research; 2019,* 17**,** Pe110-Pe119.

DANTAS, G. 2015. Networks of Exchanging Antibiotic Resistomes in Human and Environmental Microbiota. *FASEB Journal,* 29**,** 494.1.

DANTAS, G. 2017. Networks of exchanging antibiotic resistance between environmental, commensal, and pathogenic microbes. *FASEB Journal,* 31**,** 404.1.

FALERO-PEREZ, J. M., GARCIA-PETERSON, L. M. & PEREZ-VELEZ, M. E. 2012. Study of the Antibiotic Resistance among Fecal Coliforms Isolated from Freshwaters near Human Health Facilities in the Northeast Area of Puerto Rico. *Abstracts of the General Meeting of the American Society for Microbiology,* 112**,** 1526.

LIU, L., LAN, R., LIU, L., WANG, Y., ZHANG, Y., WANG, Y. & XU, J. 2017. Antimicrobial Resistance and Cytotoxicity of Citrobacter spp. in Maanshan Anhui Province, China. *Frontiers in Microbiology,* 8**,** 1357.

O’FLAHERTY, E., BALCÁZAR, J. L., BORREGO, C. M. & CUMMINS, E. A quantitative human exposure assessment model for antibiotic resistant Escherichia coli through tap water consumption. *In:* POLANSKA, M. E. & VAN IMPE, J. F. M., eds., 2018. EUROSIS, 71-73.

PRUDEN, A., EDWARDS, M., SALVESON, A. & GARNER, E. 2016. Antibiotic resistance and water sustainability: Protecting public health in a changing world. *Abstracts of Papers American Chemical Society,* 251**,** 326.

RANJAN, R. & CHOWDHARY, P. 2017. A rare case of Shewanella putrefaciens bacteremia in a patient of road traffic accident. *Indian Journal of Pathology & Microbiology,* 60**,** 599-600.

STRASSLE, P., THOM, K., JOHNSON, J. K., SHANHOLZ, C., MUELLER, K., LISSAUER, M., REW, C., RAYMOND, L. & HARRIS, A. D. 2011. Environmental Contamination by Patients with Multidrug-Resistant Acinetobacter baumannii. *Abstracts of the Interscience Conference on Antimicrobial Agents and Chemotherapy,* 51**,** K-1465.

TSUKAYAMA, P., PATEL, S., GILMAN, R. H. & DANTAS, G. 2014. Metagenomics Meets Public Health: Transmission Dynamics of Antibiotic Resistance Genes Between Humans and the Environment in a Periurban Slum in Lima, Peru. *Abstracts of the General Meeting of the American Society for Microbiology,* 114**,** 1029.

XIE, J. W., JIN, L., LUO, X. S. & LI, X. D. 2018. Airborne bacteria and antibiotic resistance genes in PM2.5 of Nanjing, China: Spatial-temporal variability and human inhalation risk. *Abstracts of Papers of the American Chemical Society,* 255**,** 1.

**2. Date (2)**

BLAUTH, K. E., SOBSEY, M. D., WILLIAMS, C. M., WORLEY-DAVIS, L. & LIEHR, S. K. Antibiotic resistant bacteria from swine farms compared to row crop farms. 2008. American Society of Agricultural and Biological Engineers, 1-7.

MCLEOD, K. A. 2009. *Microbial contaminated drinking water: A potential reservoir for antibiotic resistant <i>Escherichia coli</i>.* MR51127 M.Sc., University of Calgary (Canada).

**3. Direction (17)**

AFEMA, J. A. 2015. *Salmonella and antimicrobial resistance in humans, livestock and wild birds.* 3715149 Ph.D., Washington State University.

CARROLL, S. P., DAWES, L., HARGREAVES, M. & GOONETILLEKE, A. 2009. Faecal pollution source identification in an urbanising catchment using antibiotic resistance profiling, discriminant analysis and partial least squares regression. *Water Research,* 43**,** 1237-1246.

CARTWRIGHT, E. J., NGUYEN, T., MELLUSO, C., AYERS, T., LANE, C., HODGES, A., LI, X., QUAMMEN, J., YENDELL, S. J., ADAMS, J., MITCHELL, J., RICKERT, R., KLOS, R., WILLIAMS, I. T., BARTON BEHRAVESH, C. & WRIGHT, J. 2016. A Multistate Investigation of Antibiotic-Resistant Salmonella enterica Serotype I 4,[5],12:i:- Infections as Part of an International Outbreak Associated with Frozen Feeder Rodents. *Zoonoses & Public Health,* 63**,** 62-71.

GONÇALVES, V. D., MEIRELLES-PEREIRA, F., CATALDO, M., FONSECA, B. O., NOGUEIRA, B. A., OLIVELLA, J. G. B., ESTEVES, F. A., MATTOS-GUARALDI, A. L., DE ANDRADE, A. F. B., BELLO, A. R. & PEREIRA, J. A. A. 2019. Detection of multidrug-resistant Enterobacteria isolated from river waters flowing to Guanabara Bay (Rio de Janeiro, Brazil) and from clinical samples of hospital origin. *Biomedica,* 39.

HOQUE, R., AHMED, S. M., NAHER, N., ISLAM, M. A., ROUSHAM, E. K., ISLAM, B. Z. & HASSAN, S. 2020. Tackling antimicrobial resistance in Bangladesh: A scoping review of policy and practice in human, animal and environment sectors. *PLoS ONE [Electronic Resource],* 15**,** e0227947.

LEPUSCHITZ, S., BARON, S., LARVOR, E., GRANIER, S. A., PRETZER, C., MACH, R. L., FARNLEITNER, A. H., RUPPITSCH, W., PLEININGER, S., INDRA, A. & KIRSCHNER, A. K. T. 2019. Phenotypic and Genotypic Antimicrobial Resistance Traits of Vibrio cholerae Non-O1/Non-O139 Isolated From a Large Austrian Lake Frequently Associated With Cases of Human Infection. *Frontiers in Microbiology,* 10**,** 2600.

MAHESHWARI, M., YASER, N. H., NAZ, S., FATIMA, M. & AHMAD, I. 2016. Emergence of ciprofloxacin-resistant extended-spectrum beta-lactamase-producing enteric bacteria in hospital wastewater and clinical sources. *Journal of Global Antimicrobial Resistance,* 5**,** 22-5.

MURUGAN, K., PRABHAKARAN, P., AL-SOHAIBANI, S. & SEKAR, K. 2012. Identification of source of faecal pollution of Tirumanimuttar River, Tamilnadu, India using microbial source tracking. *Environmental Monitoring and Assessment,* 184**,** 6001-6012.

MUSIC, M. S., HRENOVIC, J., GOIC-BARISIC, I., HUNJAK, B., SKORIC, D. & IVANKOVIC, T. 2017. Emission of extensively-drug-resistant Acinetobacter baumannii from hospital settings to the natural environment. *Journal of Hospital Infection,* 96**,** 323-327.

NIESTEPSKI, S., HARNISZ, M., KORZENIEWSKA, E., AGUILERA-ARREOLA, M. G., CONTRERAS-RODRIGUEZ, A., FILIPKOWSKA, Z. & OSINSKA, A. 2019. The emergence of antimicrobial resistance in environmental strains of the Bacteroides fragilis group. *Environment International,* 124**,** 408-419.

ORAVCOVA, V., MIHALCIN, M., ZAKOVA, J., POSPISILOVA, L., MASARIKOVA, M. & LITERAK, I. 2017. Vancomycin-resistant enterococci with vanA gene in treated municipal wastewater and their association with human hospital strains. *Science of the Total Environment,* 609**,** 633-643.

ORY, J., BRICHEUX, G., TOGOLA, A., BONNET, J. L., DONNADIEU-BERNARD, F., NAKUSI, L., FORESTIER, C. & TRAORE, O. 2016. Ciprofloxacin residue and antibiotic-resistant biofilm bacteria in hospital effluent. *Environmental Pollution,* 214**,** 635-645.

PESAPANE, R., PONDER, M. & ALEXANDER, K. 2013. Tracking Pathogen Transmission at the Human-Wildlife Interface: Banded Mongoose and Escherichia coli. *EcoHealth,* 10**,** 115-128.

RWEGO, I. B., ISABIRYE-BASUTA, G., GILLESPIE, T. R. & GOLDBERG, T. L. 2008. Gastrointestinal bacterial transmission among humans, mountain gorillas, and livestock in Bwindi Impenetrable National Park, Uganda. *Conservation Biology,* 22**,** 1600-7.

SKURNIK, D., CLERMONT, O., GUILLARD, T., LAUNAY, A., DANILCHANKA, O., PONS, S., DIANCOURT, L., LEBRETON, F., KADLEC, K., ROUX, D., JIANG, D., DION, S., ASCHARD, H., DENAMUR, M., CYWES-BENTLEY, C., SCHWARZ, S., TENAILLON, O., ANDREMONT, A., PICARD, B., MEKALANOS, J., BRISSE, S. & DENAMUR, E. 2016. Emergence of Antimicrobial-Resistant Escherichia coli of Animal Origin Spreading in Humans. *Molecular Biology & Evolution,* 33**,** 898-914.

ZARFEL, G., GALLER, H., FEIERL, G., HAAS, D., KITTINGER, C., LEITNER, E., GRISOLD, A. J., MASCHER, F., POSCH, J., PERTSCHY, B., MARTH, E. & REINTHALER, F. F. 2013. Comparison of extended-spectrum-beta-lactamase (ESBL) carrying Escherichia coli from sewage sludge and human urinary tract infection. *Environmental Pollution,* 173**,** 192-9.

ZHOU, Z. C., FENG, W. Q., HAN, Y., ZHENG, J., CHEN, T., WEI, Y. Y., GILLINGS, M., ZHU, Y. G. & CHEN, H. 2018. Prevalence and transmission of antibiotic resistance and microbiota between humans and water environments. *Environment International,* 121**,** 1155-1161.

**4. Environment (16)**

COLEMAN, B. L. 2008. *The role of drinking water as a source of transmission of antimicrobial resistant <i>Escherichia coli</i>.* NR57973 Ph.D., University of Toronto (Canada).

DOHMEN, W., SCHMITT, H., BONTEN, M. & HEEDERIK, D. 2017. Air exposure as a possible route for ESBL in pig farmers. *Environmental Research,* 155**,** 359-364.

EZZELDEEN, N. A., AL-AMARY, K. F., ABDELMONEM, M. A., EL-MOEZ, S. I. A. & ABDELAZIZ, M. F. 2013. Genotyping characterization of E. coli Egyptian isolates with special reference to drug resistance genes. (The Middle East and North African Journal of Animal Science - Special Issue, no.6, ISSN 2356-6299 (print)). *Proceedings of the 6th Scientific Conference of Animal Wealth Research in the Middle East and North Africa, Hurghada, Egypt*.

FRICKMANN, H., KOLLER, T., HAGEN, R. M., EBERT, K. P., MULLER, M., WENZEL, W., GATZER, R., SCHOTTE, U., BINDER, A., SKUSA, R., WARNKE, P., PODBIELSKI, A., RUCKERT, C. & KREIKEMEYER, B. 2018. Molecular Epidemiology of Multidrug-Resistant Bacteria Isolated from Libyan and Syrian Patients with War Injuries in Two Bundeswehr Hospitals in Germany. *European Journal of Microbiology & Immunology,* 8**,** 1-11.

IWASHITA, Y., ENOKIYA, T., SUZUKI, K., YOKOYAMA, K., YAMAMOTO, A., ISHIKURA, K., OKUDA, M. & IMAI, H. 2013. Arbekacin treatment of a patient infected with a Pseudomonas putida producing a metallo-beta-lactamase. *Journal of intensive care,* 1**,** 3.

LEE, Y. T., FUNG, C. P., WANG, F. D., CHEN, C. P., CHEN, T. L. & CHO, W. L. 2012. Outbreak of imipenem-resistant Acinetobacter calcoaceticus-Acinetobacter baumannii complex harboring different carbapenemase gene-associated genetic structures in an intensive care unit. *Journal of Microbiology, Immunology & Infection,* 45**,** 43-51.

NUANGMEK, A., ROJANASTHIEN, S., CHOTINUN, S., YAMSAKUL, P., TADEE, P., THAMLIKITKUL, V., TANSAKUL, N. & PATCHANEE, P. 2018. Antimicrobial Resistance in ESBL-Producing Escherichia coli Isolated from Layer and Pig Farms in Thailand. *Acta Scientiae Veterinariae,* 46**,** 8.

PILLONETTO, M., MAZZETTI, A., BECKER, G. N., SIEBRA, C. A., AREND, L. N. V. S. & BARTH, A. L. 2019. Low level of polymyxin resistance among nonclonal mcr-1–positive Escherichia coli from human sources in Brazil. *Diagnostic Microbiology & Infectious Disease,* 93**,** 140-142.

PIRS, T., AVBERSEK, J., ZDOVC, I., KRT, B., ANDLOVIC, A., LEJKO-ZUPANC, T., RUPNIK, M. & OCEPEK, M. 2013. Antimicrobial susceptibility of animal and human isolates of Clostridium difficile by broth microdilution. *Journal of Medical Microbiology,* 62**,** 1478-85.

PULSRIKARN, C., SRIYAPAI, P., CHAICHANA, P., NYAMNIYOM, A. & SRIYAPAI, T. 2017. Antimicrobial resistance and molecular characterization of Salmonella enterica serovar Kedougou isolates from clinical specimens and environmental samples in Thailand, 2006-2009. *Southeast Asian Journal of Tropical Medicine and Public Health,* 48**,** 1006-1016.

SIBHAT, B., MOLLA ZEWDE, B., ZERIHUN, A., MUCKLE, A., COLE, L., BOERLIN, P., WILKIE, E., PERETS, A., MISTRY, K. & GEBREYES, W. A. 2011. Salmonella Serovars and Antimicrobial Resistance Profiles in Beef Cattle, Slaughterhouse Personnel and Slaughterhouse Environment in Ethiopia. *Zoonoses and Public Health,* 58**,** 102-109.

SU, X., ZHANG, J., SHI, W., YANG, X., LI, Y., PAN, H., KUANG, D., XU, X., SHI, X. & MENG, J. 2016. Molecular characterization and antimicrobial susceptibility of Listeria monocytogenes isolated from foods and humans. *Food Control,* 70**,** 96-102.

SUELI, A. F., DAVID, L. P., ÂNGELA, C. G.-R., JENNIFER, M. A.-H., ANA, T. T. & YOHEI, D. 2009. CTX-M-2–Producing SalmonellaTyphimurium Isolated from Pediatric Patients and Poultry in Brazil. *Microbial Drug Resistance: Mechanism, Epidemiology, & Disease,* 15**,** 317-321.

TERKURAN, M., ERGINKAYA, Z., GOKMEN, E. U. T., KZLYLDRM, S. & KOKSAL, F. 2014. Comparison of genotypic diversity and vancomycin resistance of enterococci isolated from foods and clinical sources in Adana Region of Turkey. *Kafkas Universitesi Veteriner Fakultesi Dergisi,* 20**,** 117-124.

TOPP, E., SCOTT, A., LAPEN, D. R., LYAUTEY, E. & DURIEZ, P. 2009. Livestock waste treatment systems for reducing environmental exposure to hazardous enteric pathogens: Some considerations. *Bioresource Technology,* 100**,** 5395-5398.

XIE, Y., KANG, M., TAO, C. M., GUO, L., YE, Y. X. & FAN, H. 2010. Molecular Epidemiology of an Outbreak of Multidrug-Resistant Acinetobacter baumannii in an Intensive Care Unit of Victims of the Wenchuan Earthquake. *Labmedicine,* 41**,** 292-295.

**5. Exposure (69)**

ABULREESH, H. H. & ORGANJI, S. R. 2011. The prevalence of multidrug-resistant staphylococci in food and the environment of Makkah, Saudi Arabia. *Research Journal of Microbiology,* 6**,** 510-523.

ACHENBACH, J. E. & BOWEN, R. A. 2013. Effect of oseltamivir carboxylate consumption on emergence of drug-resistant H5N2 avian influenza virus in mallard ducks. *Antimicrobial Agents and Chemotherapy,* 57**,** 2171-2181.

ALIVIDZA, V., MARIANO, V., AHMAD, R., CHARANI, E., RAWSON, T. M., HOLMES, A. H. & CASTRO-SÁNCHEZ, E. 2018. Investigating the impact of poverty on colonization and infection with drug-resistant organisms in humans: a systematic review. *Infectious diseases of poverty,* 7**,** 76.

AL-MASHHADANI, M. N. 2010. *Non-typhoidal salmonellosis : antimicrobial resistance and molecular epidemiology in the U.K.* University of Liverpool, 2010.

ASLANTAS, O., TURKYILMAZ, S., YILMAZ, M. A., ERDEM, Z. & DEMIR, C. 2012. Isolation and molecular characterization of methicillin-resistant staphylococci from horses, personnel and environmental sites at an equine hospital in Turkey. *Journal of Veterinary Medical Science,* 74**,** 1583-8.

AUTHORITY, E. F. S. 2015. ECDC/EFSA/EMA first joint report on the integrated analysis of the consumption of antimicrobial agents and occurrence of antimicrobial resistance in bacteria from humans and food-producing animals. *EFSA Journal,* 13.

BERESIN, G. A., WRIGHT, J. M., RICE, G. E. & JAGAI, J. S. 2017. Swine exposure and methicillin-resistant Staphylococcus aureus infection among hospitalized patients with skin and soft tissue infections in Illinois: A ZIP code-level analysis. *Environmental Research,* 159**,** 46-60.

BUI, D. P., OREN, E., ROE, D. J., BROWN, H. E., HARRIS, R. B., KNIGHT, G. M., GILMAN, R. H. & GRANDJEAN, L. 2019. A Case-Control Study to Identify Community Venues Associated with Genetically-clustered, Multidrug-resistant Tuberculosis Disease in Lima, Peru. *Clinical Infectious Diseases,* 68**,** 1547-1565.

BUSER, G. L., CASSIDY, P. M., PFEIFFER, C. D., TOWNES, J. M., MOREY, K. E., RAYAR, J., KUTUMBAKA, K. K., HAN, S., NADALA, C., SAMADPOUR, M., WEISSMAN, S. J., VEGA, R. & BELDAVS, Z. G. 2017. New Delhi metallo-beta-lactamase-1 (NDM-1) Escherichia coli isolated from household vacuum cleaner-Oregon, 2013. *Idcases,* 9**,** 56-58.

BUSHNELL, G., MITRANI-GOLD, F. & MUNDY, L. M. 2013. Emergence of New Delhi metallo-beta-lactamase type 1-producing enterobacteriaceae and non-enterobacteriaceae: global case detection and bacterial surveillance. *International Journal of Infectious Diseases,* 17**,** e325-33.

CARFORA, V., CAPRIOLI, A., GROSSI, I., PEPE, M., ALBA, P., LORENZETTI, S., AMORUSO, R., SORBARA, L., FRANCO, A. & BATTISTI, A. 2016. A methicillin-resistant Staphylococcus aureus (MRSA) sequence type 8, spa type t11469 causing infection and colonizing horses in Italy. *Pathogens and Disease,* 74.

CARLING, P. C. 2018. Wastewater drains: epidemiology and interventions in 23 carbapenem-resistant organism outbreaks. *Infection Control & Hospital Epidemiology,* 39**,** 972-979.

CHEN, L. F., KNELSON, L. P., GERGEN, M. F., BETTER, O. M., NICHOLSON, B. P., WOODS, C. W., RUTALA, W. A., WEBER, D. J., SEXTON, D. J., ANDERSON, D. J. & PROGRAM, C. D. C. P. E. 2019. A prospective study of transmission of Multidrug-Resistant Organisms (MDROs) between environmental sites and hospitalized patients-the TransFER study. *Infection Control & Hospital Epidemiology,* 40**,** 47-52.

COOKSEY, E. M., SINGH, G., SCOTT, L. C. & AW, T. G. 2019. Detection of coliphages and human adenoviruses in a subtropical estuarine lake. *Science of the Total Environment,* 649**,** 1514-1521.

DAVID, M. Z., GLIKMAN, D., CRAWFORD, S. E., PENG, J., KING, K. J., HOSTETLER, M. A., BOYLE-VAVRA, S. & DAUM, R. S. 2008. What is community-associated methicillin-resistant Staphylococcus aureus? *Journal of Infectious Diseases,* 197**,** 1235-1243.

DAY, M., DOUMITH, M., JENKINS, C., DALLMAN, T. J., HOPKINS, K. L., ELSON, R., GODBOLE, G. & WOODFORD, N. 2017. Antimicrobial resistance in Shiga toxin-producing Escherichia coli serogroups O157 and O26 isolated from human cases of diarrhoeal disease in England, 2015. *Journal of Antimicrobial Chemotherapy,* 72**,** 145-152.

DEBAS, G., KIBRET, M., BIADGLEGNE, F. & ABERA, B. 2011. Prevalence and antimicrobial susceptibility patterns of shigella species at Felege Hiwot Referral Hospital, Northwest Ethiopia. *Ethiopian Medical Journal,* 49**,** 249-56.

DEKSISSA, T. & GEBREMEDHIN, E. Z. 2019. A cross-sectional study of enteric fever among febrile patients at Ambo hospital: prevalence, risk factors, comparison of Widal test and stool culture and antimicrobials susceptibility pattern of isolates. *BMC Infectious Diseases,* 19**,** 288.

DRIEUX, L., HAENN, S., MOULIN, L. & JARLIER, V. 2016. Quantitative evaluation of extended-spectrum beta-lactamase-producing Escherichia coli strains in the wastewater of a French teaching hospital and relation to patient strain. *Antimicrobial Resistance and Infection Control,* 5**,** 9.

EGUALE, T., GEBREYES, W. A., ASRAT, D., ALEMAYEHU, H., GUNN, J. S. & ENGIDAWORK, E. 2015. Non-typhoidal Salmonella serotypes, antimicrobial resistance and co-infection with parasites among patients with diarrhea and other gastrointestinal complaints in Addis Ababa, Ethiopia. *BMC Infectious Diseases,* 15**,** 497.

EREGNO, F. E., TRYLAND, I., TJOMSLAND, T., MYRMEL, M., ROBERTSON, L. & HEISTAD, A. 2016. Quantitative microbial risk assessment combined with hydrodynamic modelling to estimate the public health risk associated with bathing after rainfall events. *Science of the Total Environment,* 548**,** 270-279.

FADLALLAH, S. M., SHEHAB, M., CHEAITO, K., SALEH, M., GHOSN, N., AMMAR, W., EL HAJJ, R. & MATAR, G. M. 2017. Molecular epidemiology and antimicrobial resistance of Salmonella species from clinical specimens and food Items in Lebanon. *Journal of Infection in Developing Countries,* 11**,** 19-27.

FALLAH, A. A., SAEI-DEHKORDI, S. S. & MAHZOUNIEH, M. 2013. Occurrence and antibiotic resistance profiles of Listeria monocytogenes isolated from seafood products and market and processing environments in Iran. *Food Control,* 34**,** 630-636.

FINDLAY, J., HOPKINS, K. L., ALVAREZ-BUYLLA, A., MEUNIER, D., MUSTAFA, N., HILL, R., PIKE, R., MCCRAE, L.-X., HAWKEY, P. M. & WOODFORD, N. 2017. Characterization of carbapenemase-producing Enterobacteriaceae in the West Midlands region of England: 2007-14. *Journal of Antimicrobial Chemotherapy,* 72**,** 1054-1062.

GAO, X., PAN, Q. & HE, M. 2015. Transmission dynamics of resistant bacteria in a predator-prey system. *Computational & Mathematical Methods in Medicine,* 2015**,** 638074.

GLADKIKH, A. S., FERANCHUK, S. I., PONOMAREVA, A. S., BOCHALGIN, N. O. & MIRONOVA, L. V. 2019. Antibiotic resistance in Vibrio cholerae El Tor strains isolated during cholera complications in Siberia and the Far East of Russia. *Infection, Genetics & Evolution,* 78**,** 104096.

GONZALEZ, D., GALLAGHER, E., ZUNIGA, T., LEIVA, J. & VITAS, A. I. 2019. Prevalence and characterization of beta-lactamase-producing Enterobacteriaceae in healthy human carriers. *International Microbiology,* 19**,** 19.

GUK, J. H., KIM, J., SONG, H., KIM, J., AN, J. U., KIM, J., RYU, S., JEON, B. & CHO, S. 2019. Hyper-Aerotolerant Campylobacter coli from Duck Sources and Its Potential Threat to Public Health: Virulence, Antimicrobial Resistance, and Genetic Relatedness. *Microorganisms,* 7**,** 19.

HANNA, N., SUN, P., SUN, Q., LI, X., YANG, X., JI, X., ZOU, H., OTTOSON, J., NILSSON, L. E., BERGLUND, B., DYAR, O. J., TAMHANKAR, A. J. & STALSBY LUNDBORG, C. 2018. Presence of antibiotic residues in various environmental compartments of Shandong province in eastern China: Its potential for resistance development and ecological and human risk. *Environment International,* 114**,** 131-142.

HE, T., WANG, R., LIU, D., WALSH, T. R., ZHANG, R., LV, Y., KE, Y., JI, Q., WEI, R., LIU, Z., SHEN, Y., WANG, G., SUN, L., LEI, L., LV, Z., LI, Y., PANG, M., WANG, L., SUN, Q., FU, Y., SONG, H., HAO, Y., SHEN, Z., WANG, S., CHEN, G., WU, C., SHEN, J. & WANG, Y. 2019. Emergence of plasmid-mediated high-level tigecycline resistance genes in animals and humans. *Nature Microbiology,* 4**,** 1450-1456.

HIBI, A. & KUMANO, Y. 2017. Sphingobacterium spiritivorum bacteremia due to cellulitis in an elderly man with chronic obstructive pulmonary disease and congestive heart failure: a case report. *Journal of Medical Case Reports [Electronic Resource],* 11**,** 277.

KAPPELL, A. D., DENIES, M. S., AHUJA, N. H., LEDEBOER, N. A., NEWTON, R. J. & HRISTOVA, K. R. 2015. Detection of multi-drug resistant Escherichia coli in the urban waterways of Milwaukee, WI. *Frontiers in Microbiology,* 6**,** 336.

KOMBA, E. V. G., MDEGELA, R. H., MSOFFE, P. L. M., NIELSEN, L. N. & INGMER, H. 2015. Prevalence, Antimicrobial Resistance and Risk Factors for Thermophilic Campylobacter Infections in Symptomatic and Asymptomatic Humans in Tanzania. *Zoonoses and Public Health,* 62**,** 557-568.

KOZODEROVIC, G., VELHNER, M., JELESIC, Z., STOJANOV, I., PETROVIC, T., STOJANOVIC, D. & GOLIC, N. 2011. Molecular typing and antimicrobial resistance of Salmonella Enteritidis isolated from poultry, food, and humans in Serbia. *Folia Microbiologica,* 56**,** 66-71.

KRIZMAN, M., AVGUSTIN, J. A., ZDOVC, I., GOLOB, M., TRKOV, M., CIGLENECKI, U. J., BIASIZZO, M. & KIRBIS, A. 2017. Antimicrobial Resistance and Molecular Characterization of Extended-Spectrum β-Lactamases and Other Escherichia coli Isolated from Food of Animal Origin and Human Intestinal Isolates. *Journal of Food Protection,* 80**,** 113-120.

KUSTER, S. P., HASSE, B., HUEBNER, V., BANSAL, V., ZBINDEN, R., RUEF, C., LEDERGERBER, B. & WEBER, R. 2010. Risks factors for infections with extended-spectrum beta-lactamase-producing Escherichia coli and Klebsiella pneumoniae at a tertiary care university hospital in Switzerland. *Infection,* 38**,** 33-40.

LI, B., YANG, X., TAN, H., KE, B., HE, D., WANG, H., CHEN, Q., KE, C. & ZHANG, Y. 2018. Whole genome sequencing analysis of Salmonella enterica serovar Weltevreden isolated from human stool and contaminated food samples collected from the Southern coastal area of China. *International Journal of Food Microbiology,* 266**,** 317-323.

LOCATELLI, C., CREMONESI, P., CAPRIOLI, A., CARFORA, V., IANZANO, A., BARBERIO, A., MORANDI, S., CASULA, A., CASTIGLIONI, B., BRONZO, V. & MORONI, P. 2017. Occurrence of methicillin-resistant Staphylococcus aureus in dairy cattle herds, related swine farms, and humans in contact with herds. *Journal of Dairy Science,* 100**,** 608-619.

LOSASSO, C., DI CESARE, A., MASTRORILLI, E., PATUZZI, I., CIBIN, V., ECKERT, E. M., FONTANETO, D., VANZO, A., RICCI, A. & CORNO, G. 2018. Assessing antimicrobial resistance gene load in vegan, vegetarian and omnivore human gut microbiota. *International Journal of Antimicrobial Agents,* 52**,** 702-705.

MADOSHI, B. P., MTAMBO, M. M. A., MUHAIRWA, A. P., LUPINDU, A. M. & OLSEN, J. E. 2018. Isolation of vancomycin-resistant Enterococcus from apparently healthy human animal attendants, cattle and cattle wastes in Tanzania. *Journal of Applied Microbiology,* 124**,** 1303-1310.

MANENZHE, R. I., ZAR, H. J., NICOL, M. P. & KABA, M. 2015. The spread of carbapenemase-producing bacteria in Africa: a systematic review. *Journal of Antimicrobial Chemotherapy,* 70**,** 23-40.

MARAGKOUDAKIS, S., POULIDAKI, S. R., PAPADOMANOLAKI, E., ALEVRAKI, G., PAPADOGIANNI, M., OIKONOMOU, N. & FANOURGIAKIS, P. 2011. Empiric antimicrobial therapy and infectious diarrhea. Do we need local guidelines? *European Journal of Internal Medicine,* 22**,** e60-2.

MARTINS, M., MCCUSKER, M. P., MCCABE, E. M., O'LEARY, D., DUFFY, G. & FANNING, S. 2013. Evidence of metabolic switching and implications for food safety from the phenome(s) of Salmonella enterica serovar Typhimurium DT104 cultured at selected points across the pork production food chain. *Applied & Environmental Microbiology,* 79**,** 5437-49.

MATAMOROS, S., VAN HATTEM, J. M., ARCILLA, M. S., WILLEMSE, N., MELLES, D. C., PENDERS, J., VINH, T. N., THI HOA, N., CONSORTIUM, C., DE JONG, M. D. & SCHULTSZ, C. 2017. Global phylogenetic analysis of Escherichia coli and plasmids carrying the mcr-1 gene indicates bacterial diversity but plasmid restriction. *Scientific reports,* 7**,** 15364.

MELO, D. B., MENEZES, A. P., REIS, J. N. & GUIMARAES, A. G. 2015. Antimicrobial resistance and genetic diversity of Escherichia coli isolated from humans and foods. *Brazilian Journal of Microbiology,* 46**,** 1165-70.

MELTZER, E., STIENLAUF, S., LESHEM, E., SIDI, Y. & SCHWARTZ, E. 2014. A Large Outbreak of Salmonella Paratyphi A Infection Among Israeli Travelers To Nepal. *Clinical Infectious Diseases,* 58**,** 359-364.

MOMTAZ, F., ALI, M. H., HOSSAIN, M. N., FOYSAL, M. J., SUMIYA, M. K. & ISLAM, K. 2018. Characterisation of Multidrug-resistant Alcaligenes faecalis Strain AF1 Isolated from Patient of RUTIs: A Study from Bangladesh. *Journal of Clinical and Diagnostic Research,* 12**,** KC1-KC4.

MOSHTAGHI, H. 2009. Antimicrobial susceptibility of Listeria monocytogenes isolated from food and clinical cases in Shahrekord, Iran. *Journal of Veterinary Pharmacology and Therapeutics,* 32**,** 211-212.

MULLER, S. 2017. Molecular and functional typing of isolates of the Acinetobacter calcoaceticus-Acinetobacter baumannii complex with emphasis on multi-drug resistant Acinetobacter baumannii. *Molecular and functional typing of isolates of the Acinetobacter calcoaceticus Acinetobacter baumannii complex with emphasis on multi drug resistant Acinetobacter baumannii,* 186.

NADIMPALLI, M. L., DE LAUZANNE, A., PHE, T., BORAND, L., JACOBS, J., FABRE, L., NAAS, T., LE HELLO, S. & STEGGER, M. 2019. Escherichia coli ST410 among humans and the environment in Southeast Asia. *International Journal of Antimicrobial Agents,* 54**,** 228-232.

OSEI SEKYERE, J. & MENSAH, E. 2019. Molecular epidemiology and mechanisms of antibiotic resistance in Enterococcus spp., Staphylococcus spp., and Streptococcus spp. in Africa: a systematic review from a One Health perspective. *Annals of the New York Academy of Sciences*.

OUNDO, J. O., IIJIMA, Y., BOGA, H. I., MULI, F. & KARIUKI, S. 2009. Molecular typing and antibiotic susceptibility patterns of enteropathogenic and shigatoxin producing Escherichia coli isolated from food handlers in three areas of Kenya. *East African Medical Journal,* 86**,** 279-86.

PAINSET, A., DAY, M., DOUMITH, M., RIGBY, J., JENKINS, C., GRANT, K., DALLMAN, T. J., GODBOLE, G. & SWIFT, C. 2020. Comparison of phenotypic and WGS-derived antimicrobial resistance profiles of Campylobacter jejuni and Campylobacter coli isolated from cases of diarrhoeal disease in England and Wales, 2015-16. *Journal of Antimicrobial Chemotherapy,* 16**,** 16.

PAPADOPOULOS, T., PETRIDOU, E., ZDRAGAS, A., MANDILARA, G., VAFEAS, G., PASSIOTOU, M. & VATOPOULOS, A. 2017. Multiple clones and low antimicrobial resistance rates for Salmonella enterica serovar Infantis populations in Greece. *Comparative Immunology, Microbiology & Infectious Diseases,* 51**,** 54-58.

RAMALIVHANA, J. N., OBI, C. L. & MOYO, S. R. 2010. Prevalence of extended-spectrum b-Lactamases producing Aeromonas hydrophila isolated from stool samples collected in the Limpopo province, South Africa. *African Journal of Microbiology Research,* 4**,** 1203-1208.

RAVEN, K. E., LUDDEN, C., GOULIOURIS, T., BLANE, B., NAYDENOVA, P., BROWN, N. M., PARKHILL, J. & PEACOCK, S. J. 2019. Genomic surveillance of Escherichia coli in municipal wastewater treatment plants as an indicator of clinically relevant pathogens and their resistance genes. *Microbial Genomics,* 5**,** 05.

REBELLO, R. C. & REGUA-MANGIA, A. H. 2014. Potential enterovirulence and antimicrobial resistance in Escherichia coli isolates from aquatic environments in Rio de Janeiro, Brazil. *Science of the Total Environment,* 490**,** 19-27.

SABIA, C., DE NIEDERHÄUSERN, S., GUERRIERI, E., MESSI, P., ANACARSO, I., MANICARDI, G. & BONDI, M. 2008. Detection of bacteriocin production and virulence traits in vancomycin-resistant enterococci of different sources. *Journal of Applied Microbiology,* 104**,** 970-979.

SALIMIZAND, H., MENBARI, S., RAMAZANZADEH, R., KHONSHA, M. & VAHEDI, M. S. 2016. DNA fingerprinting and antimicrobial susceptibility pattern of clinical and environmental Acinetobacter baumannii isolates: a multicentre study. *Journal of Chemotherapy,* 28**,** 277-283.

SHIM, S. B., CHUNG, Y. H. & LEE, K. G. 2018. Antimicrobial-resistant Staphylococcus aureus and MRSA prevalence among Korean families and household items. *Food Science and Biotechnology,* 27**,** 269-275.

SHIN, E., HONG, H., PARK, J., OH, Y., JUNG, J. & LEE, Y. 2016. Characterization of Staphylococcus aureus faecal isolates associated with food-borne disease in Korea. *Journal of Applied Microbiology,* 121**,** 277-286.

SILVA, N., IGREJAS, G., GONCALVES, A. & POETA, P. 2012. Commensal gut bacteria: distribution of Enterococcus species and prevalence of Escherichia coli phylogenetic groups in animals and humans in Portugal. *Annals of Microbiology,* 62**,** 449-459.

SUTTERLIN, S., EDQUIST, P., SANDEGREN, L., ADLER, M., TANGDEN, T., DROBNI, M., OLSEN, B. & MELHUS, A. 2014. Silver resistance genes are overrepresented among Escherichia coli isolates with CTX-M production. *Applied & Environmental Microbiology,* 80**,** 6863-9.

VALCEK, A., ROER, L., OVERBALLE-PETERSEN, S., HANSEN, F., BORTOLAIA, V., LEEKITCHAROENPHON, P., KORSGAARD, H. B., SEYFARTH, A. M., HENDRIKSEN, R. S., HASMAN, H. & HAMMERUM, A. M. 2019. IncI1 ST3 and IncI1 ST7 plasmids from CTX-M-1-producing Escherichia coli obtained from patients with bloodstream infections are closely related to plasmids from E. coli of animal origin. *Journal of Antimicrobial Chemotherapy,* 74**,** 2171-2175.

WIELDERS, C. C. H., VAN HOEK, A. H. A. M., HENGEVELD, P. D., VEENMAN, C., DIERIKX, C. M., ZOMER, T. P., SMIT, L. A. M., VAN DER HOEK, W., HEEDERIK, D. J., DE GREEFF, S. C., MAASSEN, C. B. M. & VAN DUIJKEREN, E. 2017. Extended-spectrum β-lactamase- and pAmpC-producing Enterobacteriaceae among the general population in a livestock-dense area. *Clinical Microbiology & Infection,* 23**,** 120.e1-120.e8.

WU, P. C., WANG, J. L., HSUEH, P. R., LIN, P. H., CHENG, M. F., HUANG, I. F., CHEN, Y. S., LEE, S. S., GUANG-YUAN, M., YU, H. C., HSU, C. L., WANG, F. W., CHEN, C. S., HUNG, C. H. & KO, W. C. 2019. Prevalence and risk factors for colonization by extended-spectrum beta-lactamase-producing or ST 131 Escherichia coli among asymptomatic adults in community settings in Southern Taiwan. *Infection & Drug Resistance,* 12**,** 1063-1071.

XU, Z., SHAH, H. N., MISRA, R., CHEN, J., ZHANG, W., LIU, Y., CUTLER, R. R. & MKRTCHYAN, H. V. 2018. The prevalence, antibiotic resistance and mecA characterization of coagulase negative staphylococci recovered from non-healthcare settings in London, UK. *Antimicrobial Resistance & Infection Control,* 7**,** 73.

ZHANG, Q. 2015. *Source and fate of fecal indicator bacteria in tropical soil, sand, and seawater environments.* 3717254 Ph.D., University of Hawai'i at Manoa.

ZURFLUH, K., STEPHAN, R., WIDMER, A., POIREL, L., NORDMANN, P., NUESCH, H. J., HACHLER, H. & NUESCH-INDERBINEN, M. 2017. Screening for fecal carriage of MCR-producing Enterobacteriaceae in healthy humans and primary care patients. *Antimicrobial Resistance & Infection Control,* 6**,** 28.

**6. Geographical/temporal (27)**

BAKER-AUSTIN, C., MCARTHUR, J. V., LINDELL, A. H., WRIGHT, M. S., TUCKFIELD, R. C., GOOCH, J., WARNER, L., OLIVER, J. & STEPANAUSKAS, R. 2009. Multi-site analysis reveals widespread antibiotic resistance in the marine pathogen Vibrio vulnificus. *Microbial Ecology,* 57**,** 151-9.

BEUTLICH, J., JAHN, S., MALORNY, B., HAUSER, E., HUHN, S., SCHROETER, A., RODICIO, M. R., APPEL, B., THRELFALL, J., MEVIUS, D., HELMUTH, R., GUERRA, B. & MED-VET-NET, W. P. P. G. 2011. Antimicrobial resistance and virulence determinants in European Salmonella genomic island 1-positive Salmonella enterica isolates from different origins. *Applied & Environmental Microbiology,* 77**,** 5655-64.

BORJESSON, S., MATUSSEK, A., MELIN, S., LOFGREN, S. & LINDGREN, P. E. 2010. Methicillin-resistant Staphylococcus aureus (MRSA) in municipal wastewater: an uncharted threat? *Journal of Applied Microbiology,* 108**,** 1244-1251.

CABANEL, N., BOUCHIER, C., RAJERISON, M. & CARNIEL, E. 2018. Plasmid-mediated doxycycline resistance in a Yersinia pestis strain isolated from a rat. *International Journal of Antimicrobial Agents,* 51**,** 249-254.

CABANEL, N., GALIMAND, M., BOUCHIER, C., CHESNOKOVA, M., KLIMOV, V. & CARNIEL, E. 2017. Molecular bases for multidrug resistance in Yersinia pseudotuberculosis. *Ijmm International Journal of Medical Microbiology,* 307**,** 371-381.

COLOMER-LLUCH, M., MORA, A., LOPEZ, C., MAMANI, R., DAHBI, G., MARZOA, J., HERRERA, A., VISO, S., BLANCO, J. E., BLANCO, M., ALONSO, M. P., JOFRE, J., MUNIESA, M. & BLANCO, J. 2013. Detection of quinolone-resistant Escherichia coli isolates belonging to clonal groups O25b:H4-B2-ST131 and O25b:H4-D-ST69 in raw sewage and river water in Barcelona, Spain. *Journal of Antimicrobial Chemotherapy,* 68**,** 758-65.

DURSO, L. M., MILLER, D. N. & WIENHOLD, B. J. 2012. Distribution and quantification of antibiotic resistant genes and bacteria across agricultural and non-agricultural metagenomes. *PLoS ONE [Electronic Resource],* 7**,** e48325.

FALGENHAUER, L., IMIRZALIOGLU, C., GHOSH, H., GWOZDZINSKI, K., SCHMIEDEL, J., GENTIL, K., BAUERFEIND, R., KAEMPFER, P., SEIFERT, H., MICHAEL, G. B., SCHWARZ, S., PFEIFER, Y., WERNER, G., PIETSCH, M., ROESLER, U., GUERRA, B., FISCHER, J., SHARP, H., KAESBOHRER, A., GOESMANN, A., HILLE, K., KREIENBROCK, L. & CHAKRABORTY, T. 2016. Circulation of clonal populations of fluoroquinolone-resistant CTX-M-15-producing Escherichia coli ST410 in humans and animals in Germany. *International Journal of Antimicrobial Agents,* 47**,** 457-465.

FAORO, H., OLIVEIRA, W. K., WEISS, V. A., TADRA-SFEIR, M. Z., CARDOSO, R. L., BALSANELLI, E., BRUSAMARELLO-SANTOS, L. C. C., CAMILIOS-NETO, D., CRUZ, L. M., RAITTZ, R. T., MARQUES, A. C. Q., LIPUMA, J., FADEL-PICHETH, C. M. T., SOUZA, E. M. & PEDROSA, F. O. 2019. Genome comparison between clinical and environmental strains of Herbaspirillum seropedicae reveals a potential new emerging bacterium adapted to human hosts. *BMC Genomics,* 20**,** 630.

FREITAS, A. R., NOVAIS, C., RUIZ-GARBAJOSA, P., COQUE, T. M. & PEIXE, L. 2009. Dispersion of multidrug-resistant Enterococcus faecium isolates belonging to major clonal complexes in different Portuguese settings. *Applied & Environmental Microbiology,* 75**,** 4904-8.

GHOLAMI, S., TABATABAEI, M. & SOHRABI, N. 2017. Comparison of biofilm formation and antibiotic resistance pattern of Pseudomonas aeruginosa in human and environmental isolates. *Microbial Pathogenesis,* 109**,** 94-98.

HEIDER, L. C., HOET, A. E., WITTUM, T. E., KHAITSA, M. L., LOVE, B. C., HUSTON, C. L., MORLEY, P. S., FUNK, J. A. & GEBREYES, W. A. 2009. Genetic and phenotypic characterization of the bla(CMY) gene from Escherichia coli and Salmonella enterica isolated from food-producing animals, humans, the environment, and retail meat. *Foodborne Pathogens & Disease,* 6**,** 1235-40.

JALALPOUR, S. 2012. Food borne diseases bacteria; frequency antibiotic resistance bacteria in Iranian foods. African Journal of Microbiology Research, 6, 719-723.

KRUGER, A., LUCCHESI, P. M., SANSO, A. M., ETCHEVERRIA, A. I., BUSTAMANTE, A. V., BURGAN, J., FERNANDEZ, L., FERNANDEZ, D., LEOTTA, G., FRIEDRICH, A. W., PADOLA, N. L. & ROSSEN, J. W. 2015. Genetic characterization of Shiga toxin-producing Escherichia coli O26:H11 strains isolated from animal, food, and clinical samples. *Frontiers in Cellular & Infection Microbiology,* 5**,** 74.

LIRA, F., BERG, G. & MARTINEZ, J. L. 2017. Double-Face Meets the Bacterial World: The Opportunistic Pathogen Stenotrophomonas maltophilia. *Frontiers in Microbiology,* 8**,** 15.

MARIN, M. A., FONSECA, E. L., ANDRADE, B. N., CABRAL, A. C. & VICENTE, A. C. P. 2014. Worldwide Occurrence of Integrative Conjugative Element Encoding Multidrug Resistance Determinants in Epidemic Vibrio cholerae O1. *PLoS One,* 9**,** e108728.

MÜLLER, A., STEPHAN, R. & NÜESCH-INDERBINEN, M. 2016. Distribution of virulence factors in ESBL-producing Escherichia coli isolated from the environment, livestock, food and humans. *Science of the Total Environment,* 541**,** 667-672.

NEGI, M., VERGIS, J., VIJAY, D., DHAKA, P., MALIK, S. V. S., KUMAR, A., POHARKAR, K. V., DOIJAD, S. P., BARBUDDHE, S. B., RAMTEKE, P. W. & RAWOOL, D. B. 2015. Genetic diversity, virulence potential and antimicrobial susceptibility of Listeria monocytogenes recovered from different sources in India. *Pathogens & Disease,* 73**,** 1-8.

NESME, J., CECILLON, S., DELMONT, T. O., MONIER, J. M., VOGEL, T. M. & SIMONET, P. 2014. Large-scale metagenomic-based study of antibiotic resistance in the environment. *Current Biology,* 24**,** 1096-100.

ORAVCOVA, V., PEIXE, L., COQUE, T. M., NOVAIS, C., FRANCIA, M. V., LITERAK, I. & FREITAS, A. R. 2018. Wild corvid birds colonized with vancomycin-resistant Enterococcus faecium of human origin harbor epidemic vanA plasmids. *Environment International,* 118**,** 125-133.

PARK, M. & RAFII, F. 2019. The prevalence of plasmid-coded cpe enterotoxin, beta2 toxin, tpeL toxin, and tetracycline resistance in Clostridium perfringens strains isolated from different sources. *Anaerobe,* 56**,** 124-129.

PRIBUL, B. R., FESTIVO, M. L., RODRIGUES, M. S., COSTA, R. G., RODRIGUES, E. C., DE SOUZA, M. M. & RODRIGUES, D. D. 2017. Characteristics of Quinolone Resistance in Salmonella spp. Isolates from the Food Chain in Brazil. *Frontiers in Microbiology,* 8**,** 299.

RAMSAY, K. A., WARDELL, S. J. T., PATRICK, W. M., BROCKWAY, B., REID, D. W., WINSTANLEY, C., BELL, S. C. & LAMONT, I. L. 2019. Genomic and phenotypic comparison of environmental and patient-derived isolates of Pseudomonas aeruginosa suggest that antimicrobial resistance is rare within the environment. Journal of Medical Microbiology, 68, 1591-1595.

SAHLSTROM, L., REHBINDER, V., ALBIHN, A., ASPAN, A. & BENGTSSON, B. 2009. Vancomycin resistant enterococci (VRE) in Swedish sewage sludge. *Acta Veterinaria Scandinavica,* 51**,** 24.

SCHAUFLER, K., SEMMLER, T., WIELER, L. H., WOHRMANN, M., BADDAM, R., AHMED, N., MULLER, K., KOLA, A., FRUTH, A., EWERS, C. & GUENTHER, S. 2016. Clonal spread and interspecies transmission of clinically relevant ESBL-producing Escherichia coli of ST410--another successful pandemic clone? *FEMS Microbiology Ecology,* 92.

SZMOLKA, A., LIBISCH, B., PASZTI, J., FUZI, M., EMODY, L. & NAGY, B. 2009. Virulence and antimicrobial resistance determinants of human pathogenic and commensal strains of Pseudomonas aeruginosa. *Acta Microbiologica et Immunologica Hungarica,* 56**,** 399-402.

TOBOLDT, A., TIETZE, E., HELMUTH, R., JUNKER, E., FRUTH, A. & MALORNY, B. 2014. Molecular epidemiology of Salmonella enterica serovar Kottbus isolated in Germany from humans, food and animals. *Veterinary Microbiology,* 170**,** 97-108.

VANGCHHIA, B., ABRAHAM, S., BELL, J. M., COLLIGNON, P., GIBSON, J. S., INGRAM, P. R., JOHNSON, J. R., KENNEDY, K., TROTT, D. J., TURNIDGE, J. D. & GORDON, D. M. 2016. Phylogenetic diversity, antimicrobial susceptibility and virulence characteristics of phylogroup F Escherichia coli in Australia. Microbiology, 162, 1904-1912.

YANG, K., WANG, A., FU, M., WANG, A., CHEN, K., JIA, Q. & HUANG, Z. 2020. Investigation of Incidents and Trends of Antimicrobial Resistance in Foodborne Pathogens in Eight Countries from Historical Sample Data. *International Journal of Environmental Research & Public Health [Electronic Resource],* 17**,** 10.

YUKI, S., NAZARENO, P. J., RYUICHI, N., MONDOY, M., AKIYO, N., BUGAYONG, M. P., BILAR, J., V, M. P., JULIAN MEDINA, E., MARIKO, S.-O., MAYUKO, S., KAZUTOSHI, N., HITOSHI, O. & HISAKAZU, Y. 2020. Environmental Presence and Genetic Characteristics of Carbapenemase-Producing Enterobacteriaceae from Hospital Sewage and River Water in the Philippines. *Applied & Environmental Microbiology,* 86**,** 1-10.

ZURFLUH, K., GLIER, M., HAECHLER, H. & STEPHAN, R. 2015. Replicon typing of plasmids carrying bla(CTX-M-15) among Enterobacteriaceae isolated at the environment, livestock and human interface. Science of the Total Environment, 521, 75-78.

**7. Language (1)**

TENHAGEN, B. A., WERNER, N., KASBOHRER, A. & KREIENBROCK, L. 2018. [Transmission pathways for resistant bacteria between animals and humans: antibiotics resistance in the One Health context]. *Bundesgesundheitsblatt, Gesundheitsforschung, Gesundheitsschutz,* 61**,** 515-521.

**8. Link (4)**

ABERA, B., KIBRET, M. & MULU, W. 2016. Extended-Spectrum beta (beta)-Lactamases and Antibiogram in Enterobacteriaceae from Clinical and Drinking Water Sources from Bahir Dar City, Ethiopia. *PLoS ONE [Electronic Resource],* 11**,** e0166519.

BIER, N., SCHWARTZ, K., GUERRA, B. & STRAUCH, E. 2015. Survey on antimicrobial resistance patterns in Vibrio vulnificus and Vibrio cholerae non-O1/non-O139 in Germany reveals carbapenemase-producing Vibrio cholerae in coastal waters. *Frontiers in Microbiology,* 6**,** 1179.

CHIGOR, V. N., UMOH, V. J., SMITH, S. I., IGBINOSA, E. O. & OKOH, A. I. 2010. Multidrug resistance and plasmid patterns of Escherichia coli O157 and other E. coli Isolated from diarrhoeal stools and surface waters from some selected sources in Zaria, Nigeria. *International Journal of Environmental Research & Public Health [Electronic Resource],* 7**,** 3831-41.

PRIETO, M., MARTÍNEZ, C., AGUERRE, L., ROCCA, M. F., CIPOLLA, L. & CALLEJO, R. 2016. Antibiotic susceptibility of Listeria monocytogenes in Argentina. *Enfermedades Infecciosas y Microbiologia Clinica,* 34**,** 91-95.

**9. Location (5)**

BORJESSON, S., NY, S., EGERVARN, M., BERGSTROM, J., ROSENGREN, A., ENGLUND, S., LOFMARK, S. & BYFORS, S. 2016. Limited Dissemination of Extended-Spectrum beta-Lactamase- and Plasmid-Encoded AmpC-Producing Escherichia coli from Food and Farm Animals, Sweden. *Emerging Infectious Diseases,* 22**,** 634-40.

DIONISI, A. M., LUCARELLI, C., BENEDETTI, I., OWCZAREK, S. & LUZZI, I. 2011. Molecular characterisation of multidrug-resistant Salmonella enterica serotype Infantis from humans, animals and the environment in Italy. *International Journal of Antimicrobial Agents,* 38**,** 384-389.

KNIGHT, D. R., KULLIN, B., ANDROGA, G. O., BARBUT, F., ECKERT, C., JOHNSON, S., SPIGAGLIA, P., TATEDA, K., TSAI, P. J. & RILEY, T. V. 2019. Evolutionary and Genomic Insights into Clostridioides difficile Sequence Type 11: a Diverse Zoonotic and Antimicrobial-Resistant Lineage of Global One Health Importance. *mBio,* 10**,** 16.

MOURAO, J., MACHADO, J., NOVAIS, C., ANTUNES, P. & PEIXE, L. 2014. Characterization of the emerging clinically-relevant multidrug-resistant Salmonella enterica serotype 4,[5],12:i:- (monophasic variant of S. Typhimurium) clones. *European Journal of Clinical Microbiology & Infectious Diseases,* 33**,** 2249-57.

MOURKAS, E., FLOREZ-CUADRADO, D., PASCOE, B., CALLAND, J. K., BAYLISS, S. C., MAGEIROS, L., MERIC, G., HITCHINGS, M. D., QUESADA, A., PORRERO, C., UGARTE-RUIZ, M., GUTIERREZ-FERNANDEZ, J., DOMINGUEZ, L. & SHEPPARD, S. K. 2019. Gene pool transmission of multidrug resistance among Campylobacter from livestock, sewage and human disease. *Environmental Microbiology,* 21**,** 4597-4613.

**10. No human measurement (10)**

BONARDI, S. & PITINO, R. 2019. Carbapenemase-producing bacteria in food-producing animals, wildlife and environment: A challenge for human health. *Italian Journal of Food Safety,* 8**,** 7956.

DE ROOIJ, M. M. T., HOEK, G., SCHMITT, H., JANSE, I., SWART, A., MAASSEN, C. B. M., SCHALK, M., HEEDERIK, D. J. J. & WOUTERS, I. M. 2019. Insights into Livestock-Related Microbial Concentrations in Air at Residential Level in a Livestock Dense Area. *Environmental Science & Technology,* 53**,** 7746-7758.

EGUALE, T., MARSHALL, J., MOLLA, B., BHATIYA, A., GEBREYES, W. A., ENGIDAWORK, E., ASRAT, D. & GUNN, J. S. 2014. Association of multicellular behaviour and drug resistance in Salmonella enterica serovars isolated from animals and humans in Ethiopia. *Journal of Applied Microbiology,* 117**,** 961-971.

HALDER, M., MOOKERJEE, S., BATABYAL, P. & PALIT, A. 2018. Waterborne outbreaks in diarrhoea endemic foci of India: a longitudinal exploration and its implications. *Environmental Monitoring & Assessment,* 190**,** 0-0.

HELLEIN, K. N., BATTIE, C., TAUCHMAN, E., LUND, D., OYARZABAL, O. A. & LEPO, J. E. 2011. Culture-based indicators of fecal contamination and molecular microbial indicators rarely correlate with Campylobacter spp. in recreational waters. *Journal of Water and Health,* 9**,** 695-707.

JAJA, I. F., BHEMBE, N. L., GREEN, E., OGUTTU, J. & MUCHENJE, V. 2019. Molecular characterisation of antibiotic-resistant Salmonella enterica isolates recovered from meat in South Africa. *Acta Tropica,* 190**,** 129-136.

SHOST, S. J. 2010. *Multiple antibiotic resistant <i>Staphylococcus</i> spp. in environmental settings.* 3396967 Ph.D., State University of New York at Albany.

SIGALA, J. & UNC, A. 2012. A PCR-DGGE approach to evaluate the impact of wastewater source on the antibiotic resistance diversity in treated wastewater effluent. *Water Science and Technology,* 65**,** 1323-1331.

STEELE, M., SHAZALI, S. A., CUTLER, R. R. & IDRIS, A. 2017. High prevalence of multiple drug resistant staphylococci observed in macaque-populated locations in Brunei Darussalam. *Tropical Biomedicine,* 34**,** 32-36.

YUAN, W., TIAN, T., YANG, Q. & RIAZ, L. 2019. Transfer potentials of antibiotic resistance genes in Escherichia spp. strains from different sources. *Chemosphere,* 246**,** 125736.

**11. Outcome (21)**

ABDELRAHIM, K. A. A., HASSANEIN, A. M. & ABD EL AZEIZ, H. A. E. H. 2015. Prevalence, Plasmids and Antibiotic Resistance Correlation of Enteric Bacteria in Different Drinking Water Resources in Sohag, Egypt. *Jundishapur Journal of Microbiology,* 8**,** e18648.

ABDOLMALEKI, Z., MASHAK, Z. & SAFARPOOR DEHKORDI, F. 2019. Phenotypic and genotypic characterization of antibiotic resistance in the methicillin-resistant Staphylococcus aureus strains isolated from hospital cockroaches. *Antimicrobial Resistance & Infection Control,* 8**,** 54.

ADENIJI, O. O., SIBANDA, T. & OKOH, A. I. 2019. Recreational water quality status of the Kidd's Beach as determined by its physicochemical and bacteriological quality parameters. *Heliyon,* 5**,** 7.

ADENODI, S. A., OYEJIDE, N. E., FAYEMI, S. O. & AYOADE, F. 2014. Prevalence of antibiotic-resistant strains of Escherichia coli in drinking water samples from Mowe Metropolis, Ogun State, Nigeria. *African Journal of Clinical and Experimental Microbiology,* 15**,** 69-75.

AGGA, G. E., ARTHUR, T. M., DURSO, L. M., HARHAY, D. M. & SCHMIDT, J. W. 2015. Antimicrobial-Resistant Bacterial Populations and Antimicrobial Resistance Genes Obtained from Environments Impacted by Livestock and Municipal Waste. *PLoS One,* 10**,** e0132586.

AGUILERA, R., GERSHUNOV, A. & BENMARHNIA, T. 2019. Atmospheric rivers impact California's coastal water quality via extreme precipitation. *Science of the Total Environment,* 671**,** 488-494.

AHAMMAD, Z. S., SREEKRISHNAN, T. R., HANDS, C. L., KNAPP, C. W. & GRAHAM, D. W. 2014. Increased Waterborne blaNDM-1 Resistance Gene Abundances Associated with Seasonal Human Pilgrimages to the Upper Ganges River. *Environmental Science & Technology,* 48**,** 3014-3020.

ALEMU, A., GETA, M., TAYE, S., ESHETIE, S. & ENGDA, T. 2019. Prevalence, associated risk factors and antimicrobial susceptibility patterns of Shigella infections among diarrheic pediatric population attending at Gondar town healthcare institutions, Northwest Ethiopia. *Tropical Diseases Travel Medicine & Vaccines,* 5**,** 7.

ARSLAN, S. & KUCUKSARI, R. 2015. Phenotypic and Genotypic Virulence Factors and Antimicrobial Resistance of Motile Aeromonas spp. from Fish and Ground Beef. *Journal of Food Safety,* 35**,** 551-559.

BENAVIDES, J. A., GODREUIL, S., BODENHAM, R., RATIARISON, S., DEVOS, C., PETRETTO, M.-O., RAYMOND, M. & ESCOBAR-PÁRAMO, P. 2012. No Evidence for Transmission of Antibiotic-Resistant Escherichia coli Strains from Humans to Wild Western Lowland Gorillas in Lopé National Park, Gabon. *Applied & Environmental Microbiology,* 78**,** 4281-4287.

BUBLITZ, D. C., WRIGHT, P. C., BODAGER, J. R., RASAMBAINARIVO, F. T., BLISKA, J. B. & GILLESPIE, T. R. 2014. Epidemiology of Pathogenic Enterobacteria in Humans, Livestock, and Peridomestic Rodents in Rural Madagascar. *Plos One,* 9**,** 10.

CHAUDHRY, T. H., ASLAM, B., ARSHAD, M. I., NAWAZ, Z. & WASEEM, M. 2019. Occurrence of ESBL-producing Klebsiella pneumoniae in hospital settings and waste. *Pakistan Journal of Pharmaceutical Sciences,* 32**,** 773-778.

CHEN, H., BAI, X., JING, L., CHEN, R. & TENG, Y. 2019. Characterization of antibiotic resistance genes in the sediments of an urban river revealed by comparative metagenomics analysis. *Science of the Total Environment,* 653**,** 1513-1521.

COERTZE, R. D. & BEZUIDENHOUT, C. C. 2019. Global distribution and current research of AmpC beta-lactamase genes in aquatic environments: A systematic review. *Environmental Pollution,* 252**,** 1633-1642.

HASAN, B., MELHUS, A., SANDEGREN, L., MUNIRUL, A. & OLSEN, B. 2014. The gull (Chroicocephalus brunnicephalus) as an environmental bioindicator and reservoir for antibiotic resistance on the coastlines of the Bay of Bengal. *Microbial Drug Resistance,* 20**,** 466-471.

KOSEK, M., YORI, P. P., PAN, W. K., OLORTEGUI, M. P., GILMAN, R. H., PEREZ, J., CHAVEZ, C. B., SANCHEZ, G. M., BURGA, R. & HALL, E. 2008. Epidemiology of highly endemic multiply antibiotic-resistant shigellosis in children in the Peruvian Amazon. *Pediatrics,* 122**,** e541-9.

LI, F., WANG, W., ZHU, Z., CHEN, A., DU, P., WANG, R., CHEN, H., HU, Y., LI, J., KAN, B. & WANG, D. 2015. Distribution, virulence-associated genes and antimicrobial resistance of Aeromonas isolates from diarrheal patients and water, China. *Journal of Infection,* 70**,** 600-8.

LUPINDU, A. M., OLSEN, J. E., NGOWI, H. A., MSOFFE, P. L., MTAMBO, M. M., SCHEUTZ, F. & DALSGAARD, A. 2014. Occurrence and characterization of Shiga toxin-producing Escherichia coli O157:H7 and other non-sorbitol-fermenting E. coli in cattle and humans in urban areas of Morogoro, Tanzania. *Vector Borne & Zoonotic Diseases,* 14**,** 503-10.

OLIVEIRA, K. W. D., GOMES, F. D. C. O., BENKO, G., PIMENTA, R. S., MAGALHAES, P. P., MENDES, E. N. & MORAIS, P. B. D. 2012. Antimicrobial resistance profiles of diarrheagenic Escherichia coli strains isolated from bathing waters of the Lajeado reservoir in Tocantins, Brazil. *Ambiente & Agua,* 7**,** 30-41.

PLANO, L., SHIBATA, T., GARZA, A., KISH, J., FLEISHER, J., SINIGALLIANO, C., GIDLEY, M., WITHUM, K., ELMIR, S., HOWER, S., JACKSON, C., BARRETT, J., CLEARY, T., DAVIDSON, M., DAVIS, J., MUKHERJEE, S., FLEMING, L. & SOLO-GABRIELE, H. 2013. Human-Associated Methicillin-Resistant Staphylococcus aureus from a Subtropical Recreational Marine Beach. Microbial Ecology, 65, 1039-1051.

TICHENOR, W. S., THURLOW, J., MCNULTY, S., BROWN-ELLIOTT, B. A., WALLACE JR, R. J. & FALKINHAM III, J. O. 2012. Nontuberculous Mycobacteria in Household Plumbing as Possible Cause of Chronic Rhinosinusitis. *Emerging Infectious Diseases,* 18**,** 1612-1617.

WALTERS, M. S., ROUTH, J., MIKOLEIT, M., KADIVANE, S., OUMA, C., MUBIRU, D., MBUSA, B., MURANGI, A., EJOKU, E., RWANTANGLE, A., KULE, U., LULE, J., GARRETT, N., HALPIN, J., MAXWELL, N., KAGIRITA, A., MULABYA, F., MAKUMBI, I., FREEMAN, M., JOYCE, K., HILL, V., DOWNING, R. & MINTZ, E. 2014. Shifts in Geographic Distribution and Antimicrobial Resistance during a Prolonged Typhoid Fever Outbreak - Bundibugyo and Kasese Districts, Uganda, 2009-2011. *PloS Neglected Tropical Diseases,* 8**,** e2726.

**12. Population (15)**

AALI, R., NIKAEEN, M., KHANAHMAD, H. & HASSANZADEH, A. 2014. Monitoring and comparison of antibiotic resistant bacteria and their resistance genes in municipal and hospital wastewaters. *International Journal of Preventive Medicine,* 5**,** 887-94.

ABHIROSH, C., SHERIN, V., THOMAS, A. P., HATHA, A. A. & MAZUMDER, A. 2011. Potential public health significance of faecal contamination and multidrug-resistant Escherichia coli and Salmonella serotypes in a lake in India. *Public Health,* 125**,** 377-9.

ACHUDUME, A. C. & OLAWALE, J. T. 2009. Occurrence of antibiotic resistant bacteria in waste site of Ede south west Nigeria. *Journal of Environmental Biology,* 30**,** 187-9.

ADAMS, R. J., KIM, S. S., MOLLENKOPF, D. F., MATHYS, D. A., SCHUENEMANN, G. M., DANIELS, J. B. & WITTUM, T. E. 2018. Antimicrobial-resistant Enterobacteriaceae recovered from companion animal and livestock environments. *Zoonoses & Public Health,* 65**,** 519-527.

AFEMA, J. A., BYARUGABA, D. K., SHAH, D. H., ATUKWASE, E., NAMBI, M. & SISCHO, W. M. 2016. Potential Sources and Transmission of Salmonella and Antimicrobial Resistance in Kampala, Uganda. *PLoS One,* 11**,** e0152130.

BORGES, C. A., BERALDO, L. G., MALUTA, R. P., CARDOZO, M. V., BARBOZA, K. B., GUASTALLI, E. A. L., KARIYAWASAM, S., DEBROY, C. & AVILA, F. A. 2017. Multidrug-resistant pathogenic Escherichia coli isolated from wild birds in a veterinary hospital. *Avian Pathology,* 46**,** 76-83.

CHEN, J., MCILROY, S. E., ARCHANA, A., BAKER, D. M. & PANAGIOTOU, G. 2019. A pollution gradient contributes to the taxonomic, functional, and resistome diversity of microbial communities in marine sediments. *Microbiome,* 7**,** 104.

DUARTE, A., SANTOS, A., MANAGEIRO, V., MARTINS, A., FRAQUEZA, M. J., CANIÇA, M., DOMINGUES, F. C. & OLEASTRO, M. 2014. Human, food and animal Campylobacter spp. isolated in Portugal: High genetic diversity and antibiotic resistance rates. *International Journal of Antimicrobial Agents,* 44**,** 306-313.

FRESIA, P., ANTELO, V., SALAZAR, C., GIMENEZ, M., D'ALESSANDRO, B., AFSHINNEKOO, E., MASON, C., GONNET, G. H. & IRAOLA, G. 2019. Urban metagenomics uncover antibiotic resistance reservoirs in coastal beach and sewage waters. *Microbiome,* 7**,** 35.

GARCÍA-COBOS, S., KÖCK, R., MELLMANN, A., FRENZEL, J., FRIEDRICH, A. W. & ROSSEN, J. W. A. 2015. Molecular typing of Enterobacteriaceae from pig holdings in North-Western Germany reveals extended- spectrum and AmpC β-lactamases producing but no carbapenem resistant ones. *PLoS ONE,* 10.

GUENTHER, S., EWERS, C. & WIELER, L. H. 2011. Extended-Spectrum Beta-Lactamases Producing E. coli in Wildlife, yet Another Form of Environmental Pollution? *Frontiers in Microbiology,* 2**,** 246.

LONCARIC, I., KUNZEL, F., KLANG, A., WAGNER, R., LICKA, T., GRUNERT, T., FESLER, A. T., GEIER-DOMLING, D., ROSENGARTEN, R., MULLER, E., REISSIG, A., SPERGSER, J., SCHWARZ, S., EHRICHT, R. & MONECKE, S. 2016. Carriage of meticillin-resistant staphylococci between humans and animals on a small farm. *Veterinary Dermatology,* 27**,** 191-e48.

MEIR-GRUBER, L., MANOR, Y., GEFEN-HALEVI, S., HINDIYEH, M. Y., MILEGUIR, F., AZAR, R., SMOLLAN, G., BELAUSOV, N., RAHAV, G., SHAMISS, A., MENDELSON, E. & KELLER, N. 2016. Population Screening Using Sewage Reveals Pan-Resistant Bacteria in Hospital and Community Samples. *PLoS One,* 11**,** e0164873.

MIRANDA, C. C., DE FILIPPIS, I., PINTO, L. H., COELHO-SOUZA, T., BIANCO, K., CACCI, L. C., PICAO, R. C. & CLEMENTINO, M. M. 2015. Genotypic characteristics of multidrug-resistant Pseudomonas aeruginosa from hospital wastewater treatment plant in Rio de Janeiro, Brazil. *Journal of Applied Microbiology,* 118**,** 1276-1286.

RAHMAN, M., HUYS, G., KUHN, I., RAHMAN, M. & MOLLBY, R. 2009. Prevalence and transmission of antimicrobial resistance among Aeromonas populations from a duckweed aquaculture based hospital sewage water recycling system in Bangladesh. *Antonie van Leeuwenhoek,* 96**,** 313-321.

**13. Publication/study type (33)**

2008. The growing burden of antimicrobial resistance. *Journal of Antimicrobial Chemotherapy (JAC),* 62**,** i1-i1.

2011. Special Issue: Antimicrobial resistance in South-East Asia. (Special Issue: Antimicrobial resistance in South-East Asia.). *Regional Health Forum,* 15**,** 1-137.

2014. Fate of Viruses in Water Systems. *Journal of Environmental Engineering,* 140**,** -1.

ADAMS, V., HAN, X., LYRAS, D. & ROOD, J. I. 2018. Antibiotic resistance plasmids and mobile genetic elements of Clostridium perfringens. *Plasmid,* 99**,** 32-39.

AWANISH, K. & DHARM, P. 2018. Antibiotic resistance and wastewater: correlation, impact and critical human health challenges. *Journal of Environmental Chemical Engineering,* 6**,** 52-58.

BOULANT, T., BOUDEHENR, Y. M., FILLOUX, A., PLESIAT, P., NAAS, T. & DORTET, L. 2018. Higher Prevalence of PIdA, Pseudomonas aeruginosa Trans-Kingdom H2-Type VI Secretion System Effector, in Clinical Isolates Responsible for Acute Infections and in Multidrug Resistant Strains. *Frontiers in Microbiology,* 9**,** 7.

DIAZ, J. H. & LOPEZ, F. A. 2015. Skin, Soft Tissue and Systemic Bacterial Infections Following Aquatic Injuries and Exposures. *American Journal of the Medical Sciences,* 349**,** 269-275.

ESCUDEIRO, P., POTHIER, J., DIONISIO, F. & NOGUEIRA, T. 2019. Antibiotic Resistance Gene Diversity and Virulence Gene Diversity Are Correlated in Human Gut and Environmental Microbiomes. *Msphere,* 4**,** 01.

FOKA, F. E. T., AJAY, K. & ATEBA, C. N. 2018. Emergence of vancomycin-resistant enterococci in South Africa: implications for public health. *South African Journal of Science,* 114**,** 20-26.

GEORGE, E. A., SANKAR, S., JESUDASAN, M. V., SUDANDIRADOSS, C. & NANDAGOPAL, B. 2014. Incidence of extended spectrum beta lactamase producing Escherichia coli among patients, healthy individuals and in the environment. *Indian Journal of Medical Microbiology,* 32**,** 172-4.

GEORGE, F., DANIEL, C., THOMAS, M., SINGER, E., GUILBAUD, A., TESSIER, F. J., REVOL-JUNELLES, A. M., BORGES, F. & FOLIGNÉ, B. 2018. Occurrence and dynamism of lactic acid bacteria in distinct ecological niches: A multifaceted functional health perspective. *Frontiers in Microbiology,* 9.

GUERRA, B., FISCHER, J. & HELMUTH, R. 2014. An emerging public health problem: acquired carbapenemase-producing microorganisms are present in food-producing animals, their environment, companion animals and wild birds. *Veterinary Microbiology,* 171**,** 290-7.

GWENZI, W., MUSIYIWA, K. & MANGORI, L. 2018. Sources, behaviour and health risks of antimicrobial resistance genes in wastewaters: A hotspot reservoir. *Journal of Environmental Chemical Engineering*.

HAMMERUM, A. M. 2012. Enterococci of animal origin and their significance for public health. *Clinical Microbiology & Infection,* 18**,** 619-625.

HARRIS, S., CORMICAN, M. & CUMMINS, E. 2012. Antimicrobial residues and antimicrobial-resistant bacteria: impact on the microbial environment and risk to human health - a review. *Human and Ecological Risk Assessment,* 18**,** 767-809.

HOELZER, K., WONG, N., THOMAS, J., TALKINGTON, K., JUNGMAN, E. & COUKELL, A. 2017. Antimicrobial drug use in food-producing animals and associated human health risks: what, and how strong, is the evidence? *BMC Veterinary Research [Electronic Resource],* 13**,** 211.

KARIUKI, S., MBAE, C., ONSARE, R., KAVAI, S. M., WAIRIMU, C., NGETICH, R., ALI, M., CLEMENS, J. & DOUGAN, G. 2019. Multidrug-resistant Nontyphoidal Salmonella Hotspots as Targets for Vaccine Use in Management of Infections in Endemic Settings. *Clinical Infectious Diseases,* 68**,** S10-S15.

KHADEMI, F. & SAHEBKAR, A. 2019. A systematic review and meta-analysis on the prevalence of antibiotic-resistant Listeria species in food, animal and human specimens in Iran. *Journal of food science and technology,* 56**,** 5167-5183.

LEBRETON, F., VAN SCHAIK, W., MCGUIRE, A. M., GODFREY, P., GRIGGS, A., MAZUMDAR, V., CORANDER, J., CHENG, L., SAIF, S., YOUNG, S., ZENG, Q., WORTMAN, J., BIRREN, B., WILLEMS, R. J., EARL, A. M. & GILMORE, M. S. 2013. Emergence of epidemic multidrug-resistant Enterococcus faecium from animal and commensal strains. *mBio,* 4**,** 20.

MARINHO, C. M., SANTOS, T., GONCALVES, A., POETA, P. & IGREJAS, G. 2016. A Decade-Long Commitment to Antimicrobial Resistance Surveillance in Portugal. *Frontiers in Microbiology,* 7**,** 1650.

MAY, M. 2018. The Bacteria on Your Beaches: ARE MORE ANTIBIOTIC-RESISTANT BACTERIA GETTING INTO THE OCEAN? *Oceanus,* 53**,** 24-27.

NORDMANN, P. & POIREL, L. 2016. Plasmid-mediated colistin resistance: an additional antibiotic resistance menace. *Clinical Microbiology & Infection,* 22**,** 398-400.

PARK, K., MOK, J., KWON, J., RYU, A., KIM, S. & LEE, H. 2018. Food-borne outbreaks, distributions, virulence, and antibiotic resistance profiles of Vibrio parahaemolyticus in Korea from 2003 to 2016: a review. *Fisheries and Aquatic Sciences,* 21.

PEPPER, I. L., BROOKS, J. P. & GERBA, C. P. 2018. Antibiotic Resistant Bacteria in Municipal Wastes: Is There Reason for Concern? *Environmental Science & Technology,* 52**,** 3949-3959.

PETERSON, E. & KAUR, P. 2018. Antibiotic Resistance Mechanisms in Bacteria: Relationships Between Resistance Determinants of Antibiotic Producers, Environmental Bacteria, and Clinical Pathogens. *Frontiers in Microbiology,* 9**,** 2928.

QAMAR, F. N., YOUSAFZAI, M. T., KHALID, M., KAZI, A. M., LOHANA, H., KARIM, S., KHAN, A., HOTWANI, A., QURESHI, S., KABIR, F., AZIZ, F., MEMON, N. M., DOMKI, M. H. & HASAN, R. 2018. Outbreak investigation of ceftriaxone-resistant Salmonella enterica serotype Typhi and its risk factors among the general population in Hyderabad, Pakistan: a matched case-control study. *The Lancet Infectious Diseases,* 18**,** 1368-1376.

SANGANYADO, E. & GWENZI, W. 2019. Antibiotic resistance in drinking water systems: Occurrence, removal, and human health risks. *Science of the Total Environment,* 669**,** 785-797.

VITTECOQ, M., GODREUIL, S., PRUGNOLLE, F., DURAND, P., BRAZIER, L., RENAUD, N., ARNAL, A., ABERKANE, S., JEAN-PIERRE, H., GAUTHIER-CLERC, M., THOMAS, F. & RENAUD, F. 2016. Antimicrobial resistance in wildlife. *Journal of Applied Ecology,* 53**,** 519-529.

WALSER, S. M., GERSTNER, D. G., BRENNER, B., BÜNGER, J., EIKMANN, T., JANSSEN, B., KOLB, S., KOLK, A., NOWAK, D., RAULF, M., SAGUNSKI, H., SEDLMAIER, N., SUCHENWIRTH, R., WIESMÜLLER, G., WOLLIN, K. M., TESSERAUX, I. & HERR, C. E. 2015. Evaluation of exposure-response relationships for health effects of microbial bioaerosols - A systematic review. *International journal of hygiene and environmental health,* 218**,** 577-89.

WHICHARD, J. M., GAY, K., TATE, H. & CHILLER, T. M. 2013. Surveillance for foodborne diseases: PART 3: Surveillance for antimicrobial resistance among foodborne bacteria-the US approach. *Infectious Disease Surveillance: Second Edition.* John Wiley and Sons.

YOUNG, N. 2016. The association between marine bathing and infectious diseases-- a review. *Journal of the Marine Biological Association of the United Kingdom,* 96**,** 93-100.

YUEH, M.-F. & TUKEY, R. H. 2016. Triclosan: A Widespread Environmental Toxicant with Many Biological Effects. *Annual Review of Pharmacology & Toxicology,* 56**,** 251-272.

ZHANG, X. X., ZHANG, T. & FANG, H. H. 2009. Antibiotic resistance genes in water environment. *Applied Microbiology & Biotechnology,* 82**,** 397-414.

Map 2.

Excluded references by reason for exclusion

1. **Combined water types (1)**

YANG, Y., LI, Z., SONG, W., DU, L., YE, C., ZHAO, B., LIU, W., DENG, D., PAN, Y., LIN, H. & CAO, X. 2019. Metagenomic insights into the abundance and composition of resistance genes in aquatic environments: Influence of stratification and geography. *Environment International,* 127**,** 371-380.

1. **Exposure source (14)**

BOIOCCHI, F., DAVIES, M. P. & HILTON, A. C. 2019. An Examination of Flying Insects in Seven Hospitals in the United Kingdom and Carriage of Bacteria by True Flies (Diptera: Calliphoridae, Dolichopodidae, Fanniidae, Muscidae, Phoridae, Psychodidae, Sphaeroceridae). *Journal of Medical Entomology,* 21**,** 21.

BREATHNACH, A. S., CUBBON, M. D., KARUNAHARAN, R. N., POPE, C. F. & PLANCHE, T. D. 2012. Multidrug-resistant Pseudomonas aeruginosa outbreaks in two hospitals: association with contaminated hospital waste-water systems. *Journal of Hospital Infection,* 82**,** 19-24.

DECRAENE, V., PHAN, H. T. T., GEORGE, R., WYLLIE, D. H., AKINREMI, O., AIKEN, Z., CLEARY, P., DODGSON, A., PANKHURST, L., CROOK, D. W., LENNEY, C., WALKER, A. S., WOODFORD, N., SEBRA, R., FATH-ORDOUBADI, F., MATHERS, A. J., SEALE, A. C., GUIVER, M., MCEWAN, A., WATTS, V., WELFARE, W., STOESSER, N., CAWTHORNE, J. & GROUP, T. I. 2018. A Large, Refractory Nosocomial Outbreak of Klebsiella pneumoniae Carbapenemase-Producing Escherichia coli Demonstrates Carbapenemase Gene Outbreaks Involving Sink Sites Require Novel Approaches to Infection Control. *Antimicrobial Agents & Chemotherapy,* 62**,** 12.

DUNLOP, P. S. M., CIAVOLA, M., RIZZO, L., MCDOWELL, D. A. & BYRNE, J. A. 2015. Effect of photocatalysis on the transfer of antibiotic resistance genes in urban wastewater. *Catalysis Today,* 240**,** 55-60.

GARCIA-MIGURA, L., LIEBANA, E., JENSEN, L. B., BARNES, S. & PLEYDELL, E. 2007. A longitudinal study to assess the persistence of vancomycin-resistant Enterococcus faecium (VREF) on an intensive broiler farm in the United Kingdom. *FEMS Microbiology Letters,* 275**,** 319-25.

GARCIA-MIGURA, L., PLEYDELL, E., BARNES, S., DAVIES, R. H. & LIEBANA, E. 2005. Characterization of vancomycin-resistant Enterococcus faecium isolates from broiler poultry and pig farms in England and Wales. *Journal of Clinical Microbiology,* 43**,** 3283-9.

HORTON, R. A., WU, G., SPEED, K., KIDD, S., DAVIES, R., COLDHAM, N. G. & DUFF, J. P. 2013. Wild birds carry similar Salmonella enterica serovar Typhimurium strains to those found in domestic animals and livestock. *Research in Veterinary Science,* 95**,** 45-48.

IGNASIAK, K. & MAXWELL, A. 2018. Oxytetracycline reduces the diversity of tetracycline-resistance genes in the Galleria mellonella gut microbiome. *BMC Microbiology,* 18**,** 228.

LIAO, X., CULLEN, P. J., LIU, D., MUHAMMAD, A. I., CHEN, S., YE, X., WANG, J. & DING, T. 2018. Combating Staphylococcus aureus and its methicillin resistance gene (mecA) with cold plasma. *Science of the Total Environment,* 645**,** 1287-1295.

MATHER, A. E., DENWOOD, M. J., HAYDON, D. T., MATTHEWS, L., MELLOR, D. J., COIA, J. E., BROWN, D. J. & REID, S. W. 2011. The prevalences of Salmonella Genomic Island 1 variants in human and animal Salmonella Typhimurium DT104 are distinguishable using a Bayesian approach. *PLoS ONE [Electronic Resource],* 6**,** e27220.

MEDANEY, F., ELLIS, R. J. & RAYMOND, B. 2016. Ecological and genetic determinants of plasmid distribution in Escherichia coli. *Environmental Microbiology,* 18**,** 4230-4239.

PORMOHAMMAD, A., NASIRI, M. J. & AZIMI, T. 2019. Prevalence of antibiotic resistance in Escherichia coli strains simultaneously isolated from humans, animals, food, and the environment: a systematic review and meta-analysis. *Infection and drug resistance,* 12**,** 1181-1197.

SHARIFUZZAMAN, S. M., RAHMAN, H., AUSTIN, D. A. & AUSTIN, B. 2018. Properties of Probiotics Kocuria SM1 and Rhodococcus SM2 Isolated from Fish Guts. *Probiotics and Antimicrobial Proteins,* 10**,** 534-542.

XU, Z., SHAH, H. N., MISRA, R., CHEN, J., ZHANG, W., LIU, Y., CUTLER, R. R. & MKRTCHYAN, H. V. 2018. The prevalence, antibiotic resistance and mecA characterization of coagulase negative staphylococci recovered from non-healthcare settings in London, UK. *Antimicrobial Resistance & Infection Control,* 7**,** 73.

1. **Not UK (64)**

ABDELLRAZEQ, G. S., KAMAR, A. M. & EL-HOUSHY, S. M. 2014. Molecular characterization of Listeria species isolated from frozen fish. *Alexandria Journal of Veterinary Sciences,* 40**,** 1-15.

AGNEW, A., WANG, J., FANNING, S., BEARHOP, S. & MCMAHON, B. J. 2015. Insights into antimicrobial resistance among long distance migratory East Canadian High Arctic light-bellied Brent geese (Branta bernicla hrota). *Irish Veterinary Journal,* 69**,** 13.

AGUNOS, A., WADDELL, L., LÉGER, D. & TABOADA, E. 2014. A systematic review characterizing on-farm sources of Campylobacter spp. for broiler chickens. *PloS one,* 9**,** e104905.

BANDELJ, P., GOLOB, M., OCEPEK, M., ZDOVC, I. & VENGUST, M. 2017-05. Antimicrobial Susceptibility Patterns of Clostridium difficile Isolates from Family Dairy Farms. v. 64.

BOLTON, D. J., IVORY, C. & MCDOWELL, D. 2013. A small study of Yersinia enterocolitica in pigs from birth to carcass andcharacterisation of porcine and human strains. *Food Control,* 33**,** 521-524.

BOLTON, D. J., IVORY, C. & MCDOWELL, D. 2013. A study of Salmonella in pigs from birth to carcass: Serotypes, genotypes, antibiotic resistance and virulence profiles. *International Journal of Food Microbiology,* 160**,** 298-303.

BUELOW, E., BAYJANOV, J. R., MAJOOR, E., WILLEMS, R. J., BONTEN, M. J., SCHMITT, H. & VAN SCHAIK, W. 2018. Limited influence of hospital wastewater on the microbiome and resistome of wastewater in a community sewerage system. *FEMS Microbiology Ecology,* 94**,** 01.

BURCH, T. R., SADOWSKY, M. J. & LAPARA, T. M. 2017. Effect of Different Treatment Technologies on the Fate of Antibiotic Resistance Genes and Class 1 Integrons when Residual Municipal Wastewater Solids are Applied to Soil. *Environmental Science & Technology,* 51**,** 14225-14232.

CACACE, D., FATTA-KASSINOS, D., MANAIA, C. M., CYTRYN, E., KREUZINGER, N., RIZZO, L., KARAOLIA, P., SCHWARTZ, T., ALEXANDER, J., MERLIN, C., GARELICK, H., SCHMITT, H., DE VRIES, D., SCHWERMER, C. U., MERIC, S., OZKAL, C. B., PONS, M. N., KNEIS, D. & BERENDONK, T. U. 2019. Antibiotic resistance genes in treated wastewater and in the receiving water bodies: A pan-European survey of urban settings. *Water Research,* 162**,** 320-330.

CARROLL, D., WANG, J., FANNING, S. & MCMAHON, B. J. 2015. Antimicrobial Resistance in Wildlife: Implications for Public Health. *Zoonoses & Public Health,* 62**,** 534-42.

CLEARY, D. W., BISHOP, A. H., ZHANG, L., TOPP, E., WELLINGTON, E. M. & GAZE, W. H. 2016. Long-term antibiotic exposure in soil is associated with changes in microbial community structure and prevalence of class 1 integrons. *FEMS Microbiology Ecology,* 92**,** 10.

COERTZE, R. D. & BEZUIDENHOUT, C. C. 2019. Global distribution and current research of AmpC beta-lactamase genes in aquatic environments: A systematic review. *Environmental Pollution,* 252**,** 1633-1642.

CUI, E. P., GAO, F., LIU, Y., FAN, X. Y., LI, Z. Y., DU, Z. J., HU, C. & NEAL, A. L. 2018. Amendment soil with biochar to control antibiotic resistance genes under unconventional water resources irrigation: Proceed with caution. *Environmental Pollution,* 240**,** 475-484.

DARWISH, W. S., ATIA, A. S., REDA, L. M., ELHELALY, A. E., THOMPSON, L. A. & ELDIN, W. F. S. 2018. Chicken giblets and wastewater samples as possible sources of methicillin-resistant Staphylococcus aureus: Prevalence, enterotoxin production, and antibiotic susceptibility. *Journal of Food Safety,* 38**,** 7.

DEMANÈCHE, S., SANGUIN, H., POTÉ, J., NAVARRO, E., BERNILLON, D., MAVINGUI, P., WILDI, W., VOGEL, T. M. & SIMONET, P. 2008. Antibiotic-resistant soil bacteria in transgenic plant fields. *Proceedings of the National Academy of Sciences of the United States of America,* 105**,** 3957-3962.

EKWANZALA, M. D., DEWAR, J. B., KAMIKA, I. & MOMBA, M. N. B. 2018. Systematic review in South Africa reveals antibiotic resistance genes shared between clinical and environmental settings. *Infection and drug resistance,* 11**,** 1907-1920.

ELBEDIWI, M., LI, Y., PAUDYAL, N., PAN, H., LI, X., XIE, S., RAJKOVIC, A., FENG, Y., FANG, W., RANKIN, S. C. & YUE, M. 2019. Global Burden of Colistin-Resistant Bacteria: Mobilized Colistin Resistance Genes Study (1980-2018). *Microorganisms,* 7.

ENGEMANN, C. A., KEEN, P. L., KNAPP, C. W., HALL, K. J. & GRAHAM, D. W. 2008. Fate of tetracycline resistance genes in aquatic systems: Migration from the water column to peripheral biofilms. *Environmental Science & Technology,* 42**,** 5131-5136.

ERAMO, A., MORALES MEDINA, W. R. & FAHRENFELD, N. L. 2019. Viability-based quantification of antibiotic resistance genes and human fecal markers in wastewater effluent and receiving waters. *Science of the Total Environment,* 656**,** 495-502.

FRICKE, W. F., WRIGHT, M. S., LINDELL, A. H., HARKINS, D. M., BAKER-AUSTIN, C., RAVEL, J. & STEPANAUSKAS, R. 2008. Insights into the environmental resistance gene pool from the genome sequence of the multidrug-resistant environmental isolate Escherichia coli SMS-3-5. *Journal of Bacteriology,* 190**,** 6779-6794.

GAO, P., MUNIR, M. & XAGORARAKI, I. 2012. Correlation of tetracycline and sulfonamide antibiotics with corresponding resistance genes and resistant bacteria in a conventional municipal wastewater treatment plant. *Science of the Total Environment,* 421-422**,** 173-183.

HADDADIN, R. N., ASSAF, A. M., HOMSI, A., COLLIER, P. J. & SHEHABI, A. 2019. Investigating possible association between multidrug resistance and isolate origin with some virulence factors of Escherichia coli strains isolated from infant faeces and fresh green vegetables. *Journal of Applied Microbiology,* 127**,** 88-98.

HARRIS, S., CORMICAN, M. & CUMMINS, E. 2012. The effect of conventional wastewater treatment on the levels of antimicrobial-resistant bacteria in effluent: a meta-analysis of current studies. *Environmental geochemistry and health,* 34**,** 749-62.

HENDRIKSEN, R. S., MUNK, P., NJAGE, P., VAN BUNNIK, B., MCNALLY, L., LUKJANCENKO, O., RODER, T., NIEUWENHUIJSE, D., PEDERSEN, S. K., KJELDGAARD, J., KAAS, R. S., CLAUSEN, P., VOGT, J. K., LEEKITCHAROENPHON, P., VAN DE SCHANS, M. G. M., ZUIDEMA, T., DE RODA HUSMAN, A. M., RASMUSSEN, S., PETERSEN, B., GLOBAL SEWAGE SURVEILLANCE PROJECT, C., AMID, C., COCHRANE, G., SICHERITZ-PONTEN, T., SCHMITT, H., ALVAREZ, J. R. M., AIDARA-KANE, A., PAMP, S. J., LUND, O., HALD, T., WOOLHOUSE, M., KOOPMANS, M. P., VIGRE, H., PETERSEN, T. N. & AARESTRUP, F. M. 2019. Global monitoring of antimicrobial resistance based on metagenomics analyses of urban sewage. *Nature communications,* 10**,** 1124.

HÖLZEL, C. S., SCHWAIGER, K., HARMS, K., KÜCHENHOFF, H., KUNZ, A., MEYER, K., MÜLLER, C. & BAUER, J. 2010. Sewage sludge and liquid pig manure as possible sources of antibiotic resistant bacteria. *Environmental Research,* 110**,** 318-326.

ICGEN, B. 2016. VanA-Type MRSA (VRSA) Emerged in Surface Waters. *Bulletin of Environmental Contamination & Toxicology,* 97**,** 359-366.

KIZNY GORDON, A. E., MATHERS, A. J., CHEONG, E. Y. L., GOTTLIEB, T., KOTAY, S., WALKER, A. S., PETO, T. E. A., CROOK, D. W. & STOESSER, N. 2017. The Hospital Water Environment as a Reservoir for Carbapenem-Resistant Organisms Causing Hospital-Acquired Infections-A Systematic Review of the Literature. *Clinical Infectious Diseases,* 64**,** 1435-1444.

KOCZURA, R., MOKRACKA, J., JABŁOŃSKA, L., GOZDECKA, E., KUBEK, M. & KAZNOWSKI, A. 2012. Antimicrobial resistance of integron-harboring Escherichia coli isolates from clinical samples, wastewater treatment plant and river water. *Science of the Total Environment,* 414**,** 680-685.

KOIKE, S., AMINOV, R. I., YANNARELL, A. C., GANS, H. D., KRAPAC, I. G., CHEE-SANFORD, J. C. & MACKIE, R. I. 2010. Molecular Ecology Of Macrolide-Lincosamide-Streptogramin B Methylases in Waste Lagoons and Subsurface Waters Associated with Swine Production. *Microbial Ecology,* 59**,** 487-498.

KOIKE, S., KRAPAC, I. G., OLIVER, H. D., YANNARELL, A. C., CHEE-SANFORD, J. C., AMINOV, R. I. & MACKIE, R. I. 2007. Monitoring and source tracking of tetracycline resistance genes in lagoons and groundwater adjacent to swine production facilities over a 3-year period. *Applied and Environmental Microbiology,* 73**,** 4813-4823.

LE BRIS, H., DHAOUADI, R., NAVINER, M., GIRAUD, E., MANGION, C., ARMAND, F., LA COTTE, N. D., THORIN, C., GANIÈRE, J. P. & POULIQUEN, H. 2007. Experimental approach on the selection and persistence of anti-microbial-resistant Aeromonads in faecal matter of rainbow trout during and after an oxolinic acid treatment. v. 273 issue 4.

LIAO, H., FRIMAN, V. P., GEISEN, S., ZHAO, Q., CUI, P., LU, X., CHEN, Z., YU, Z. & ZHOU, S. 2019. Horizontal gene transfer and shifts in linked bacterial community composition are associated with maintenance of antibiotic resistance genes during food waste composting. *Science of the Total Environment,* 660**,** 841-850.

LIAO, H., LU, X., RENSING, C., FRIMAN, V. P., GEISEN, S., CHEN, Z., YU, Z., WEI, Z., ZHOU, S. & ZHU, Y. 2018. Hyperthermophilic Composting Accelerates the Removal of Antibiotic Resistance Genes and Mobile Genetic Elements in Sewage Sludge. *Environmental Science & Technology,* 52**,** 266-276.

LIAO, H., ZHAO, Q., CUI, P., CHEN, Z., YU, Z., GEISEN, S., FRIMAN, V. P. & ZHOU, S. 2019. Efficient reduction of antibiotic residues and associated resistance genes in tylosin antibiotic fermentation waste using hyperthermophilic composting. *Environment International,* 133.

LIU, Y., CUI, E., NEAL, A. L., ZHANG, X., LI, Z., XIAO, Y., DU, Z., GAO, F., FAN, X. & HU, C. 2019. Reducing water use by alternate-furrow irrigation with livestock wastewater reduces antibiotic resistance gene abundance in the rhizosphere but not in the non-rhizosphere. *Science of the Total Environment,* 648**,** 12-24.

MACKIE, R. I., KOIKE, S., KRAPAC, I., CHEE-SANFORD, J., MAXWELL, S. & AMINOV, R. I. 2006. Tetracycline residues and tetracycline resistance genes in groundwater impacted by swine production facilities. *Animal Biotechnology,* 17**,** 157-176.

MAHON, B. M., BREHONY, C., CAHILL, N., MCGRATH, E., O'CONNOR, L., VARLEY, A., CORMICAN, M., RYAN, S., HICKEY, P., KEANE, S., MULLIGAN, M., RUANE, B., JOLLEY, K. A., MAIDEN, M. C., BRISSE, S. & MORRIS, D. 2019. Detection of OXA-48-like-producing Enterobacterales in Irish recreational water. *Science of the Total Environment,* 690**,** 1-6.

MENEGHINE, A. K., NIELSEN, S., VARANI, A. M., THOMAS, T. & ALVES, L. M. C. 2017. Metagenomic analysis of soil and freshwater from zoo agricultural area with organic fertilization. *PLoS ONE,* 12.

MORACH, M., STEPHAN, R., SCHMITT, S., EWERS, C., ZSCHÖCK, M., REYES-VELEZ, J., GILLI, U., DEL PILAR CRESPO-ORTIZ, M., CRUMLISH, M., GUNTURU, R., DAUBENBERGER, C. A., IP, M., REGLI, W. & JOHLER, S. 2018. Population structure and virulence gene profiles of Streptococcus agalactiae collected from different hosts worldwide. *European Journal of Clinical Microbiology and Infectious Diseases,* 37**,** 527-536.

MOREIRA, N. F. F., NARCISO-DA-ROCHA, C., POLO-LOPEZ, M. I., PASTRANA-MARTINEZ, L. M., FARIA, J. L., MANAIA, C. M., FERNANDEZ-IBANEZ, P., NUNES, O. C. & SILVA, A. M. T. 2018. Solar treatment (H2O2, TiO2-P25 and GO-TiO2 photocatalysis, photo-Fenton) of organic micropollutants, human pathogen indicators, antibiotic resistant bacteria and related genes in urban wastewater. *Water Research,* 135**,** 195-206.

MRIDHA, M. A. R., NAREJO, N. T., UDDIN, M. S., KABIR, M. S., KARIM, M. & CHOWDHURY, M. B. R. 2005. Resistance of Aeromonas spp. in the fish, Catla catla, against some antibacterial agents. *Pakistan Journal of Zoology,* 37**,** 158-161.

MUNK, P., KNUDSEN, B. E., LUKJANCENKO, O., DUARTE, A. S. R., VAN GOMPEL, L., LUIKEN, R. E. C., SMIT, L. A. M., SCHMITT, H., GARCIA, A. D., HANSEN, R. B., PETERSEN, T. N., BOSSERS, A., RUPPE, E., GROUP, E., LUND, O., HALD, T., PAMP, S. J., VIGRE, H., HEEDERIK, D., WAGENAAR, J. A., MEVIUS, D. & AARESTRUP, F. M. 2018. Abundance and diversity of the faecal resistome in slaughter pigs and broilers in nine European countries. *Nature Microbiology,* 3**,** 898-908.

NORONHA, M. F., LACERDA JÚNIOR, G. V., GILBERT, J. A. & DE OLIVEIRA, V. M. 2017. Taxonomic and functional patterns across soil microbial communities of global biomes. *Science of the Total Environment,* 609**,** 1064-1074.

PARNANEN, K. M. M., NARCISO-DA-ROCHA, C., KNEIS, D., BERENDONK, T. U., CACACE, D., THI THUY, D., ELPERS, C., FATTA-KASSINOS, D., HENRIQUES, I., JAEGER, T., KARKMAN, A., MARTINEZ, J. L., MICHAEL, S. G., MICHAEL-KORDATOU, I., O'SULLIVAN, K., RODRIGUEZ-MOZAZ, S., SCHWARTZ, T., SHENG, H., SORUM, H., STEDTFELD, R. D., TIEDJE, J. M., GIUSTINA, S. V. D., WALSH, F., VAZ-MOREIRA, I., VIRTA, M. & MANAIA, C. M. 2019. Antibiotic resistance in European wastewater treatment plants mirrors the pattern of clinical antibiotic resistance prevalence. *Science Advances,* 5.

PENG, S., DOLFING, J., FENG, Y., WANG, Y. & LIN, X. 2018. Enrichment of the Antibiotic Resistance Gene tet(L) in an Alkaline Soil Fertilized With Plant Derived Organic Manure. *Frontiers in Microbiology,* 9**,** 1140.

PEREIRA, G. D. V., DA CUNHA, D. G., PEDREIRA MOURINO, J. L., RODILES, A., JARAMILLO-TORRES, A. & MERRIFIELD, D. L. 2017. Characterization of microbiota in Arapaima gigas intestine and isolation of potential probiotic bacteria. *Journal of Applied Microbiology,* 123**,** 1298-1311.

RADHOUANI, H., IGREJAS, G., PINTO, L., GONÇALVES, A., COELHO, C., RODRIGUES, J. & POETA, P. 2011. Molecular characterization of antibiotic resistance in enterococci recovered from seagulls (Larus cachinnans) representing an environmental health problem. *Journal of Environmental Monitoring,* 13**,** 2227-2233.

RAMSAY, K. A., WARDELL, S. J. T., PATRICK, W. M., BROCKWAY, B., REID, D. W., WINSTANLEY, C., BELL, S. C. & LAMONT, I. L. 2019. Genomic and phenotypic comparison of environmental and patient-derived isolates of Pseudomonas aeruginosa suggest that antimicrobial resistance is rare within the environment. *Journal of Medical Microbiology,* 25**,** 25.

SAPKOTA, A. R., CURRIERO, F. C., GIBSON, K. E. & SCHWAB, K. J. 2007. Antibiotic-Resistant Enterococci and Fecal Indicators in Surface Water and Groundwater Impacted by a Concentrated Swine Feeding Operation. *Environmental Health Perspectives,* 115**,** 1040-1045.

SHEN, Y., XU, C., SUN, Q., SCHWARZ, S., OU, Y., YANG, L., HUANG, Z., EICHHORN, I., WALSH, T. R., WANG, Y., ZHANG, R. & SHEN, J. 2018. Prevalence and Genetic Analysis of mcr-3-Positive Aeromonas Species from Humans, Retail Meat, and Environmental Water Samples. *Antimicrobial Agents & Chemotherapy,* 62**,** 09.

SINGH, B., TYAGI, A., BILLEKALLU THAMMEGOWDA, N. K. & ANSAL, M. D. 2018-06. Prevalence and antimicrobial resistance of vibrios of human health significance in inland saline aquaculture areas. v. 49.

SOMENSI, C. A., SOUZA, A. L. F., SIMIONATTO, E. L., GASPARETO, P., MILLET, M. & RADETSKI, C. M. 2015. Genetic material present in hospital wastewaters: Evaluation of the efficiency of DNA denaturation by ozonolysis and ozonolysis/sonolysis treatments. *Journal of Environmental Management,* 162**,** 74-80.

STALDER, T., ALRHMOUN, M., LOUVET, J. N., CASELLAS, M., MAFTAH, C., CARRION, C., PONS, M. N., PAHL, O., PLOY, M. C. & DAGOT, C. 2013. Dynamic Assessment of the Floc Morphology, Bacterial Diversity, and Integron Content of an Activated Sludge Reactor Processing Hospital Effluent. *Environmental Science & Technology,* 47**,** 7909-7917.

TIMRAZ, K., XIONG, Y. H., AL QARNI, H. & HONG, P. Y. 2017. Removal of bacterial cells, antibiotic resistance genes and integrase genes by on-site hospital wastewater treatment plants: surveillance of treated hospital effluent quality. *Environmental Science-Water Research & Technology,* 3**,** 293-303.

TOMOVA, A., IVANOVA, L., BUSCHMANN, A. H., GODFREY, H. P. & CABELLO, F. C. 2018-01. Plasmid-Mediated Quinolone Resistance (PMQR) Genes and Class 1 Integrons in Quinolone-Resistant Marine Bacteria and Clinical Isolates of Escherichia coli from an Aquacultural Area. v. 75.

VELDMAN, K., CAVACO, L. M., MEVIUS, D., BATTISTI, A., FRANCO, A., BOTTELDOORN, N., BRUNEAU, M., PERRIN-GUYOMARD, A., CERNY, T., DE FRUTOS ESCOBAR, C., GUERRA, B., SCHROETER, A., GUTIERREZ, M., HOPKINS, K., MYLLYNIEMI, A. L., SUNDE, M., WASYL, D. & AARESTRUP, F. M. 2011. International collaborative study on the occurrence of plasmid-mediated quinolone resistance in Salmonella enterica and Escherichia coli isolated from animals, humans, food and the environment in 13 European countries. *Journal of Antimicrobial Chemotherapy,* 66**,** 1278-1286.

WU, D., HUANG, X. H., SUN, J. Z., GRAHAM, D. W. & XIE, B. 2017. Antibiotic Resistance Genes and Associated Microbial Community Conditions in Aging Landfill Systems. *Environmental Science & Technology,* 51**,** 12859-12867.

XIANG, Q., ZHU, D., GILES, M., NEILSON, R., YANG, X. R., QIAO, M. & CHEN, Q. L. 2019. Agricultural activities affect the pattern of the resistome within the phyllosphere microbiome in peri-urban environments. *Journal of Hazardous Materials,* 382**,** 121068.

YANG, Y., SONG, W., LIN, H., WANG, W., DU, L. & XING, W. 2018. Antibiotics and antibiotic resistance genes in global lakes: A review and meta-analysis. *Environment International,* 116**,** 60-73.

ZHANG, J., LIN, H., MA, J., SUN, W., YANG, Y. & ZHANG, X. 2019. Compost-bulking agents reduce the reservoir of antibiotics and antibiotic resistance genes in manures by modifying bacterial microbiota. *Science of the Total Environment,* 649**,** 396-404.

ZHANG, Y., MARRS, C. F., SIMON, C. & XI, C. 2009. Wastewater treatment contributes to selective increase of antibiotic resistance among Acinetobacter spp. *Science of the Total Environment,* 407**,** 3702-3706.

ZHAO, F., FENG, Y., LU, X., MCNALLY, A. & ZONG, Z. 2017. IncP Plasmid Carrying Colistin Resistance Gene mcr-1 in Klebsiella pneumoniae from Hospital Sewage. *Antimicrobial Agents & Chemotherapy,* 61**,** 02.

ZHENG, F., ZHU, D., GILES, M., DANIELL, T., NEILSON, R., ZHU, Y. G. & YANG, X. R. 2019. Mineral and organic fertilization alters the microbiome of a soil nematode Dorylaimus stagnalis and its resistome. *Science of the Total Environment,* 680**,** 70-78.

ZOTHANPUIA, PASSARI, A. K., GUPTA, V. K. & SINGH, B. P. 2016. Detection of antibiotic-resistant bacteria endowed with antimicrobial activity from a freshwater lake and their phylogenetic affiliation. *PeerJ,* 4**,** e2103.

1. **Outcome (5)**

BISHOP, A. H., RACHWAL, P. A. & VAID, A. 2014. Identification of genes required by Bacillus thuringiensis for survival in soil by transposon-directed insertion site sequencing. *Current Microbiology,* 68**,** 477-85.

EBDON, J. E. & TAYLOR, H. D. 2006. Geographical stability of enterococcal antibiotic resistance profiles in Europe and its implications for the identification of fecal sources. *Environmental Science & Technology,* 40**,** 5327-32.

GOSLING, R. J., MUELLER-DOBLIES, D., MARTELLI, F., NUNEZ-GARCIA, J., KELL, N., RABIE, A., WALES, A. D. & DAVIES, R. H. 2018. Observations on the distribution and persistence of monophasic Salmonella Typhimurium on infected pig and cattle farms. *Veterinary Microbiology,* 227**,** 90-96.

SALIMRAJ, R., ZHANG, L., HINCHLIFFE, P., WELLINGTON, E. M. H., BREM, J., SCHOFIELD, C. J., GAZE, W. H. & SPENCER, J. 2016. Structural and biochemical characterization of Rm3, a subclass B3 metallo-β-lactamase identified from a functional metagenomic study. *Antimicrobial Agents and Chemotherapy,* 60**,** 5828-5840.

TERRA, L., DYSON, P. J., HITCHINGS, M. D., THOMAS, L., ABDELHAMEED, A., BANAT, I. M., GAZZE, S. A., VUJAKLIJA, D., FACEY, P. D., FRANCIS, L. W. & QUINN, G. A. 2018. A Novel Alkaliphilic Streptomyces Inhibits ESKAPE Pathogens. *Frontiers in Microbiology,* 9**,** 2458.

1. **Study design (15)**

ARNOLD, K. E., WILLIAMS, N. J. & BENNETT, M. 2016. 'Disperse abroad in the land': the role of wildlife in the dissemination of antimicrobial resistance. *Biology Letters,* 12**,** 08.

BRANGER, C., LEDDA, A., BILLARD-POMARES, T., DOUBLET, B., FOUTEAU, S., BARBE, V., ROCHE, D., CRUVEILLER, S., MEDIGUE, C., CASTELLANOS, M., DECRE, D., DRIEUX-ROUZE, L., CLERMONT, O., GLODT, J., TENAILLON, O., CLOECKAERT, A., ARLET, G. & DENAMUR, E. 2018. Extended-spectrum beta-lactamase-encoding genes are spreading on a wide range of Escherichia coli plasmids existing prior to the use of third-generation cephalosporins. *Microbial Genomics,* 4**,** 16.

BROWNING, L. M., REILLY, W. J., COIA, J. E., MATHER, H. & BROWN, D. J. 2005. Antimicrobial resistance of Salmonella in Scotland, 2004 (excluding Typhi and Paratyphi). *HPS Weekly Report,* 39**,** 268-272.

CHEE-SANFORD, J., MAXWELL, S., TSAU, K., MERRICK, K. & AMINOV, R. 2011. Antibiotic Resistance in Swine-Manure-Impacted Environments. *Antimicrobial Resistance in the Environment.* John Wiley and Sons.

DESTIANI, R. & TEMPLETON, M. R. 2019. Chlorination and ultraviolet disinfection of antibiotic-resistant bacteria and antibiotic resistance genes in drinking water. *Aims Environmental Science,* 6**,** 222-241.

FINLEY, R. L., COLLIGNON, P., LARSSON, D. G. J., MCEWEN, S. A., LI, X. Z., GAZE, W. H., REID-SMITH, R., TIMINOUNI, M., GRAHAM, D. W. & TOPP, E. 2013. The Scourge of Antibiotic Resistance: The Important Role of the Environment. *Clinical Infectious Diseases,* 57**,** 704-710.

GRAHAM, D. W. 2015. Antibiotic Resistance in the Environment: Not the Usual Suspects. *Chemistry & Biology,* 22**,** 805-6.

GREIG, J., RAJIĆ, A., YOUNG, I., MASCARENHAS, M., WADDELL, L. & LEJEUNE, J. 2015. A scoping review of the role of wildlife in the transmission of bacterial pathogens and antimicrobial resistance to the food Chain. *Zoonoses and public health,* 62**,** 269-84.

KHAN, S., BEATTIE, T. K. & KNAPP, C. W. 2019. Rapid selection of antimicrobial-resistant bacteria in complex water systems by chlorine and pipe materials. *Environmental Chemistry Letters,* 17**,** 1367-1373.

LASKARIS, P., GAZE, W. H. & WELLINGTON, E. M. H. 2011. Environmental reservoirs of resistance genes in antibiotic-producing bacteria and their possible impact on the evolution of antibiotic resistance. *Antimicrobial resistance in the environment*.

RODGERS, K., MCLELLAN, I., PESHKUR, T., WILLIAMS, R., TONNER, R., HURSTHOUSE, A. S., KNAPP, C. W. & HENRIQUEZ, F. L. 2019. Can the legacy of industrial pollution influence antimicrobial resistance in estuarine sediments? *Environmental Chemistry Letters,* 17**,** 595-607.

SHAW, L. M., BLANCHARD, A., CHEN, Q., AN, X., DAVIES, P., TOTEMEYER, S., ZHU, Y. G. & STEKEL, D. J. 2019. DirtyGenes: testing for significant changes in gene or bacterial population compositions from a small number of samples. *Scientific Reports,* 9**,** 2373.

TELLO GILDEMEISTER, A. A. 2012. *A study into the effects and environmental risk of antibiotics used in freshwater aquaculture on environmental bacteria.* [Great Britain] : University of Stirling, 2012.

VENGLOVSKY, J., SASAKOVA, N. & PLACHA, I. 2009. Pathogens and antibiotic residues in animal manures and hygienic and ecological risks related to subsequent land application. v. v. 100.

WILLIAMS-NGUYEN, J., SALLACH, J. B., BARTELT-HUNT, S., BOXALL, A. B., DURSO, L. M., MCLAIN, J. E., SINGER, R. S., SNOW, D. D. & ZILLES, J. L. 2016. Antibiotics and Antibiotic Resistance in Agroecosystems: State of the Science. *Journal of Environmental Quality,* 45**,** 394-406.
